# HIV broadly neutralizing antibody precursors to the Apex epitope induced in non-human primates

**DOI:** 10.1126/sciimmunol.adt6660

**Published:** 2025-08-22

**Authors:** Krystal M. Ma, Henry J. Sutton, Payal P. Pratap, Jon M. Steichen, Diane Carnathan, James Quinn, Oleksandr Kalyuzhniy, Alessia Liguori, Sashank Agrawal, Sabyasachi Baboo, Patrick Madden, Christopher A. Cottrell, Jordan R. Willis, Jeong-Hyun Lee, Elise Landais, Xiaozhen Hu, Parham Ramezani-Rad, Gabriel Ozorowski, Vanessa R. Lewis, Jolene K. Diedrich, Xiaoya Zhou, Tasha K. Altheide, Nicole Phelps, Erik Georgeson, Nushin B. Alavi, Danny Lu, Saman Eskandarzadeh, Michael Kubitz, Yumiko Adachi, Tina-Marie Mullen, Murillo Silva, Mariane B. Melo, Sunny Himansu, Darrell J. Irvine, Dennis R. Burton, John R. Yates, James C. Paulson, Devin Sok, Ian A. Wilson, Guido Silvestri, Andrew B. Ward, Shane Crotty, William R. Schief

**Affiliations:** 1 Consortium for HIV/AIDS Vaccine Development (CHAVD), The Scripps Research Institute, La Jolla, CA 92037, USA; 2 IAVI Neutralizing Antibody Center, The Scripps Research Institute, La Jolla, CA 92037, USA; 3 Department of Immunology and Microbiology, The Scripps Research Institute, La Jolla, CA 92037, USA; 4 Center for Vaccine Innovation, La Jolla Institute for Immunology, La Jolla, CA 92037, USA; 5 Department of Integrative Structural and Computational Biology, The Scripps Research Institute, La Jolla, CA 92037, USA; 6 Division of Microbiology and Immunology, Emory National Primate Research Center, Emory University, Atlanta, GA 30329, USA; 7 Department of Pathology and Laboratory Medicine, Emory University School of Medicine, Atlanta, GA 30329, USA; 8 Department of Molecular Medicine, The Scripps Research Institute, La Jolla, CA 92037, USA; 9 Howard Hughes Medical Institute, Chevy Chase, MD, 20815, USA.; 10 Moderna Inc., Cambridge, MA, 02139, USA.; 11 David H. Koch Institute for Integrative Cancer Research, MIT, Cambridge, MA, 02139, USA.; 12 Department of Materials Science and Engineering, MIT, Cambridge, MA, 02139, USA.; 13 Department of Biological Engineering, MIT, Cambridge, MA, 02139, USA.; 14 Ragon Institute of Massachusetts General Hospital, Cambridge, MA, 02139, USA.; 15 Department of Medicine, Division of Infectious Diseases and Global Public Health, University of California, San Diego (UCSD), La Jolla, CA, 92037 USA

## Abstract

An effective prophylactic HIV vaccine will likely need to induce broadly neutralizing antibodies (bnAbs). BnAbs to the Apex region of the HIV envelope glycoprotein (Env) are promising targets for vaccination due to their relatively low somatic hypermutation compared with other bnAbs. Most Apex bnAbs engage Env using an exceptionally long heavy chain complementarity determining region 3 (HCDR3) containing specific binding motifs, which reduces bnAb precursor frequency and makes priming of rare bnAb precursors a likely limiting step in the path to Apex bnAb induction. We found that adjuvanted protein or mRNA-LNP immunization of rhesus macaques with ApexGT6, an Env trimer engineered to bind Apex bnAb precursors, consistently induced Apex bnAb-related precursors with long HCDR3s bearing bnAb-like sequence motifs. Cryo-electron microscopy revealed that elicited Apex bnAb-related HCDR3s possessed structures combining elements of several prototype Apex bnAbs. These results achieve an important HIV vaccine development milestone in outbred primates.

## One Sentence Summary

A germline-targeting trimer consistently induced rare bnAb-like precursors specific for the HIV Env Apex in rhesus macaques.

## Introduction

A primary goal for the HIV vaccine design field is to develop a vaccine that induces broadly neutralizing antibodies (bnAbs) that can protect against diverse HIV strains (1–4). One of the leading strategies proposed to achieve that goal, germline-targeting vaccine design, requires a priming immunogen to induce rare bnAb-precursor B cells that possess the genetic features needed to develop into bnAbs, followed by a series of heterologous boost immunogens to guide maturation to bnAb development (5–29). The IAVI G001 clinical trial demonstrated that a germline-targeting immunogen induced precursors for the VRC01 class of bnAbs specific for the CD4 binding site of HIV Env in 97% of vaccine recipients (19, 20). A vaccine that elicits VRC01-class bnAbs in humans has not yet been reported, as developing a sequential boosting regimen to guide bnAb-precursor B cells to acquire the mutations needed to become bnAbs remains a challenging and complex task (15, 24, 30). Furthermore, an optimal vaccine capable of inducing bnAbs to all or nearly all circulating HIV strains will require induction of multiple different classes of bnAbs specific for different HIV Env epitopic regions.

BnAbs specific for the Apex region of Env include some of the most potent bnAbs identified and typically have less somatic hypermutation (SHM) than bnAbs to other Env epitopes (31, 32), thus making them desirable targets for vaccination as they might theoretically be elicited by fewer sequential heterologous boosting steps than other bnAb classes. The primary hurdle to eliciting Apex bnAbs is believed to be the low frequency of bnAb-precursor B cells possessing a long (≥ 24 amino acid [aa]) and acidic HCDR3 that can penetrate the glycan shield and bind to the positively charged surface within the Apex epitopic region (18, 32–35). Among the long HCDR3 Apex bnAbs, PCT64 and PG9 were identified as having the highest and second-highest precursor frequencies in the human immunoglobulin repertoire (18). Furthermore, a germline-targeting Env trimer, ApexGT5, was shown to have affinity for the inferred precursors of both PCT64 and PG9 (18) and to be capable of priming PCT64-like responses in a mouse model (17). Rhesus macaques (RMs) have a relatively human-like naive B cell repertoire and the ability to produce long HCDR3s (36). A RM infected with a simian-human immunodeficiency virus (SHIV) produced an Apex epitope-specific bnAb, RHA1, similar to the PCT64 human bnAb (37). We sought to evaluate whether Apex bnAb-related precursors could be induced by vaccination in outbred primates that can produce polyclonal long HCDR3 responses, including not only bnAb precursors but also long HCDR3 competitors absent from mouse models. We designed a slightly improved germline-targeting trimer, ApexGT6, with affinity for additional PCT64- and PG9-class precursors, and we evaluated B cell responses to this trimer delivered as an adjuvanted soluble protein or mRNA-encoded membrane-anchored protein in RMs.

## Results

### Structure-guided directed evolution produced an improved Apex bnAb germline-targeting priming immunogen

**Fig. 1.**
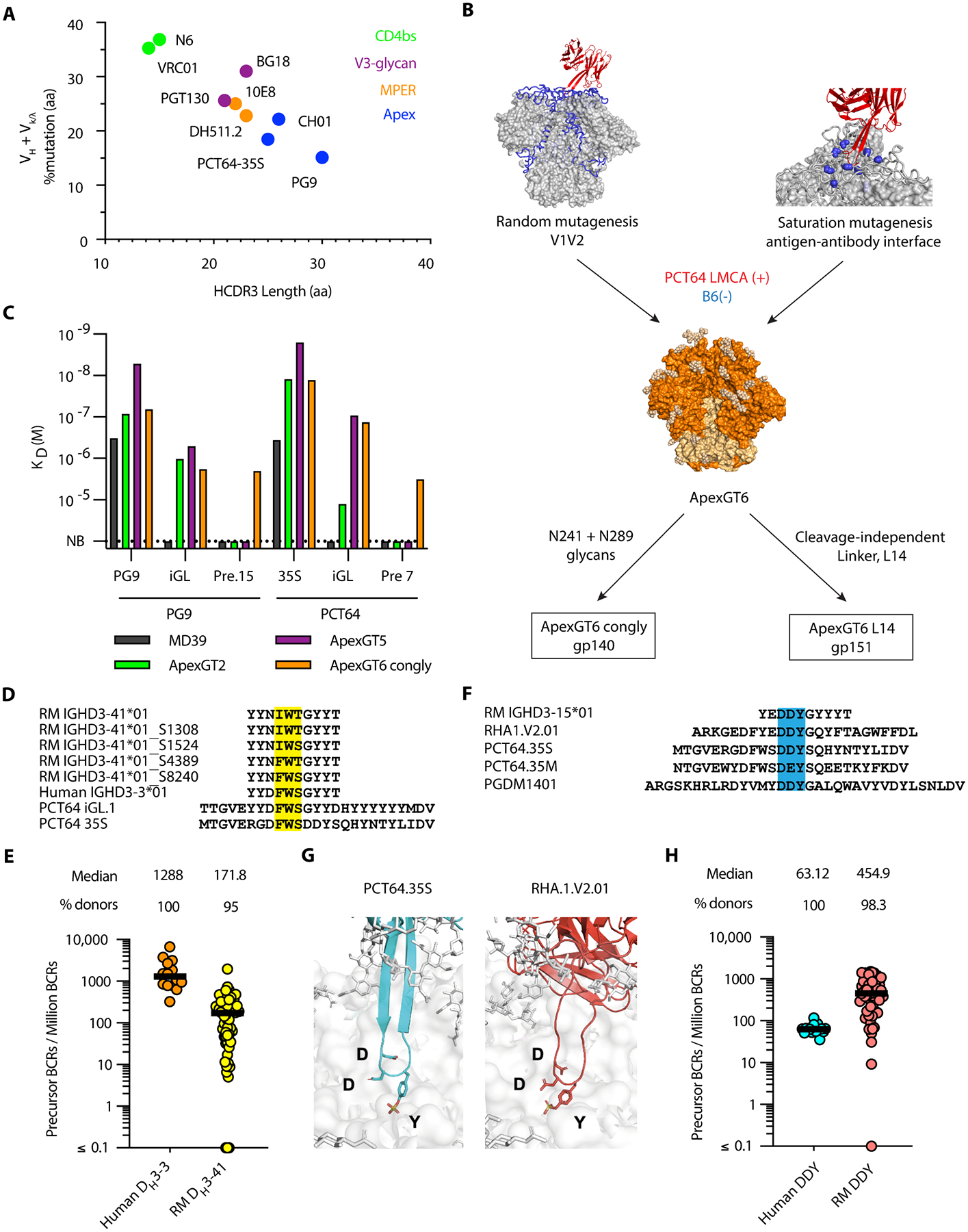
Structure-guided directed evolution produced ApexGT6 candidate immunogens to prime Apex bnAb-like precursor responses. **(A)** Somatic hypermutation of bnAbs versus their HCDR3 length. Somatic hypermutation was calculated by combining the number of mutations on the heavy and light chain variable regions (V_H_ and V_κ/λ_), then dividing by their combined length. All calculations were based on aa mutations. Each dot represents a bnAb target, colored by its directed epitope. **(B)** The immunogen design pathway starting with previously designed ApexGT trimers to ApexGT6 congly gp140 and ApexGT6 L14 gp151. The libraries were designed using the high-resolution cryoEM structure of ApexGT2.2MUT (displayed as a grey surface) in combination with PCT64 LMCA (depicted as a red cartoon). The mutations from each library are represented by blue ribbons or spheres. The enriched mutations were combined to produce ApexGT6 (surface representation, gp120 is colored orange, gp41 is taupe, and glycans are depicted as beige spheres). **(C)** Surface plasmon resonance (SPR) K_D_s for MD39 and ApexGT trimer analytes binding to PCT64 and PG9 variants as IgG ligands. Pre.15 is a PG9-like NGS precursor and Pre.7 is a PCT64-like NGS precursor. Both precursors were designed by incorporating an NGS-derived HCDR3 into the corresponding inferred germlines (iGLs). The data were fit using a 1:1 binding model. NB, no binding, is the highest concentration (approximately 10 μM) tested. **(D)** Alignment of RM IGHD3–41 alleles, human IGHD3–3, and HCDR3 sequences for one inferred germline (iGL.1) and one mature (PCT64.35S) of PCT64. The FWS motif are highlighted in yellow. **(E)** The frequency of PCT64-like HC sequences in 14 human donors and 60 RMs. Median was plotted across all donors. **(F)** Alignment of RM IGHD3–15 and HCDR3 sequences for RHA1 (labelled as RHA1.V2.01), two mature PCT64s (PCT64.35S and PCT64.35M) and PGDM1401. The DDY motif are highlighted in cyan. **(G)** Zoomed-in views of PCT64 (cyan) and RHA1 (red) HCDR3s buried within the ApexGT2 and BG505 DS-SOSIP trimers, respectively. The DDY motifs are shown as sticks. **(H)** The frequency of Apex bnAb-like heavy chain (HC) sequences (with a DDY motif around the middle of the long HCDR3) in 14 human donors and 60 RMs. Median was plotted across all donors. Source data can be found in Data file S4.

Compared to other HIV bnAbs, Apex bnAbs typically require fewer SHMs but longer HCDR3s (Fig. 1A). This poses a challenge for effective precursor priming, as the precursor B cell frequency is low (18). To address this, we prioritized PCT64, which has the highest precursor frequency, to guide the design of an improved Apex bnAb germline-targeting trimer. We used structure-guided directed evolution by mammalian cell-surface display to iteratively engineer ApexGT trimers (12, 18). We constructed two libraries: a random mutagenesis library on V1V2 using error-prone PCR, and a combinatorial saturation mutagenesis library targeting residues identified within the PCT64 antigen-antibody binding interface (18). We used the least mutated common ancestor of PCT64 (PCT64 LMCA) as the positive probe and the non-nAb B6 as the negative probe to select mutations that enhanced affinity to the germline while still maintaining good trimerization (Fig. 1B). Mutations identified from enriched clones were incorporated into ApexGT trimers for affinity evaluation using surface plasmon resonance (SPR) and antigenic profiling by enzyme-linked immunosorbent assay (ELISA). Our best candidate from this round of design, ApexGT6, differed from the previously published design, ApexGT5, by three mutations (Table S1). These mutations were introduced to gain affinity for PCT64 and PG9 precursors identified bioinformatically in next-generation sequencing (NGS) data for HIV seronegative individuals (18) (Fig. 1C and fig. S1, A and B), as well as to stabilize the prefusion state and thereby improved the antigenic profile (fig. S1C). To minimize off-target responses to glycan holes outside of the apex, we developed a glycan hole-filled version of the soluble protein trimer of ApexGT6, known as ApexGT6 congly, with N241 and N289 glycosylation sites introduced (Fig. 1B and fig. S1D). Additionally, we constructed a cleavage-independent version of ApexGT6, referred to as ApexGT6 L14, that can be expressed as a membrane-bound trimer (gp151) to facilitate mRNA-LNP immunization (Fig. 1B). Both ApexGT6 congly and L14 exhibited the antigenic profile of a well-folded Env trimer with good thermostability (fig. S1, C and E), as well as good occupancy at the highly conserved glycosylation sites within the apex (38) (fig. S1D).

### Non-human primates are relevant pre-clinical models for Apex vaccine development

ApexGT6 was designed with the aim of priming PCT64- and PG9-like responses in humans (18). However, we hypothesized that similar responses could be elicited in RM due to their genetic similarity to humans and their ability to produce long HCDR3s (36). Moreover, Roark et al. (37) showed that a SHIV-infected RM developed PCT64-like responses with similar patterns of Env-antibody coevolution to humans.

RMs have a germline D gene (D_H_3–41) with several alleles similar to the human D_H_3–3 (27) (Fig. 1D). To compare frequencies of PCT64-like heavy chain (HC) precursors that utilize D_H_3–41 in RMs or D_H_3–3 in humans, we conducted bioinformatic searches in ultra-deep immunoglobulin HC sequencing datasets from humans (16) and RMs (25). In these searches, PCT64-like precursors were defined by having: (1) HCDR3 length ≥ 24 aa (1 aa shorter than PCT64); (2) HCDR3 encoded by D_H_3–41 or D_H_3–3 using the same reading frame as PCT64; and (3) the start position of the D gene occurred approximately in the middle of the HCDR3, at least 6 aa from the beginning and 12 aa from the end, based on the position in PCT64 (fig. S1F). These definitions allowed for precursors with exceptionally long HCDR3s but with diverse V-D and D-J junctions. We detected such precursors in 57 of 60 RMs (95%) and 14 of 14 humans (Fig. 1E). Among RMs with detectable D_H_3–41-based PCT64-like HC precursors, the frequency of such precursors (median 171.8 per million) was 7.5-fold lower than the frequency of D_H_3–3-based PCT64-like precursors in humans (median 1,288 per million) (Fig. 1E). These results suggested that RM vaccine priming of D_H_3–41-based PCT64-like responses might be more challenging than human vaccine priming of D_H_3–3-based PCT64-like responses.

RMs possess another germline D gene, D_H_3–15, that has promise to support PCT64-like responses but does not have a human counterpart. The D_H_3–15 gene is used by RHA1 (37), the only known Apex epitope-specific bnAb isolated from a RM. D_H_3–15 directly encodes a DDY motif that is present at the tip of the long HCDR3s of RHA1, as well as PCT64, and another human Apex bnAb, PGDM1401 (39) (Fig. 1F). Structural studies of these bnAbs show that DDY includes a sulfated tyrosine (40) and makes key interactions with Env (18, 37) (Fig. 1G). The DDY in the human bnAbs most likely resulted from a V(D)J recombination event or was acquired during lineage development through somatic hypermutation (SHM) (41), unlike the DDY in RHA1 germline-encoded by D_H_3–15.

Using RHA1, PCT64, and PGDM1401 as guides, we searched for a more general class of Apex bnAb-related precursors based on the following criteria: (1) HCDR3 length ≥ 24 aa; and (2) the DDY motif occurred approximately in the middle of the HCDR3, at least 7 aa from the beginning and 10 aa from the end, based on the position in PCT64 and RHA1 (fig. S1F). These definitions allowed for precursors with exceptionally long HCDR3s possessing a DDY motif, regardless of germline gene usage, with diverse V-D and D-J junctions. Although humans lack a germline D gene that can directly encode DDY on the HCDR3 (fig. S1G), we hypothesized that DDY could occur in human HCDR3s in the V-D or D-J junctions. We detected such precursors in 59 of 60 RMs (98.3%) and 14 of 14 humans (Fig. 1H). The frequency of such precursors was 7.2-fold higher in RMs (median 454.9 per million) than in humans (median 63.12 per million) (Fig. 1H), likely owing to the DDY-encoding D_H_3–15 gene in RMs for which humans have no counterpart. These results suggested that it might be easier to elicit Apex bnAb-related DDY-encoded responses in RMs than in humans. Nevertheless, we found it encouraging that such precursors exist in both humans and RMs at substantial frequency.

We conducted similar searches for PG9-like precursors defined by having: (1) HCDR3 length ≥ 29 aa (1 aa shorter than PG9); (2) HCDR3 encoded by D_H_3–41 or D_H_3–3 using the same reading frame as PG9; and (3) the start position of the D gene occurred approximately in the middle of the HCDR3, at least 15 aa from the beginning and 8 aa from the end, based on the position in PG9 (fig. S1F). Such precursors were detected in 14 of 14 humans but only 6 of 60 RMs, and the median frequency among responders was 6.5-fold lower in RMs (median among responders, 4.8 per million) than in humans (median 31.6 per million) (fig. S1H). These results suggested that eliciting PG9-like responses in RMs could be substantially more challenging than in humans.

In summary, we identified two sets of Apex bnAb-related precursors in RMs with precursor frequencies comparable to those in humans. These analyses suggested that RMs are relevant preclinical models for evaluating ApexGT priming immunogens.

### ApexGT6 elicited serum binding antibody responses to the Apex epitope

**Fig. 2.**
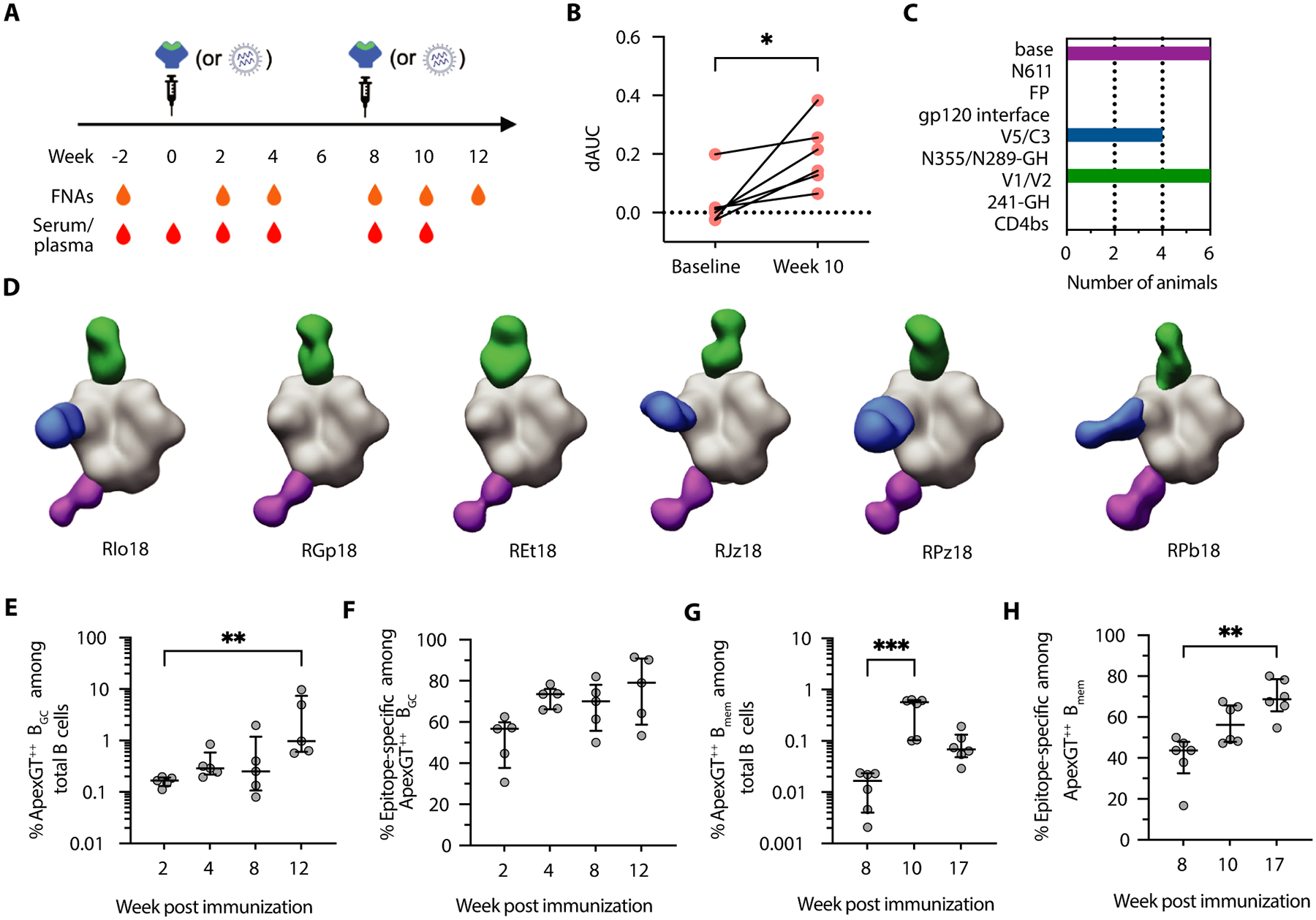
ApexGT6 adjuvanted-protein immunizations elicited strong Apex epitope-specific responses. **(A)** Experimental schematic of ApexGT6 bolus priming in RMs. The trimer cartoon represents protein immunogen (n=6), and the cartoon of nucleotide surrounded with LNP represents mRNA immunogen (n=6). **(B)** ELISA quantification of ApexGT6 epitope-specific binding serum IgG titers from experimental RMs (n=6) prior to immunization (baseline) and at 10 weeks after first immunization (week 10). dAUC was calculated by AUC (area under the curve) of ApexGT6-binding subtracted by AUC of ApexGT6.KO-binding. Wilcoxon matched-pairs signed rank test, n = 6. *P < 0.05. **(C)** Summary of week 10 polyclonal plasma IgGs binding to ApexGT6 congly soluble protein trimer detected by nsEMPEM (n=6). **(D)** Composite figures from nsEMPEM analysis of polyclonal responses at week 10, with animal IDs displayed below each figure. Antibodies are colored according to their targeted epitope clusters, as shown in Fig. 2C (i.e., V1/V2 binders are green, base binders are purple, V5/C3 binders are blue); ApexGT6 congly antigen is represented in gray. **(E)** ApexGT6^++^ B_GC_ cells as a percentage of total B cells. Gated on CD20^+^ CD38^−^ cells. Median and interquartile range are plotted depending on the scale in all figures unless otherwise stated. Dunn’s multiple comparisons test. **P < 0.01. **(F)** Epitope-specific B_GC_ cells as a percentage of ApexGT6^++^ B_GC_ cells. Median and interquartile range are plotted depending on the scale in all figures unless otherwise stated. Dunn’s multiple comparisons test. **(G)** ApexGT6^++^ B_mem_ cells as a percentage of total B cells. Gated on CD20^+^IgD^−^ cells. Median and interquartile range are plotted depending on the scale in all figures unless otherwise stated. Dunn’s multiple comparisons test. ***P < 0.001. **(H)** Epitope-specific B_mem_ cells as a percentage of ApexGT6^++^ B_mem_ cells. Median and interquartile range are plotted depending on the scale in all figures unless otherwise stated. Dunn’s multiple comparisons test. **P < 0.01. Data points represent biological replicate. Source data can be found in Data file S4.

We immunized RMs (N=6) bilaterally and subcutaneously in the deltoid, with 50 μg of soluble ApexGT6 congly protein and 375 μg of saponin/MPLA nanoparticle (SMNP) adjuvant (42) administered per animal flank. Bolus immunizations were conducted on day 0 and week 8. Fine-needle aspiration (FNA) (43) of axillary lymph nodes (LNs) and blood samples were collected at multiple time points for further investigation (Fig. 2A).

After the second immunization, all 6 animals produced serum IgG binding to the Apex epitope of ApexGT6 (Fig. 2B and fig. S2, A and B). Negative-stain electron microscopy polyclonal epitope mapping (nsEMPEM) demonstrated V1/V2-directed antibody responses present in all ApexGT6-immunized animals at week 10 (Fig. 2, C and D). In contrast, RMs immunized intramuscularly with the soluble native-like Env trimer BG505 MD39 (12) based on the same HIV isolate as ApexGT6 but lacking germline targeting mutations did not produce an Apex epitope-specific response detectable by nsEMPEM (44). Thus, the ApexGT design was necessary and sufficient to effectively trigger Apex epitope-specific serum IgG responses.

### ApexGT6 induced Apex epitope-specific GC and memory B cell responses.

Successful priming by a germline-targeting immunogen must produce a strong germinal center (GC) and/or memory B cell response derived from bnAb-precursor B cells. To investigate whether ApexGT6 could indeed prime bnAb-precursor B cells, we performed B cell sorting and receptor sequencing on ApexGT6-binding B cells from post-immunization LN and PBMC samples. LN B cells (CD20^+^CD38^−^) were mostly GC B cells (B_GC_) and will be referred to as such. In PBMCs, memory B cells (B_mem_) were defined as all CD20^+^IgD^−^ B cells, a strategy that encompassed both the more typical (IgG^+^CD27^+^) and the less conventional (CD27^−^ or IgM^+^) antigen-experienced B cell populations. Specificity for ApexGT6 was determined using ApexGT6 probes conjugated to two different fluorophores (ApexGT^++^) (fig. S2, C–D, and fig. S3). Specificity for the Apex epitope was determined by differential binding to ApexGT6 and ApexGT6.KO, a variant of ApexGT6 with two mutations in the Apex region (R169E and K171E in HxB2 numbering) that essentially abrogates binding by long-HCDR3 Apex bnAbs and related precursors (17). Apex epitope-specific B cells were defined as ApexGT6^++^ApexGT6.KO^−^.

We first measured the B_GC_ and B_mem_ cellular dynamics by flow cytometry. ApexGT6^++^ B_GC_ cells increased after immunization, with median frequency rising 5.8-fold from week 2 to week 12 (Fig. 2E). The fraction of Apex epitope-specific B_GC_ cells among ApexGT6^++^ B_GC_ cells was consistently high over that interval, with median values of 57% to 79% (Fig. 2F). In PBMCs, the frequencies of ApexGT6^++^ B_mem_ cells increased after the second immunization, with median values rising approximately 34-fold from 0.017% at week 8 to 0.572% at week 10, and then declining approximately 8-fold to 0.069% at week 17 (Fig. 2G). However, the fraction of Apex epitope-specific B_mem_ cells among ApexGT6^++^ B_mem_ cells increased continuously during that period, with the median value reaching approximately 69% by week 17 (Fig. 2H). Overall, two bolus priming immunizations of soluble ApexGT6 trimer immunogen with SMNP adjuvant elicited Apex epitope-specific B_GC_ and B_mem_ cell responses.

### Apex bnAb-like precursor responses were detected in all protein-immunized animals

To investigate whether the Apex epitope-specific B cell receptors (BCRs) were related to known Apex bnAbs, we sequenced the heavy and light chain VDJ genes of sorted B_GC_ cells and B_mem_ cells. After alignment of VDJ sequences to a previously described RM reference database (45, 46), we obtained 8,168 ApexGT6-negative and 2,376 ApexGT6^++^ paired (heavy and light chain) BCR sequences from 20 FNA samples and 678 ApexGT6^++^ BCR sequences from 9 PBMC samples in total (fig. S4A, Tables 1 and 2, Data files S1 and S2).

**Fig. 3.**
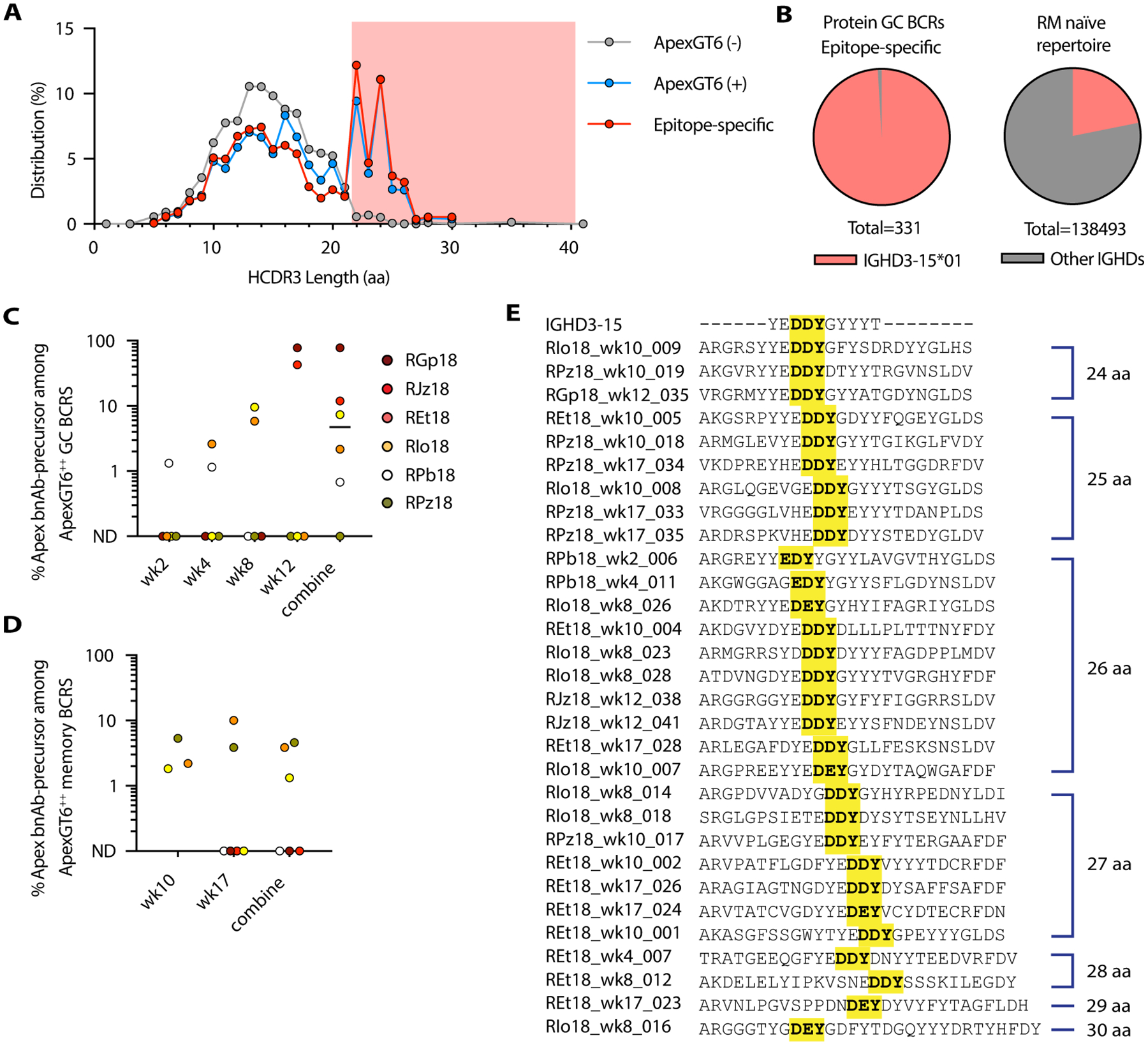
ApexGT6 adjuvanted-protein immunizations induced Apex bnAb-like precursor responses in all animals. **(A)** HCDR3 aa length distribution of BCRs in the lymph node. Lengths ≥ 22 aa are highlighted with a red shadow. **(B)** Frequency comparison of RM IGHD3–15 (represented in red) among two BCR datasets. Left: epitope-specific GC B cells with long HCDR3s (≥ 24 amino acids) sorted and sequenced from ApexGT6 soluble protein immunized RMs. Right: BCRs with long HCDR3s (≥ 24 amino acids) from a RM naïve BCR dataset (25). **(C)** The percentages of Apex bnAb-like precursor BCRs among ApexGT6^++^ GC BCRs increased over time. Each point represents the percentage from a specific RM, colored according to the legend. Medians are indicated by horizontal lines. **(D)** The percentages of Apex bnAb-like precursor BCRs among ApexGT6^++^ memory BCRs increased over time. Each point represents the percentage from a specific RM, colored according to the legend in Fig. 3C. Medians are indicated by horizontal lines. **(E)** Alignment of IGHD3–15 and representative HCDR3 of distinct Apex bnAb-like precursor lineages from the ApexGT6 adjuvanted-protein group. Sequences are aligned based on the HCDR3 length (labeled on the right) and the position of the DDY motif (highlighted in yellow). The sequence names include the animal IDs and timepoints of isolation. Source data can be found in Data file S4.

Among GC BCRs, we observed an enrichment of long HCDR3s with length ≥ 22 aa (aa) in ApexGT6^++^ and Apex epitope-specific BCRs but not in the BCRs that did not bind to ApexGT6 (Fig. 3A and fig. S4B). We focused our analysis on those BCRs with HCDR3 lengths equal to or longer than 24 aa, as nearly all known Apex bnAbs use a minimum length of 24 aa to penetrate the glycan shield (18, 37). We identified 331 Apex epitope-specific paired BCRs with long HCDR3s (≥ 24 aa) in five out of six animals (Table 1). VDJ alignment analysis revealed that ApexGT6^++^ and Apex epitope-specific BCRs with long HCDR3s were nearly entirely (99%) composed of sequences utilizing IGHD3–15 (Fig. 3B and fig. S4C). In contrast, in a control dataset of naïve BCR sequences from 60 RMs (25), less than 22% of BCRs with long HCDR3s used IGHD3–15 (Fig. 3B). In addition, none of the Apex epitope-specific GC BCRs met the D_H_3–41 precursor criteria described above. However, we observed that more than 94% of Apex epitope-specific GC BCRs with long HCDR3s elicited by ApexGT6 immunization met the Apex bnAb-like precursor criteria described earlier (fig. S1E, “RM DDY”), suggesting that the majority of these sequences were Apex bnAb-like precursors (Table 1).

To further investigate the development of Apex bnAb-like precursor B cell responses after immunization, we analyzed their frequencies among ApexGT6^++^ GC BCRs at different sampling time points. The percentages of Apex bnAb-like precursors among ApexGT6^++^ GC BCRs increased over time (Fig. 3C). Overall, we identified Apex bnAb-like precursor GC BCRs in 5 of 6 RMs, with a median frequency of 5%.

In PBMC samples for three of the six animals, we identified 21 Apex bnAb-like precursors at weeks 10 and 17 (Table 1, Fig. 3D). In one of these animals, RPz18, we detected six different Apex bnAb-like precursor lineages in PBMCs but none in FNA samples (Fig. 3, C and D), which indicated that the Apex bnAb-like precursor responses elicited by ApexGT6 may be underestimated in some animals due to undersampling. Due to sample health technical issues, we recovered insufficient BCR sequences from PBMCs of the other 3 animals to assess the potential presence of Apex bnAb-like precursors. Nevertheless, we did observe multiple Apex bnAb-like precursors in the LNs of three animals (Fig. 3C). In the three animals with detectable Apex bnAb-like precursors in PBMCs, the median frequency of Apex bnAb-like precursors among ApexGT6^++^ memory BCRs was 3.8% (Fig. 3D).

Overall, we detected 333 paired Apex bnAb-like precursor BCRs from Apex epitope-specific B_GC_ cells and B_mem_ cells in response to 1 or 2 immunizations with ApexGT6. These 333 sequences could be clustered into 30 clonal lineages (Fig. 3E). These lineages exhibited different HCDR3 lengths, with distinct junctional sequences within the HCDR3s, and therefore most likely corresponded to distinct clones derived from specific precursors.

### Immunization with mRNA encoding membrane-bound ApexGT6 induced Apex epitope-specific serum binding antibodies and Apex bnAb-related memory B cell responses

To test if mRNA immunization strategies could be used to prime Apex epitope-specific responses, we also immunized a group of six RMs with 50 μg of mRNA-LNP encoding cleavage-independent membrane-bound ApexGT6 (ApexGT6 L14 gp151), bilaterally and intramuscularly (IM) in the deltoid. The experimental timeline and sample collections were identical to the adjuvanted-protein group (Fig. 2A).

**Fig. 4.**
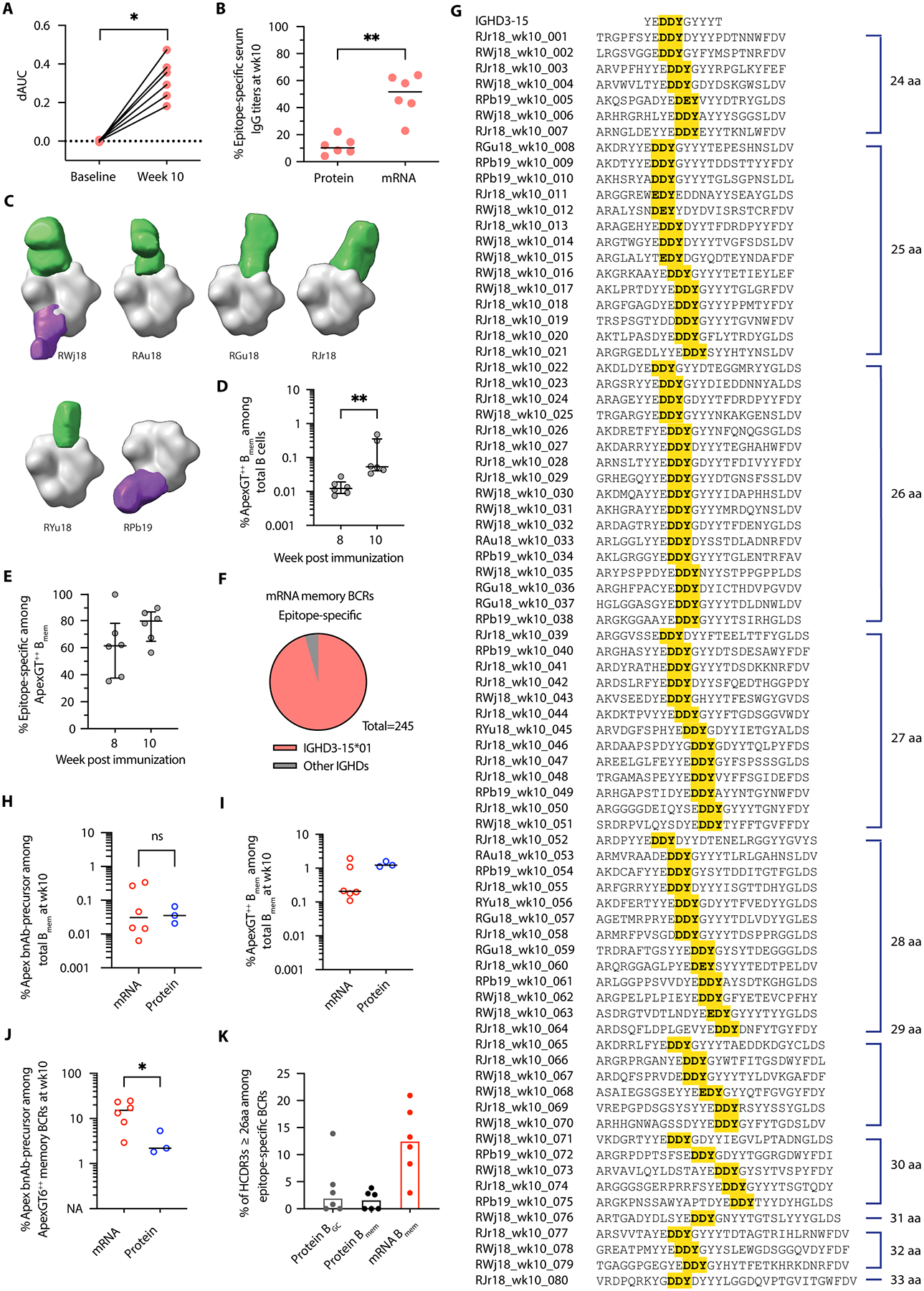
membrane-bound ApexGT6 mRNA-LNP induced robust Apex bnAb-like precursor memory responses in all immunized animals. **(A)** ELISA quantification of ApexGT6 epitope-specific binding serum IgG titers from experimental RMs (n=6) prior to immunization (baseline) and at 10 weeks after first immunization (week 10). dAUC was calculated by AUC of ApexGT6-binding subtracted by AUC of ApexGT6.KO-binding. Wilcoxon matched-pairs signed rank test, n = 6. *P < 0.05. **(B)** ELISA quantification of the percentages of ApexGT6-binding serum IgG that are epitope-specific at week 10 of protein and mRNA groups. Medians are indicated by horizontal lines. Mann-Whitney test, compare ranks. **P < 0.01. **(C)** Composite figures from nsEMPEM analysis of polyclonal responses at week 10, with animal IDs displayed below each figure. Antibodies are colored according to their targeted epitope clusters, as shown in Fig. 2C; ApexGT6–5CC antigen is represented in gray. **(D)** ApexGT6^++^ B_mem_ cells as a percentage of total B cells. Gated on CD20^+^IgD^−^ cells. Median and interquartile range are plotted depending on the scale in all figures unless otherwise stated. Dunn’s multiple comparisons test. **P < 0.01. **(E)** Epitope-specific B_mem_ cells as a percentage of ApexGT6^++^ B_mem_ cells. **(F)** Frequency of RM IGHD3–15 (represented in red) among epitope-specific memory BCRs with long HCDR3s (≥ 24 amino acids) sorted and sequenced from ApexGT6 membrane-bound mRNA immunized RMs. **(G)** Alignment of IGHD3–15 and representative HCDR3 of distinct Apex bnAb-like precursor lineages from the ApexGT6 mRNA-LNP group. Sequences are aligned based on the HCDR3 length (labeled on the right) and the position of the DDY motif (highlighted in yellow). The sequence names include the animal IDs and timepoints of isolation. **(H)** Comparison of the percentages of Apex bnAb-like precursor BCRs among total B_mem_ cells at week 10 between mRNA and protein immunizations. Medians are indicated by horizontal lines. Mann-Whitney compare ranks test. ns > 0.05. **(I)** Comparison of the percentages of ApexGT6^++^ B_mem_ among total B_mem_ cells at week 10 between mRNA and protein immunizations. Medians are indicated by horizontal lines. **(J)** Comparison of the percentages of Apex bnAb-like precursor BCRs among ApexGT6^++^ BCRs at week 10 between mRNA and protein immunizations. Medians are indicated by horizontal lines. Mann-Whitney compare ranks test. *P < 0.05. **(K)** Percentages of BCRs with very long HCDR3s (≥ 26 aa) among epitope-specific BCRs from each immunized macaque. Medians are plotted as bars. Data points represent biological replicate. Source data can be found in Data file S4.

After two immunizations with mRNA-LNP, all six animals produced serum IgG that bound to the Apex epitope (Fig. 4A). Approximately 50% of the ApexGT6-specific response was directed to the Apex epitope (fig. S5A). In a control experiment first described elsewhere (44), RMs were immunized with mRNA encoding a native-like Env trimer (BG505 MD39.3) based on the same HIV isolate as ApexGT6 and also designed as a cleavage-independent membrane-bound trimer. We report here that BG505 MD39.3 mRNA induced an Apex epitope-specific serum IgG response that was weaker than the ApexGT6 mRNA response (fig. S5, A and B). When comparing serum IgG titers between the ApexGT6 protein and mRNA groups, we found that protein immunization resulted in higher titers to the immunogen (fig. S5C). However, the magnitude of the Apex epitope-specific response (fig. S5D) and the percentage of serum IgG binding antibodies that were Apex epitope-specific (Fig. 4B) were both higher in the membrane-bound mRNA group. This was true even though the soluble protein had two glycan holes filled, whereas the mRNA immunogen did not, suggesting that the mRNA-delivered membrane-bound platform may be more effective than adjuvanted soluble protein in eliciting an Apex epitope-directed response.

Although the ELISA showed a strong serum response to the Apex epitope in the mRNA group, negative stain electron microscopy polyclonal epitope mapping (nsEMPEM) analyses revealed that, after mixing serum-derived polyclonal Fabs from the mRNA immunization group with soluble ApexGT6 trimer, the Fab-Env complexes were predominantly monomeric. As a result, we were unable to obtain enough trimer-liganded classes to reconstruct 3D volumes. The phenomenon of antibody-driven trimer disassembly has been observed previously (44, 47, 48). A similar phenomenon was observed in RMs immunized with mRNA encoding membrane-bound native-like MD39 Env (44), suggesting that the antibodies in the post membrane-bound trimer immunized plasma induced degradation of soluble trimers during sample preparation of nsEMPEM.

To prevent trimer disassembly during plasma incubation in nsEMPEM, we created a variant of ApexGT6 (ApexGT6–5CC) with two additional disulfide bonds, 49C-555C (intra-protomer) and 73C-561C (inter-protomer). This strategy had worked previously for BG505 MD39.3 (44, 48). We found that these modifications reduced the fidelity of assembly of the ApexGT6 trimer, in that nsEMPEM in the absence of plasma revealed a mixture of trimeric and non-trimeric species, consistent with our Size Exclusion Chromatography coupled with Multi-Angle Light Scattering (SEC-MALS) data during purification. However, nsEM with ApexGT6–5CC in the presence of plasma revealed that the trimeric species that did assemble (and presumably formed the inter-protomer disulfide) was protected from degradation. No significant additional disassembly occurred after adding the plasma sample, and nsEMPEM was able to characterize the epitopes targeted on the trimer. This approach allowed us to obtain enough trimer-liganded classes to reconstruct 3D volumes and observe V1/V2-directed antibody responses in 5 of 6 ApexGT6 mRNA-immunized animals at week 10 (Fig. 4C). Although our nsEMPEM analysis indicated that one animal lacked Apex serological responses, subsequent analyses of memory BCRs including SPR measurements revealed Apex-specific B_mem_ in this animal (Data file S3). Overall, use of ApexGT6–5CC allowed nsEMPEM detection of Apex-specific serum IgG responses in a majority of ApexGT6 mRNA-immunized animals.

Our nsEMPEM analysis also revealed that the mRNA group had fewer animals with detectable base-binding antibodies (2/6) (Fig. 4C) compared to the protein group (6/6) (Fig. 2D). This difference was likely due to the structural characteristics of the immunogens. The soluble protein was truncated at D664 and exposed a large non-glycosylated surface at the base, whereas the membrane-anchored form likely made the base substantially less accessible to antibody.

We next measured the germinal center and memory cellular dynamics following ApexGT6 mRNA-LNP immunization via flow cytometry. In PBMCs, the frequencies of ApexGT6^++^ B_mem_ cells increased after the second immunization (Fig. 4D). The fraction of Apex epitope-specific B_mem_ cells among ApexGT6^++^ B_mem_ cells remained consistently high during that period, with median values ranging from 60% to 80% (Fig. 4E). In contrast, we observed minimal ApexGT6-specific cell responses in LN samples (fig. S5E). Given the robust B_mem_ response seen in the blood, we concluded that IM mRNA-LNP immunizations predominantly drained to an inaccessible LN, limiting the information obtained from the axillary LN FNAs. Nevertheless, we demonstrated that two immunizations of mRNA encoding membrane-bound ApexGT6 induced strong Apex epitope-specific B_mem_ cell responses.

We obtained 1,401 paired heavy and light chain BCR sequences from the PBMCs of the 6 animals immunized with mRNA-LNPs (Table 3 and Data file S2). We observed an enrichment of long HCDR3s (≥ 24 aa) in Apex epitope-specific BCRs (fig. S5, F and G). These Apex epitope-specific BCRs with long HCDR3s primarily contained IGHD3–15 (96%), and more than 88% were Apex bnAb-like according to our definition (Fig. 4F and Table 3). As for the protein group, none of the Apex epitope-specific BCRs met the criteria for the D_H_3–41 precursor. In total, we identified 218 paired Apex bnAb-like precursor memory BCRs from Apex epitope-specific B_mem_ cells clustered into 80 distinct lineages across all six animals (Fig. 4G). These lineages exhibited high polyclonal diversity. A substantial number of clones possessed very long HCDR3 (length ≥ 26 aa). Among all six animals, the median frequency of Apex bnAb-like precursors among B_mem_ cells at week 10 was 0.0308% (approximately 1 in 3252), similar to that of the protein immunization group (Fig. 4H).

When comparing the protein and mRNA-LNP groups further, we observed a higher frequency of ApexGT6^++^ B_mem_ cells among the total B_mem_ cells in the protein group (Fig. 4I). However, among ApexGT6^++^ memory BCRs, the mRNA-LNP group showed a higher percentage of Apex bnAb-like precursors compared to the protein group (Fig. 4J). Hence, under the dosing conditions of our experiments, adjuvanted-protein immunization induced a stronger ApexGT6^++^ B_mem_ response, whereas the mRNA-encoded membrane-bound platform induced an ApexGT6^++^ B_mem_ response with superior focusing on the Apex epitope, which was consistent with the serum results. The balance of these factors resulted in protein and mRNA-LNP inducing similar overall frequencies of Apex bnAb-like precursors among B_mem_ cells.

The mRNA-LNP immunized animals produced a higher frequency of Apex bnAb-like precursors with very long HCDR3s (length ≥ 26 aa) compared to RMs immunized with protein (Fig. 4K and fig. S5H). This suggested that mRNA-encoded membrane-bound ApexGT6 might have greater potential to recruit B cells with very long HCDR3s. Longer HCDR3s are associated with higher breadth and potency for human Apex bnAbs (fig S5, I and J), hence any advantage in priming bnAb-like precursors with very long HCDR3s could potentially improve vaccine outcomes from future germline-targeting sequential vaccination regimens including more native-like boosters. In summary, two bolus immunizations of mRNA-LNP encoding membrane-bound ApexGT6 induced strong Apex epitope-specific B_mem_ cell responses in all six animals, with a higher proportion of Apex bnAb-like precursors compared to adjuvanted-protein immunization.

### Apex bnAb-like precursor antibodies gained somatic hypermutation over time and acquired affinity to less mutated Apex epitopes

**Fig. 5.**
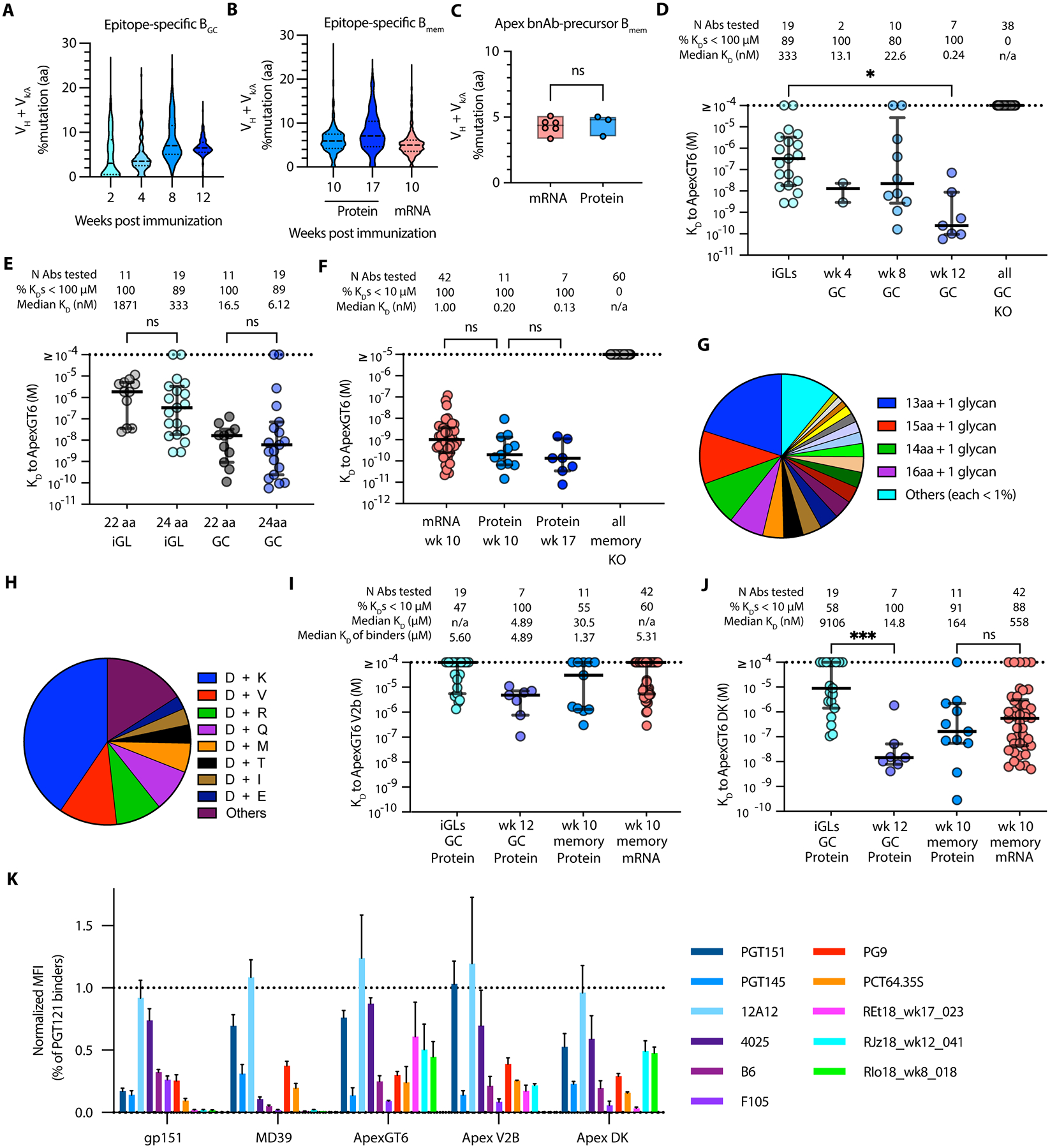
Induced Apex bnAb-like precursor antibodies gained somatic hypermutation over time and acquired enhanced affinity for native-like Apex. **(A)** Somatic hypermutation (SHM) of Apex epitope-specific GC BCRs at sampled timepoints. SHM is calculated by combining the number of mutations on V_H_ and V_κ/λ_, then dividing by their combined length. All calculations are based on aa. Thick lines indicate median values, and dash lines indicate 25 and 75% quantiles. **(B)** SHM of Apex epitope-specific memory BCRs at sampled timepoints. Calculated in the same manner as panel 5A. **(C)** Comparison of the median values of SHM of Apex bnAb-like precursor memory BCRs from each animal at week 10 between the mRNA and protein groups. Floating bars indicate minimum and maximum, with lines indicating median values. Mann-Whitney compare ranks test. ns > 0.05. **(D)** SPR affinity measurement of mAbs derived from representative Apex bnAb-like precursor GC BCRs isolated at different timepoints and their inferred germlines (iGLs) binding to ApexGT6 and ApexGT6.KO. Lines indicate median values and 25 and 75% quantiles. Dunn’s multiple comparisons test. *P < 0.05. **(E)** SPR affinity measurement of mAbs derived from representative Apex bnAb-like precursor GC BCRs with HCDR3 lengths of 22 to 23 aa (labeled as 22 aa GC) or 24 aa and longer (labeled as 24 aa GC), as well as their inferred germlines (labeled as 22 aa iGL and 24 aa iGL, respectively) binding to ApexGT6. Lines indicate median values and 25 and 75% quantiles. Dunn’s multiple comparisons test. ns > 0.05. **(F)** SPR affinity measurement of mAbs derived from representative Apex bnAb-like precursor memory BCRs isolated from mRNA or protein group at week 10 or week 17 binding to ApexGT6 and ApexGT6.KO. Lines indicate median values and 25 and 75% quantiles. Dunn’s multiple comparisons test. ns > 0.05. **(G)** Frequency of different V2b loop features on Envs across HIV strains from the Los Alamos database. **(H)** Frequency of different aa combinations at position 167 and 169 on Envs across HIV strains from the Los Alamos database. **(I)** SPR affinity measurement of Apex bnAb-like precursor mAbs (iGLs and week 12 GC BCRs from protein group, week 10 memory BCRs from protein and mRNA-LNP groups) binding to ApexGT6-V2b. Lines indicate median values and 25 and 75% quantiles. **(J)** SPR affinity measurement of Apex bnAb-like precursor mAbs (iGLs and week 12 GC BCRs from protein group, week 10 memory BCRs from protein and mRNA-LNP groups) binding to ApexGT6-DK. Lines indicate median values and 25 and 75% quantiles. Dunn’s multiple comparisons test. ns > 0.05, ***P < 0.001. **(K)** Cell surface antigenic profile assay for membrane-bound ApexGT6 and its DK and V2b loop variants. DNA-expressed membrane-anchored trimers binding to selected IgGs. Mean-fluorescence intensity (MFI) via fluorescence-activated cell sorting (FACS) binding was normalized to PGT121 binding with error bars representing standard deviation (n = 2). Data points represent technical replicate. All trimers are based on the same design in ref (18). Apex trimers contain mutations described in the text. Source data can be found in Data file S4.

Our findings demonstrated that ApexGT6, whether administered as adjuvanted-protein or mRNA-LNP, can effectively prime Apex bnAb-like precursors. We next wanted to determine if these cells can effectively acquire mutations and increase their affinity to native Env over time (Data file S3). Sequence analysis revealed that the somatic hypermutation (SHM) of the epitope-specific GC BCRs increased over time following protein immunization, reaching median values of 6.5% aa mutation after the second immunization (Fig. 5A and fig. S6A). SHM for Apex bnAb-like precursor GC BCRs also increased over time (fig. S6B). We also observed a slight increase in SHM among epitope-specific memory BCRs from week 10 to week 17 in the protein-immunized group (Fig. 5B and fig. S6C). Due to low cell numbers, we were only able to collect B**_mem_** cell sequences at week 10 for the mRNA group after the second immunization, hence longitudinal SHM analysis for that group after the boost was not possible. For epitope-specific memory BCRs, the degree of SHM in the mRNA group was similar to but slightly lower than in the protein group at week 10 (Fig. 5B and fig. S6C). For Apex bnAb-like precursor memory BCRs at week 10, the median values of SHM from mRNA-LNP immunized animals were similar to those of the protein group (Fig. 5C). These data indicated that ApexGT6 immunization by protein or mRNA induced substantial SHM.

To investigate whether Apex bnAb-like precursor GC BCRs primed by protein immunization accumulated affinity-enhancing mutations, we produced monoclonal antibodies (mAbs) of selected representative Apex bnAb-like precursors from multiple lineages at various time points, isolated from different animals. We also produced inferred germline versions (iGL) of each antibody with all recognizable templated SHMs reverted to germline. We then evaluated the binding affinities of these mAbs to ApexGT6 using SPR. Apex bnAb-like precursor mAbs derived from the GC BCRs showed improved affinity by week 12 (Fig. 5D). At week 12, the median K_D_ of mAb binders was around 0.24 nM, an increase of more than 258-fold compared to the corresponding inferred germline versions (fig. S6D). All tested ApexGT6 binders lacked detectable affinity for ApexGT6.KO, indicating that they were Apex epitope-specific (Fig. 5D). Those that did not bind to ApexGT6 did not bind to ApexGT6.KO either. Hence, in those cases, although ApexGT6 binding was detected by the original B cell sorting using substantial avidity, the binding was weak enough that it could not be detected by SPR at the highest trimer concentration tested (approximately 10 μM). It remains possible that at least a fraction of these cells were not true ApexGT6 binders but were sorted due to a wide gating strategy.

Although we used strict minimum length criteria for defining an Apex bnAb-like HCDR3, based on the known Apex bnAbs, it remains uncertain whether those with HCDR3s longer than 20 aa but shorter than 24 aa have the potential to become Apex bnAbs. A considerable percentage of Apex epitope-specific binders after ApexGT6 immunizations had HCDR3 lengths of 22 to 23 aa. This suggested that a 22- or 23-aa HCDR3 was at least long enough to penetrate the N156/N160 glycan shield and bind to the Apex region of ApexGT6. Furthermore, there were no statistically significant differences in affinity toward the immunogen among these mAbs (Fig. 5E). Follow-up studies will be required to understand the potential of Abs with length 22 – 23 aa HCDR3s for bnAb development.

We next evaluated affinities of memory BCRs induced by protein and mRNA vaccination. For B_mem_ cells, all 60 mAbs identified to be Apex bnAb-like precursors by sequence analysis were Apex epitope-specific binders (Fig. 5F). The affinity for ApexGT6 at week 10 was similarly strong for mAbs from both the mRNA and protein groups, with median K_D_ values of 1 nM and 0.2 nM, respectively. The affinities observed at week 17 were similar to those observed at week 10 in the protein group for which we had mAbs at both timepoints. Together, these SPR results suggested that the majority of GC and memory-derived Apex bnAb-like precursor BCRs identified by our sequence criteria were directed towards the apex region of ApexGT6, with affinity-enhancing SHM acquired through immunization.

We also evaluated whether post-prime Apex bnAb-like precursor BCRs could bind to an apex epitope closer to native, to assess whether affinity maturation against the ApexGT6 immunogen had resulted in on-track maturation toward bnAb development. The Apex epitope on the trimer is composed of two main motifs, strand C (49, 50) and V2b loop (18, 51), both of which play a crucial role in antigen-antibody recognition. To improve the affinity to the Apex bnAb-like precursors, these motifs were modified with multiple germline-targeting mutations in ApexGT6. However, an Apex bnAb should be able to recognize an epitope that has these germline-targeting mutations reverted to the ones most commonly found in HIV strains in global circulation. For the V2b loop (179–191, based on HXB2 sequence numbering), analysis of Env sequences from the Los Alamos database (52) indicated that the most common features include a hydrophilic loop of length 13 to 16 aa with one or two glycosylation sites (Fig. 5G and fig. S6E). The V2b loop of ApexGT6 fulfills the length requirement (13 aa), but includes multiple hydrophobic GT mutations, and lacks glycosylation sites. For strand C, two germline-targeting mutations, D167N and K169R, were found to have favorable interactions with the germline-reverted variant of PCT64 (18). Sequence analysis indicated that 167D and 169K are the most frequent aa at these two positions among HIV isolates (Fig. 5H and fig. S6F). Accordingly, we assessed the affinities of post-primed Apex bnAb-like mAbs and their iGLs for variants of the ApexGT6 trimer with either a wild type V2b loop (derived from isolate 191084, referred to as ApexGT6-V2b), or with 167D and 169K (referred to as ApexGT6-DK) on the strand C.

When testing the post-immunized Apex bnAb-like mAbs against ApexGT6-V2b, the affinity decreased substantially, with only 47% of the iGLs having detectable binding and with affinities weaker than 1 μM (Fig. 5I). This decrease in affinity appeared to be caused by reverting the hydrophobic mutations to hydrophilic residues (L187N and W188S), and by adding a glycosylation site at N187 (fig. S6G). After the second immunization, all tested Apex bnAb-like mAbs derived from GC BCRs were able to bind to ApexGT6-V2b, with a median K_D_ value of approximately 4.9 μM. More than half of the Apex bnAb-like mAbs derived from memory BCRs were capable of binding to ApexGT6-V2b for both the mRNA and protein groups at week 10. The median K_D_ values among binders were 1.4 μM and 5.3 μM, respectively.

Compared to ApexGT6, ApexGT6-DK abolished binding of nearly half of the clones that bound iGL, suggesting that these GT mutations are required for priming these precursors in RMs. The affinity of Apex bnAb-like GC-derived mAbs for the DK trimer increased over time (fig. S6H). After the second immunization, more than 85% of the tested Apex bnAb-like mAbs derived from GC BCRs showed an affinity stronger than 100 nM for the ApexGT6-DK trimer (Fig. 5J). For post-immunized Apex bnAb-like memory-derived mAbs, the affinity to the DK version of ApexGT6 was similarly strong for both the mRNA and protein groups, with median K_D_ values of 164 nM and 558 nM, respectively.

To evaluate neutralization of Apex bnAb-related precursor mAbs, we tested five such mAbs with highest affinity for ApexGT6 and variants against both wild-type (WT) strains and GT5/GT6 modified pseudoviruses (fig. S7). As expected after immunization with a germline-targeting priming alone, we observed no neutralization against pseudoviruses bearing WT Env. Most pseudoviruses with GT5 and GT6 mutations had insufficient titers for testing. From the few variants with sufficient titers, RJz18_wk12_038 showed modest neutralization specifically against a GT5-modified virus containing a partially WT strand C.

We also conducted a cell surface antigenic profile assay to assess the affinity of Apex bnAb-like binders against the membrane-bound ApexGT6 trimer and its DK and WT V2b loop variants. All three Apex trimers exhibited an overall antigenic profile similar to that of MD39, except for increased binding to the non-neutralizing V3-directed mAb 4025 (Fig. 5K). The three ApexGT6-induced Apex bnAb-like mAbs demonstrated strong binding to ApexGT6, but not to membrane-bound BG505 SOSIP (referred as gp151) or BG505 MD39 (referred as MD39) trimers. REt18_wk17_023 and RJz18_wk12_041 showed good binding to ApexGT6-V2b, while RIo18_wk8_018 and RJz18_wk12_041 showed good binding to ApexGT6-DK. Taken together, these findings suggested that two doses of ApexGT6 immunization induced Apex bnAb-like precursor B cells with SHMs that conferred the ability to recognize Apex epitopes closer to native on soluble protein and membrane-bound trimers, providing evidence for vaccine-induced maturation toward bnAb development.

### ApexGT6 induced mAbs possessed structural features similar to three prototype human Apex bnAbs.

To investigate how the Apex bnAb-like BCRs induced by ApexGT6 recognize the apex, we determined cryoEM structures of three high affinity binders complexed with ApexGT6: RJr18_wk10_017 (RM017) from the mRNA group, and RIo18_wk8_018 (RM018) and RJz18_wk12_038 (RM038) from the protein group (fig. S8 and S9, Table S2). We also determined crystal structures of unliganded RM018 and RM038, whereas RM017 did not crystallize (Table S3).

**Fig. 6.**
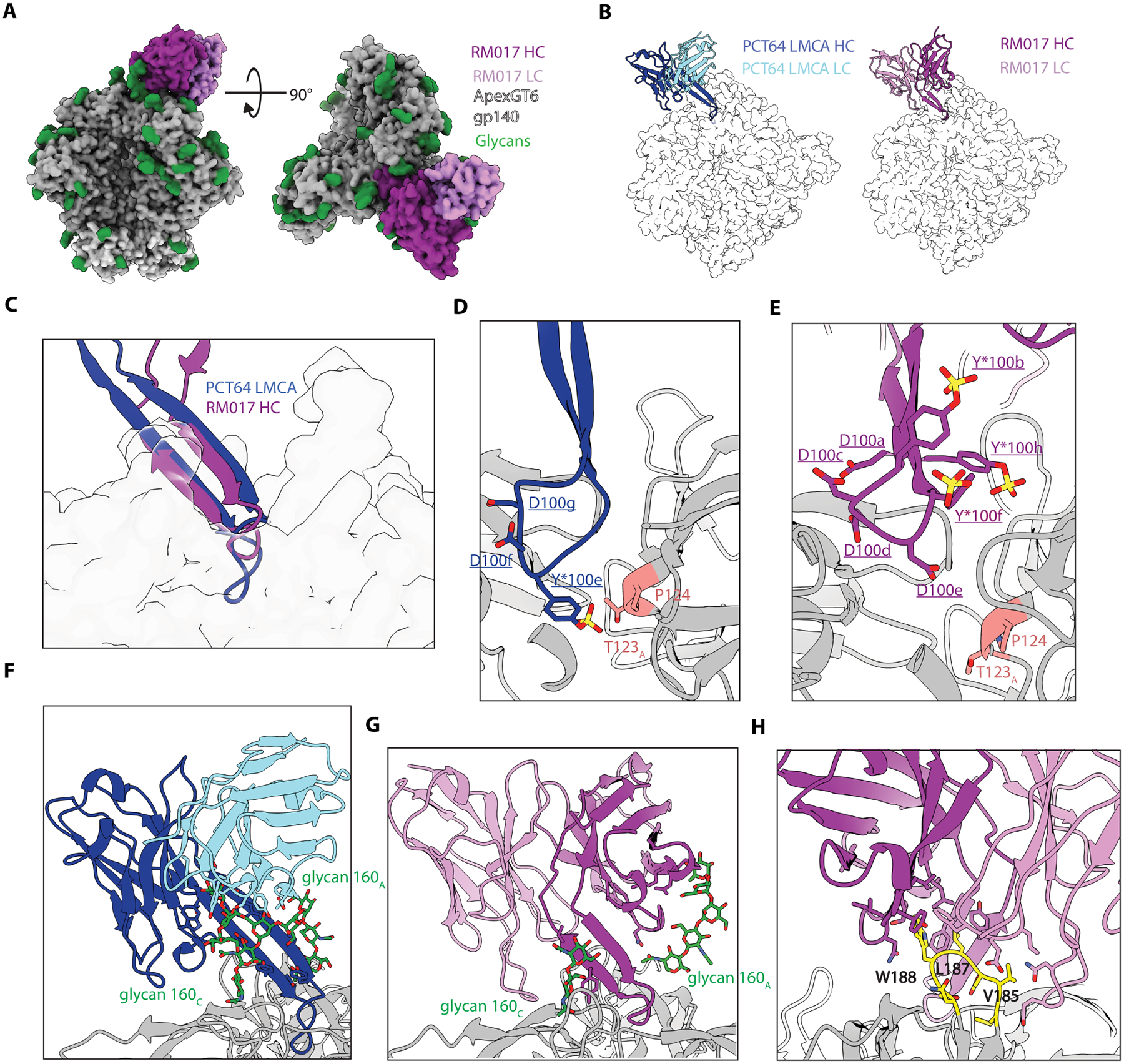
ApexGT6 induced mAbs possessed structural features similar to PCT64. **(A)** Top-down and side views of RM017 (purple) binding at the trimer 3-fold axis of ApexGT6 (grey). Glycans are colored as green spheres. **(B)** RM017 (right side: heavy chain: dark purple, light chain: light purple) employs an extended HCDR3 for binding at the trimer 3-fold axis of ApexGT6 (white surface), aligned to PCT64 LMCA (left side: heavy chain: blue, light chain: cyan) binding to ApexGT2.2MUT (white surface), but with flipped heavy and light chain. Two liganded structures were aligned using gp120_A_ of each ApexGT trimer. **(C)** Zoomed-in views of PCT64 LMCA (blue) and RM017 (fuchsia) HCDR3s interact to the center of Apex (white surface), respectively. **(D)** Zoomed-in views of the DDY motif of PCT64 LMCA (blue sticks) interacting to the 3-fold axis of ApexGT2.2MUT (grey cartoon). T123 and P124 at the 3-fold axis depicted as salmon sticks. **(E)** Zoomed-in views of the DDY motif of RM017 (purple sticks) interacting to the 3-fold axis of ApexGT6 (grey cartoon). T123 and P124 at the 3-fold axis depicted as salmon sticks. **(F)** Side view of the VL gene segment of PCT64.LMCA (cyan cartoon) interacting with the N160 glycan (green sticks) on the trimer apex of ApexGT2.2MUT (grey). **(G)** Side view of the VL gene segment of RM017 (light purple cartoon) interacting with the strand C of the trimer apex of ApexGT6 (grey cartoon). **(G)** Zoomed-in view shows the hydrophobic pocket (with residues displayed as sticks) formed by HCDR2, the heavy and light chain interface, and the 17-amino-acid LCDR1 of RM017 interacting with the engineered hydrophobic V2b loop (shown as a yellow cartoon, with hydrophobic mutations as sticks).

The RM017 liganded structure showed the expected 1:1 stoichiometry and a tilted angle of approach relative to the 3-fold axis, similar to other Apex bnAbs and their inferred germlines (18, 53) (Fig. 6A). From a top-down view, RM017 used its extended long HCDR3 to bind to the center of the ApexGT6 apex, similar to PCT64 LMCA binding to ApexGT2 (18), but with flipped heavy and light chains (Fig. 6B). The HCDR3 of RM017 approaches at a similar angle as the HCDR3 of PCT64 LMCA, with both forming beta hairpin conformations to penetrate the trimer apex, though HCDR3 of PCT64 LMCA penetrates deeper into the ectodomain surface (Fig. 6C). The LMCA HCDR3 tip extends further into the trimer 3-fold axis, with its sulfated tyrosine at position 100e reaching in to interact with proline at position 124 on gp120 (Fig. 6D). In RM017, the HCDR3 tip contains multiple negatively charged residues with the aspartic acid at 100e extending furthest into the trimer axis (Fig. 6E and fig. S10A). While the LMCA light chain has few peptidic interactions with the trimer, it helps the heavy chain stabilize N160C glycan contacts along the HCDR3 (Fig. 6F). In contrast, the flipped chain orientation of RM017 shows the light chain making peptidic interactions with outer-facing C-strand residues of protomer A, whereas HCDR2 and HFRW3 work together to stabilize the N160A glycans (Fig. 6G). Beyond its central Apex interactions through the protruding HCDR3, RM017 also interacts favorably with the engineered V2b loop of ApexGT6. The HCDR2, upper HCDR3 stem, LCDR1, and LCDR3 of RM017 form a hydrophobic pocket (Fig. 6H) that interacts with the engineered hydrophobic V2b loop of ApexGT6 (fig. S10, B and C). RM017 has a 17-amino-acid LCDR1, similar to DH650 (macaque CD4bs mAb, IGVk2) (38), which may represent a light chain feature in macaques. The PCT64 LMCA interaction with ApexGT2 showed similar features in its HCDR3 and HCDR1 (18).

**Fig. 7.**
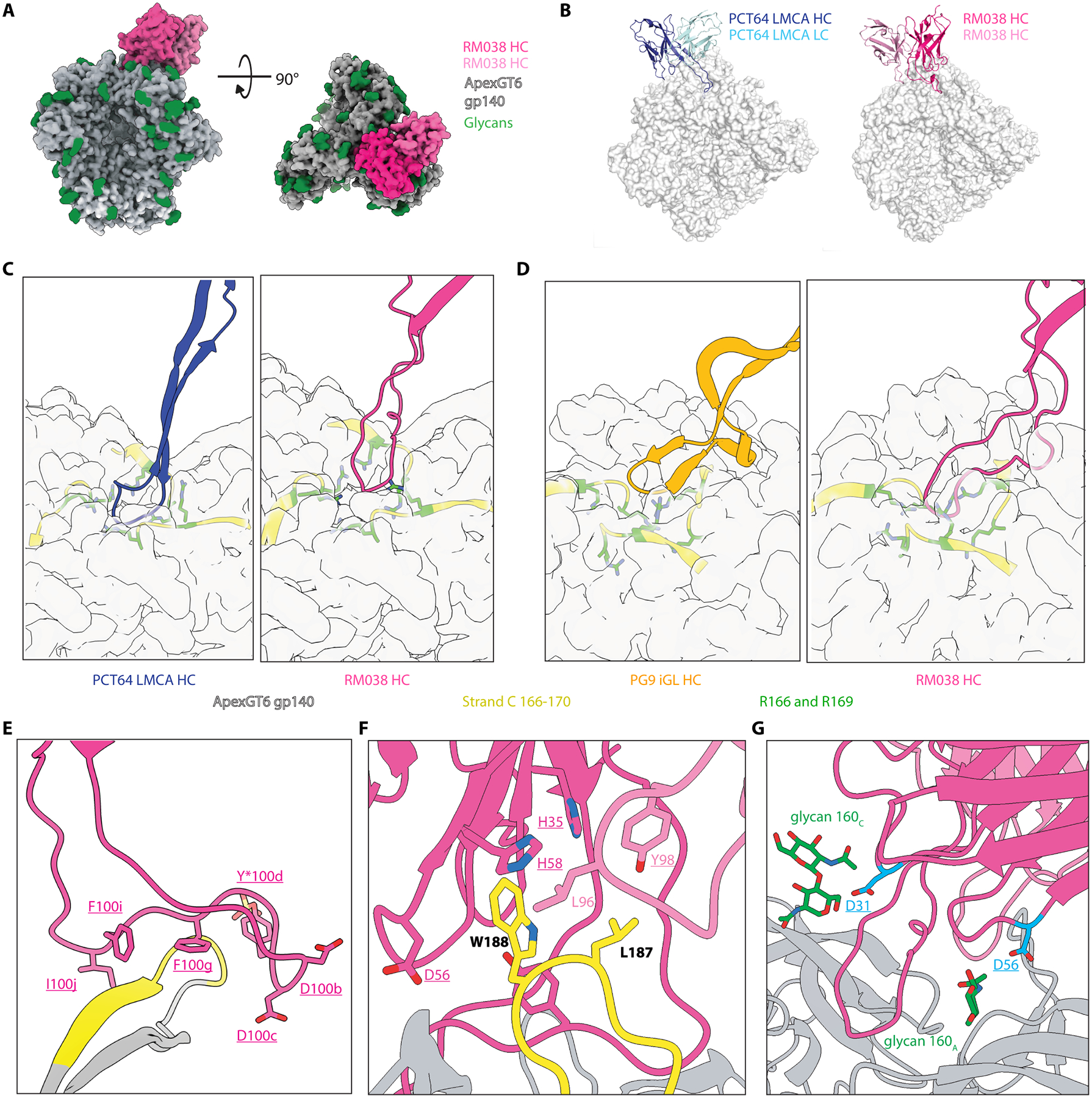
ApexGT6 induced mAbs exhibited hybrid structural features of PCT64 and PG9. **(A)** Top-down and side views of RM038 (pink) binding at the trimer 3-fold axis of ApexGT6 (grey). Glycans are colored as green spheres. **(B)** RM038 (right side; heavy chain: fuchsia, light chain: pink) employs an extended HCDR3 for binding at the trimer 3-fold axis of ApexGT6 (white surface), aligned to PCT64 LMCA (left side; heavy chain: blue, light chain: cyan) binding to ApexGT2 (white surface), but with flipped heavy and light chain. Two liganded structures were aligned using gp120_A_ of each ApexGT trimer. **(C)** Zoomed-in views of PCT64 LMCA (blue) and RM038 (fuchsia) HCDR3s interact to the center of Apex (white surface), respectively. 166–170 residues on the strand C colored as yellow with R166 and R169 shown as green sticks. **(D)** 90° rotation view of 6C showing the second lobe of RM038 (fuchsia) HCDR3 bind to strand C of ApexGT6, similar to PG9 iGL (orange) binding to ApexGT3. **(E)** Side view of the HCDR3 of RM038 and strand C (166–170 residues colored as yellow) on gp120_A_ of ApexGT6, illustrating the hybrid features of PCT64 and PG9, with key residues depicted as sticks. **(F)** Zoomed-in view of the hydrophobic pocket (residues showed as sticks) interactions with the engineered hydrophobic V2b loop (highlighted as yellow cartoon, with hydrophobic mutations depicted as sticks). **(G)** Acidic SHMs (cyan) on HCDR1 and HCDR2 may enhance binding to the N160 glycans (green).

The structure of RM038 in complex with ApexGT6 also showed a 1:1 stoichiometry and a tilted angle of approach relative to the 3-fold axis (Fig. 7A). From a top-down view, RM038 used its extended long HCDR3 to bind to the center of the apex of ApexGT6, similar to that of PCT64 LMCA to ApexGT2 (18), but with heavy and light chains flipped as well (Fig. 7, B and C). The aspartic acids within the DDY motif interacted with the residues around the top surface of the 3-fold axis (R166, N167, and R169, fig. S11A), similar to the counterparts on PCT64 LMCA (fig. S11B). However, the HCDR3 of RM038 was less extended towards the 3-fold axis than PCT64 LMCA. Notably, the sulfated tyrosine within DDY motif of RM038 interacted around the top surface of the 3-fold axis, including V127, G128, and the base of the N160 glycan on the same protomer (fig. S11C), instead of being buried inside like PCT64 LMCA, which interacted with T123 and P124 (fig. S11D). Rotation of the structure by 90° revealed that the HCDR3 of RM038 formed a second lobe that interacted to strand C of one of the protomers (Fig. 7, D and E, fig. S11E). A similar second lobe was present in the apo crystal structure (fig. S11, F and G). Thus, the HCDR3 of RM038 had similarities to the hammer-head motif on the HCDR3 of the Apex bnAb PG9 (fig. S11H), but with the heavy and light chains flipped when RM038 is aligned with PG9 liganded structures (18) (fig. S11I). Overall, the HCDR3 of RM038 exhibited hybrid features of PCT64 and PG9 when interacting with the apex epitope of ApexGT6 (Fig. 7, C–E). In addition to the protruding HCDR3 interacting at the center of the apex, the V2b hydrophobic loop also remains the focal point of a hydrophobic pocket created at the interface of the heavy and light chains of RM038 (Fig. 7F and fig. S11J). Several polar and acidic SHMs on HCDR1 and HCDR2 might enhance binding to the N160 glycans (Fig. 7G), which commonly occurred during Apex bnAb maturation (23, 50, 53–55).

**Fig. 8.**
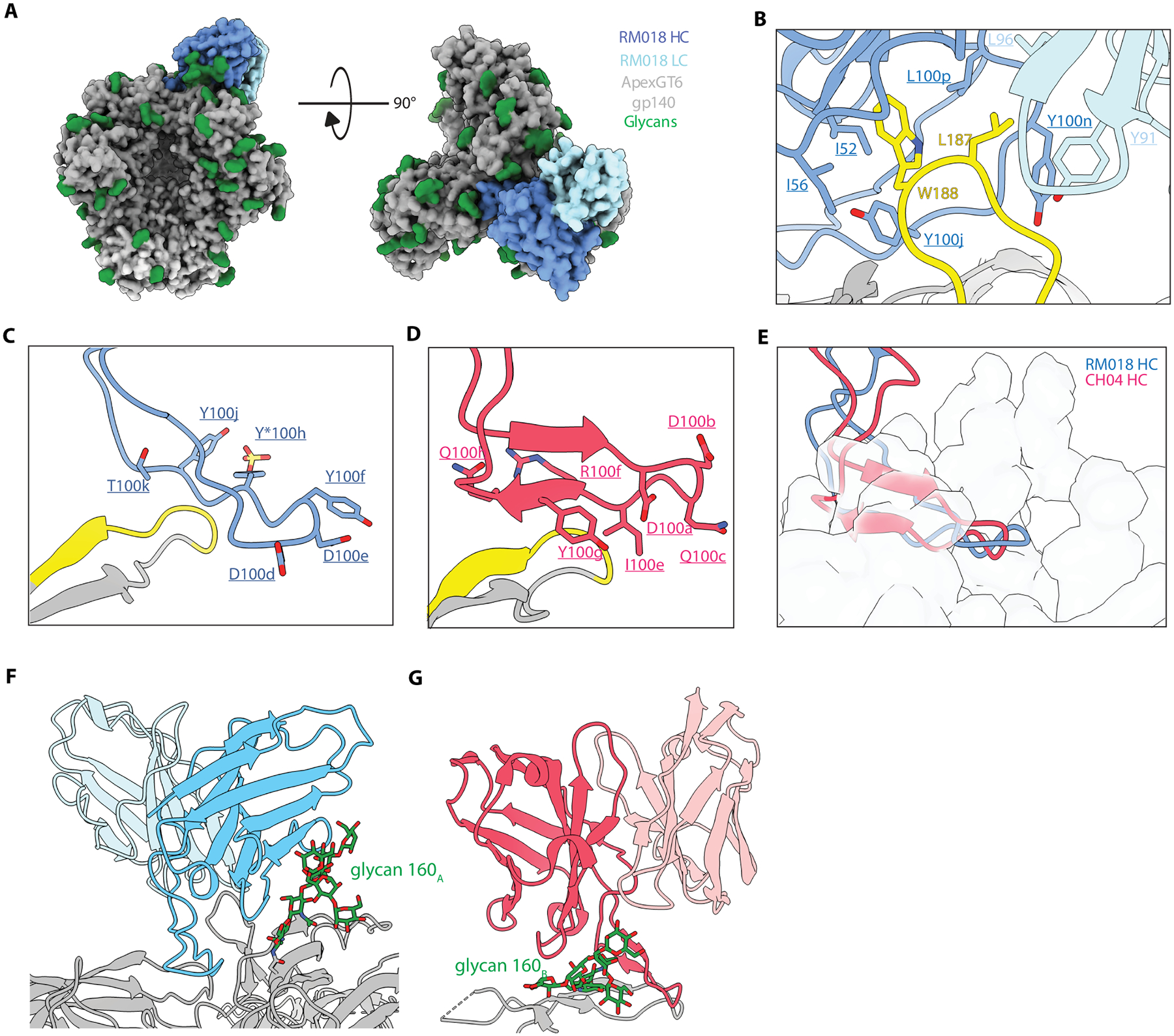
ApexGT6 induced mAbs showed similar structural features of CH01–04. **(A)** Top-down and side views of RM018 (blue) binding at the trimer 3-fold axis of ApexGT6 (grey). Glycans are colored as green spheres. **(B)** Zoomed-in view of the hydrophobic pocket (residues showed as sticks) interactions with the engineered hydrophobic V2b loop (highlighted as yellow cartoon, with hydrophobic mutations depicted as sticks). **(C)** Side view of the HCDR3 of RM018 (blue) and strand C (166–170 residues colored as yellow) on gp120_A_ of ApexGT6, illustrating the CH04-like feature, with key residues depicted as sticks. **(D)** Side view of the HCDR3 of CH04 (red, PDB:5ESZ) and strand C (166–170 residues colored as yellow) on the V1V2 scaffold, with key residues depicted as sticks. **(E)** Superimposed liganded structures of RM018 and CH04. The structures were aligned using residues 156–174 on strand C, with ApexGT6 showed as white surface. **(F)** Side view of the VH gene segment of RM018 (blue cartoon) interacting with the N160 glycan (green sticks) on the trimer apex of ApexGT6 (grey). **(G)** Side view of the VH gene segment of CH04 (red cartoon) interacting with the N160 glycan (green sticks) on the trimer apex of ApexGT6 (grey).

The structure of the second mAb (RM018) in complex with ApexGT6 also showed the expected 1:1 stoichiometry and a tilted angle of approach relative to the 3-fold axis (Fig. 8A), with a similar heavy and light chain binding mode as RM017 and RM038 (fig. S12A). The heavy and light chains also formed a hydrophobic pocket around V2b mutations as observed for RM017 and RM038 (Fig. 8B and fig. S12B). The HCDR3 of RM018 formed an extended loop that interacted with strand C on one protomer of the trimer and with the DDY motif at the tip of the long HCDR3 (Fig. 8C and fig. S12C). Unlike PCT64 LMCA, RM017 or RM038, the DDY tip on the HCDR3 of RM018 interacted around the Apex 3-fold axis in a more parallel manner, with the tyrosine within the DDY motif being unsulfated (fig. S12, D and E). This binding mode is similar to that of the Apex bnAb CH01–04 (55) (Fig. 8, D–E). In the unliganded RM018 crystal structure, the HCDR3 adopted a beta-hairpin conformation (fig. S12F) and showed greater extension compared to the liganded form (fig. S12G), suggesting that the CH01–04-like conformation may be induced upon binding to the Apex epitope. Beyond the HCDR3s, RM018 also exhibited extensive glycan interactions with the heavy chain framework regions (FR) 2 and 3, as well as CDR2s, similar to CH04 but with the heavy chain and light chain positions reversed (Fig. 8, F and G).

In summary, we found that Apex bnAb-like BCRs induced by ApexGT6 exhibited structural features similar to those of three human Apex bnAbs. This suggested that ApexGT6 might be capable of eliciting multiple Apex bnAb-related responses simultaneously.

### Alternative J_H_ gene usage makes CH01–04 promising Apex bnAb targets for germline-targeting vaccine design

In a previous study (18), the CH01–04 class of bnAbs was not prioritized for Apex vaccine design due to its low precursor frequency arising from its use of a rare J_H_ gene (J_H_2). Our finding that RM018 had a binding mode sharing features with CH01–04 bnAbs prompted us to revisit the idea of targeting this class of Apex bnAbs. We calculated that CH01–04-class BCRs utilizing a more common J_H_ gene (J_H_4) would have a substantially higher precursor frequency (median frequency of 0.30 per million naïve B cells in 13 out of 14 donors, fig. S13A) compared to using J_H_2 (median frequency of 0.09 per million naïve B cells in 2 out of 14 donors). This frequency surpassed that of PG9/PG16, which were previously considered to have the second-highest precursor frequency in the human immunoglobulin repertoire among long HCDR3-dependent V2 apex bnAbs (fig. S13A). We then produced variant CH01–04-like antibodies using the more common J_H_4 gene and evaluated their neutralization capacity. We found that the J_H_4-variant of CH04 had slightly improved neutralization breadth and potency (74% breadth with a geometric mean IC50 of detected 0.39 μg/mL) compared to WT CH04 (70% breadth with a geometric mean IC50 of 0.53 μg/mL) (fig. S13B). This was tested over a panel of 23 viruses, including strains sensitive to CH01–04 (fig. S13, B and C). Therefore, we propose that the J_H_4-variant CH01–04-class represents an attractive target for vaccine induction. Eliciting a CH01–04 response in addition to a PCT64-class response would potentially expand the breadth of neutralization induced, because CH01–04 can neutralize some HIV strains that PCT64 cannot. It is not entirely surprising that ApexGT6 can elicit a CH01–04-like response in NHPs, given that it can bind to the iGLs and a human NGS precursor of CH04 with appreciable affinty (fig. S13, D and E). We concluded that J_H_4-variant CH01–04 bnAbs are an important vaccine target and ApexGT6 has the potential to prime precursors for this class of bnAb.

## Discussion

Apex bnAbs were the first to be discovered among the new generation of high potency and high breadth HIV bnAbs (56), and they represent canonical examples of antibodies with long HCDR3-dependent binding modes. Developing vaccine strategies to induce Apex bnAbs remains a major goal within the HIV vaccine field, in large measure because of the relatively low levels of SHM typically required for Apex bnAbs to develop breadth and potency. Beyond HIV, learning how to consistently induce antibodies with very long HCDR3s could allow for deeper study of the processes of long HCDR3 creation and maturation, and could open doors for vaccine development against other recessed epitopes on other pathogens. We found that two bolus immunizations of the germline-targeting priming immunogen ApexGT6 initiated strong Apex bnAb-related precursor responses in all immunized RMs, using soluble protein or mRNA-encoded membrane-anchored trimer formats. These Apex bnAb-related precursor responses were polyclonal, featuring diverse junctional regions within the HCDR3, meeting a key additional requirement for successful germline targeting priming (7, 8, 11–15, 30, 57, 58). Apex bnAb-related B_mem_ cells were found at a frequency approximately one-third of that observed for CD4 binding site-specific VRC01-class B_mem_ cells induced by the eOD-GT8 60mer protein adjuvanted with AS01B in a recent human clinical trial (19). Furthermore, these responses showed improved binding to Apex epitopes closer to native over time, suggesting that the ApexGT6-induced bnAb-related precursor responses accumulated SHM on a path toward bnAb development. This study demonstrates that a vaccine can consistently stimulate the production of long-HCDR3 Apex bnAb-related responses in an outbred animal model, which represents a major advance toward development of a vaccine to induce Apex bnAbs.

ApexGT6 consistently induced Apex bnAb-like precursor responses (with a DDY motif near the middle of the long HCDR3) but did not induce precursors encoded by D_H_3–41. This might be because DDY precursors have higher precursor frequencies than D_H_3–41 precursors in RMs. Furthermore, the acidic nature of the DDY motif might give it an affinity advantage over D_H_3–41 precursors, as the negative charge could facilitate the binding of these precursors to the positively charged strand C residues on ApexGT6. Although humans lack a D gene encoding DDY, we found a substantial frequency of Apex bnAb-like precursors containing a DDY motif near the middle of the long HCDR3 among HIV-negative human donors. Hence, given the ability of ApexGT6 to induce such DDY-encoding precursors in RMs, we hypothesize that ApexGT6 might be capable of initiating Apex bnAb-like (DDY) precursor responses in humans. ApexGT6 also has potential to prime Apex epitope-specific D_H_3–3 precursors in humans, because ApexGT6 has detectable affinity for some human NGS precursors encoded by D_H_3–3, and humans have a higher frequency of D_H_3–3 precursors compared to DDY precursors.

Cryo-EM revealed that some of the elicited Apex bnAb-related HCDR3s possessed combined elements from two Apex bnAbs, PG9 and PCT64, when interacting with the trimer immunogen. Notably, PG9 has much higher neutralization breadth and potency than PCT64 (59, 60). Our findings suggest that ApexGT6 could potentially induce hybrid responses with the high precursor frequency of PCT64 and the high neutralization potency of PG9. This hybrid HCDR3 structure has been observed in humans (50), implying that these findings might be relevant to human immunization. Furthermore, the RM apex Abs exhibited HCDR3 features similar to those of the three prototype bnAbs (PCT64, PG9, CH01–04) that have the highest precursor frequencies among long HCDR3-dependent Apex bnAbs in the human immunoglobulin repertoire. This implies that our studies may be applicable in a human vaccination setting. Our results with ApexGT6 suggest that one ApexGT trimer could potentially target all three classes of long HCDR3-dependent Apex bnAbs, expanding the precursor frequency and neutralization breadth of an Apex epitope-specific response.

In the cryo-EM structures, we observed a dependency on the engineered V2b loop, where several germline targeting mutations are located. Our SPR results suggested that after the second immunization, over half of the Apex bnAb-like mAbs could bind to the ApexGT6 with a WT V2b loop. This implies that sequential heterologous boosting immunizations might guide the Apex bnAb-like responses to eliminate dependence on the V2b loop.

Compared to adjuvanted-protein immunization, mRNA-LNP immunization to deliver membrane-anchored trimer exhibited a higher percentage of Apex epitope-specific responses and stimulated Apex bnAb-related BCRs with longer HCDR3s and a higher number of different lineages. These benefits appeared to offset the higher immunogenicity of adjuvanted-protein immunization under the dosing conditions tested here, resulting in similar frequencies of Apex bnAb-related B_mem_ precursors and SHM. We were not able to compare GC dynamics between the mRNA and adjuvanted-protein groups. Nevertheless, we observed an increase in the frequency of Apex bnAb-related B_mem_ cells in blood after the second immunization in the mRNA group, which suggested that two vaccinations with mRNA-LNPs encoding membrane-anchored ApexGT6 might be necessary to facilitate subsequent heterologous boosting needed to drive further maturation toward bnAb development. Alternately, the second immunization might be serving mostly to drive pre-existing, already-matured GC responses into the periphery as memory B cells, facilitating their detection but perhaps not being required prior to a heterologous boost. Future studies will be needed to examine this question.

Quaternary epitopes like the Apex of the HIV Env trimer are often targeted by bnAbs against various pathogens including respiratory syncytial virus (RSV) (61), dengue virus (62), Ebola Virus (63), and SARS-CoV-2 (64). However, engineering these epitopes for vaccine design presents challenges. Mutations at the interface of adjacent protomers can cause instability and misfolding of multimeric proteins. Additionally, for asymmetric epitopes, a mutation on one protomer that provides favorable interactions to an antibody may result in an unfavorable interaction between that antibody and the other protomer. The results of this study indicate promise for design of immunogens that induce bnAb precursors to quaternary epitopes for other pathogens.

One limitation of this study is that limited direct data were obtained on GCs after ApexGT6 mRNA-LNP immunizations. We infer that active GCs were present in these animals, due to similar rates of SHM and affinity maturation seen in the mRNA group when compared with protein immunization animals. Our difficulties in isolating antigen-specific B_GC_ cells from FNAs following mRNA-LNP intramuscular deltoid vaccination in RMs were consistent with findings of other RM studies (44, 65). Additionally, while we used strict minimum length criteria for defining an Apex bnAb-related HCDR3, based on the known Apex bnAbs, it remains uncertain whether those with HCDR3s longer than 20 aa but shorter than 24 aa have the potential to become apex bnAbs. Follow-up studies will be required to understand the potential of Abs with length 22 to 23 aa HCDR3s for bnAb development. Moreover, the human precursor frequencies we provided in this study were based on structurally informed sequence-based precursor definitions. However, we did not account for binding affinities or the potential effect on binding affinities by sequence determinants within the non-templated HCDR3 junctions. Therefore, the percentage of the bioinformatically defined precursors that can bind with sufficient affinity to ApexGT6 remains unknown. Furthermore, this study primarily focused on priming Apex bnAb-like precursors, not on eliciting bnAbs through sequential immunization. We did not expect or aim to elicit heterologous neutralization after delivering a priming immunogen, because the priming immunogen still contains several germline-targeting mutations. These mutations, designed to recruit precursors, are not found in most wild-type HIV strains. However, we observed an increased affinity of the Apex bnAb-like precursor monoclonal antibodies to trimers with a more native-like Apex epitope over time. These trimers with more native Apex epitopes might potentially serve as heterologous boosting immunogens. We hypothesize that bnAb induction may require two or three heterologous boosts with successively more native-like trimers.

## Materials and Methods

### Study design

This study primarily aimed to assess whether germline-targeting vaccination could consistently elicit bnAb-like precursor responses targeting the HIV Env Apex region in outbred primates. To achieve this goal, we immunized rhesus macaques with ApexGT6, a germline-targeting trimer with affinity for additional PCT64- and PG9-class precursors. We administered it in two forms: as an adjuvanted soluble protein or as an mRNA-encoded membrane-anchored protein. Throughout the study, we assessed epitope-specific responses in serum, germinal centers, and memory cells. Binding assays were conducted to examine epitope specificity and acquired affinity maturation through immunization. Structural analyses were performed to investigate how the macaque Apex bnAb-like BCRs induced by ApexGT6 recognize the Apex epitope, and to validate whether they exhibited HCDR3 features similar to human Apex bnAbs. For animal experiments, the investigators were not blinded to the group identities.

### Cell lines

This study used HEK 293F cells, for production of soluble proteins in suspension and for cell-surface expression and antigenicity testing; HEK 293T cells (ATCC) were used to produce viruses; rtTA3G-expressing HEK 293T cells were used for mammalian display directed evolution.

For virus production, HEK 293T cells (ATCC) were maintained in complete DMEM (Gibco), supplemented with 10% FBS, 2 mM L-glutamine (Gibco), and 1% Pen-Strep. TZM-bl cells (NIH AIDS Reagents Program) were maintained in complete DMEM and used as target cells in pseudovirus neutralization assays.

For mammalian display, HEK 293T cells were cultured at 37°C in Advanced DMEM (Gibco) supplemented with 5% FBS (Gibco), GlutaMAX (Gibco), 2-mercaptoethanol (Gibco) and Antibiotic-Antimycotic (Gibco); rtTA3G-expressing HEK 293T cells were cultured under the same conditions but with the addition of 10 μg/mL blasticidin (Gibco).

All cell lines were maintained at 37°C in a humidified atmosphere of 5% CO2. Sex of both cell lines was female. Cell lines have not been authenticated.

### DNA gene synthesis and cloning

Five types of gene constructs were used in this study. Procedures were similar to those previously used (18) with some differences noted. Recombinant trimers and antibodies were synthesized at GenScript, Inc. Trimers were cloned into pCW between the signal sequence and a C-terminal GTKHHHHHH tag using the AgeI and KpnI cloning sites. Trimer probes were cloned into pHLsecAvi2 between the signal sequence and a C-terminal flexible linker (GTGGSGGSG), followed by an Avi tag (LNDIFEAQKIEWHE) connected through a flexible linker with a his tag (GGSGGSHHHHHH) using the AgeI and KpnI cloning sites. Antibody heavy chains were cloned into pCW-CHIg-hG1 between the leader and the IgG1 human constant domain using the EcoRI and NdeI cloning sites. Kappa chains were cloned into pCW-CLIg-hk between the leader and the human kappa constant region using the EcoRI and BsiWI cloning sites. Lambda chains were cloned into pCW-CLIg-hL2 between the leader sequence and the last aa in lambda FW4 using the EcoRI and AvrII cloning sites.

Genes used for library design were cloned into pENTR via Gibson assembly. This included a C-terminal cMyc epitope followed by a platelet-derived growth factor receptor (PDGFR) transmembrane domain. Error-prone PCR libraries were constructed using the GeneMorph II Random Mutagenesis Kit (Agilent). Combinatorial NNK libraries were ordered as ultramers from IDT, using ambiguous nucleotides at the combinatorial positions. Alternating complement and reverse complement ultramers were assembled with outer primers, overlapping an insertion site by 30 bp.

### ApexGT immunogen design

Lentiviral mammalian display and directed evolution were performed as described previously (12), with modifications to sorting probes and libraries. The epPCR library, starting with the BG505.ApexGT2 based construct, introduced random mutations at positions 86–229 (HXBC2 numbering). The combinatorial NNK library, starting with the BG505.ApexGT5 based construct, had three combinatorial NNK positions K121, T128, and K168 using IDT.

Cells were incubated with PCT64 LMCA IgG and B6 Fab with a V5 tag, washed with FACS buffer, and then stained with fluorescein isothiocyanate (FITC)-labeled α-cMyc (Immunology Consultants Laboratory), R-Phycoerythrin AffiniPure F(ab')₂ Fragment Goat Anti-Human IgG, Fcγ fragment specific (Jackson), and V5-Tag Antibody | SV5-Pk1 (Bio-Rad) (Table S4). Cells were sorted on a BD Influx (BD Biosciences) FACS sorter. Approximately 10K of the desired gated cells were collected and expanded for ∼ one week in the presence of puromycin and blasticidin before the next round of enrichment was carried out. The genomic DNA for the final enriched libraries was extracted and PCR amplified using partial adapters recommended by GeneWiz “EZ amplicon”. We determined enriched mutations as described previously (12).

### ApexGT protein expression

BG505-based ApexGT variants were cloned into pCW as described above and transformed into DH5α cells following the manufacturer's protocol. Plasmids were maxi-prepped using a BenchPro 2100 (Thermo Fisher Scientific). Trimer plasmids were co-transfected with furin in a 2:1 ratio into 293F cells cultured in FreeStyle media (Life Technologies) using polyethylenimine (PEI, Polysciences, Inc.; 1:3 DNA:PEI ratio) as a transfection reagent in Opti-MEM^™^ reduced serum medium (Thermo Fisher Scientific, Cat# 31985070). Proteins were harvested from the supernatant after 7 days of incubation at 37°C. For non-tagged trimers, proteins were purified through lectin affinity chromatography using 7.5 mL of lectin beads from Vector Laboratories (#AL-1243) per 1L of protein supernatant. This was done using a disposable plastic column from Bio-Rad (#732–1010). Trimer fractions were further isolated using size exclusion chromatography (SEC) on a Superdex 200 PG SEC column (Cytiva, Cat 28–9893-35) on an ÄKTA Pure 25L HPLC. For his-tagged trimers, proteins were purified by Ni++ affinity chromatography followed by SEC. For biotinylated probes, proteins were expressed with a his-tag and avi-tag, purified by Ni++ affinity chromatography followed by SEC, and biotinylated using a BirA biotin-protein ligase reaction kit (Avidity, Cat# BirA500) according to the manufacturer's instructions. The oligomeric state of the trimers was then confirmed by size exclusion chromatography-multi-angle light scattering using the DAWN HELEOS II multi-angle light scattering system with Optilab T-rEX refractometer (Wyatt Labs). The final proteins were diluted in 1x TBS and stored at −80°C.

### Antibody expression and purification

Paired HC and LC variable region sequences from selected RM BCRs were gene synthesized and inserted into human IgG1 constant region expressing vector pCW-CHIg-hG1 and human kappa or lambda expressing vectors pCW-CLig-hk or pCW-CLig-hL2, respectively. Heavy and light chains along with TPST1 were transfected with PEI at a 3:1:1:1 PEI:heavy:light:TPST1 ratio into Freestyle 293F cells. Supernatants were harvested on day 7, and batch binding occurred overnight at 4°C with Protein A resin (Thermo Fisher Scientific, Cat# 20334), on a rocker. After elution with IgG elution buffer (0.1 M Glycine pH 2.7, Thermo Fisher Scientific), antibodies were buffer-exchanged into 1× PBS and then concentrated using a 50k MWCO concentrator (Millipore).

### Surface plasmon resonance (SPR)

We measured kinetics and affinity of antibody-antigen interactions on Carterra LSA using a low capture IgG method as previously described (22).

### Antigenic profile with enzyme-linked immunosorbent assay (ELISA)

96-well plates were coated overnight at 4C with 6x-His Epitope Tag Rabbit Antibody (Genscript, Cat. No. A00174, Table S4) at 2ug/mL in PBS. Plates were washed 3 times with PBS, 0.2% Tween (PBS-T), and blocked with 5% milk and 1%FBS in PBS-T for 1h. Subsequently, 2ug/mL of the purified His-tagged trimers was added for 2h in PBS, after which the plates were washed three times with PBS-T. Serial dilutions of antigenic profiling mAbs in 1% FBS PBS-T were added to the plates for 1 h, after which the plates were washed again three times with PBS-T before the addition of anti-human conjugated peroxidase at 1:5000 for 1 h. After four final washes, binding was detected by the addition of TMB substrate and measured by absorbance at 450 and 570nm. Background subtraction was performed by subtracting the 570 nm value from the corresponding 450 nm value. Data were subsequently analyzed in Prism (Prism v10.2; GraphPad Software).

### Differential scanning calorimetry (DSC)

DSC experiments were performed on a MicroCal VP-Capillary differential scanning calorimeter (Malvern Instruments) as previously described (12).

### Glycan analysis

Glycan analysis, including Proteinase K treatment and deglycosylation, LC-MS/MS, data processing, and the DeGlyPHER (38) method were carried out as previously described (25).

### Animals and immunizations

Twenty-four healthy adult Indian-origin rhesus macaques (*Macaca mulatta*) were used in this study. The animals were housed at the Emory National Primate Research Center (ENPRC). All procedures were approved by the Emory University Institutional Animal Care and Use Committee (IACUC) under protocol 202000087. Animal care facilities are accredited by the U.S. Department of Agriculture (USDA) and the Association for Assessment and Accreditation of Laboratory Animal Care (AAALAC) International.

Four groups of six RMs were immunized at weeks 0 and 8. In each group All immunization were split between two limbs in the deltoid area. One group received subcutaneous immunizations 100 ug of ApexGT6 soluble trimer protein (ApexGT6 congly gp140) and 750 ug of SMNP adjuvant. Another group received intramuscular (IM) immunizations of 100 μg of mRNA-LNP encoding cleavage-independent membrane-bound ApexGT6 (ApexGT6 L14 gp151). A third group was immunized IM with 100 ug of soluble trimer BG505 MD39.3 and 750 ug of SMNP. The final group received 100ug of membrane-bound BG505 MD39.3 gp151 mRNA-LNP via IM injections.

### Tissue collection and processing.

Blood was collected throughout the study in NaCitrate CPT tubes for peripheral blood mononuclear cells (PBMCs) and plasma isolation and frozen. Serum was collected via serum clot tubes and frozen. Lymph node fine needle aspirates were collected, processed, and frozen as previously described (45).

### Analysis of plasma by ELISA

96-well plates were coated overnight at 4C with 6x-His Epitope Tag Rabbit Antibody (Genscript, Cat. No. A00174, Table S4) at 2ug/mL in PBS. Plates were washed 3 times with PBS, 0.2% Tween (PBS-T), and blocked with 5% milk and 1%FBS in PBS-T for 1h. Subsequently, 2ug/mL of the purified His-tagged trimers was added for 2h in PBS, after which the plates were washed three times with PBS-T. Serial dilutions (3-fold factor) of serum (1:100 dilution) in 1% FBS PBS-T were added to the plates for 1 h, after which the plates were washed again three times with PBS-T before the addition of anti-human conjugated peroxidase at 1:5000 for 1 h. After four final washes, binding was detected by the addition of TMB substrate and measured by absorbance at 450 and 570nm. Background subtraction was performed by subtracting the 570 nm value from the corresponding 450 nm value. Data were subsequently analyzed in Prism (Prism v10.2; GraphPad Software).

### Negative stain Electron Microscopy Polyclonal Epitope Mapping (nsEMPEM)

For the protein group, we used the same soluble protein immunogen, ApexGT6 congly, as antigen samples. For the mRNA group, we used the soluble protein truncated at D664 (gp140) version of ApexGT6 L14 gp151, along with the 5CC variant containing two additional disulfide bonds: 49C-555C (intra-protomer) and 73C-561C (inter-protomer). These disulfide bonds prevented trimer degradation during plasma incubation of nsEMPEM of the mRNA group.

After heat inactivation at 56°C for 1 hr, IgG was purified from plasma samples on an AKTA Pure Protein Purification System (Cytiva) on a HiTrap MabSelect PrismA column (Cytiva). Purified IgG was then digested into Fab using liquid papain as described in the EMPEM protocol (66). Once isolated, 750 μg of Fab was complexed with 15 μg of Apex GT6 trimers and left at RT overnight. The following day, trimer-Fab complexes were purified and isolated from unbound Fabs via SEC (Superdex Increase 200; Cytiva). Samples were then concentrated using 10 kDA Amicon^®^ concentrators (Millipore).

Carbon-coated Cu-400 mesh grids (Electron Microscopy Sciences) and Nano-W^™^(Nanoprobes) stain were using for negative stain sample grid preparation. The grids were glow discharged with a PELCO easiGLOW (Ted Pella Inc) before sample application. Immediately before sample application, samples were diluted to 0.02 μg/μL in TBS (50 mM Tris pH 7.4, 150 mM NaCl) before 3 μL was applied to the grid. After blotting off the sample, 3 μL of Nano-W^™^ was applied, held for 7 seconds, blotted and waited for 10 seconds, another 3 μL of Nano-W^™^ was applied and held for 15 seconds before a final blot.

Image collection was done on an FEI Talos microscope (Thermo Fisher Scientific) at 73,000x magnification and 1.98-pixel size using Leginon (67) and initial data processing was conducted through Appion (68). 2D and 3D Classification and 3D refinement was done through Relion v3.0(69). EM maps were visually presented using UCSF Chimera (70) and Segger (71) was used for segmentation.

### Cryo-EM sample preparation

For RM038, 200 ug ApexGT6 trimer was complexed overnight at RT with 300 ug of Fabs of RM038 and RM20A3. The sample was then purified via SEC (Superdex 200 Increase; Cytiva) to isolate the complex and remove excess, unbound Fabs. For RM018 and RM017, 200 μg of Apex GT6 trimer was complexed overnight with 136.4 μg of RM20A3 Fab at RT and then the following day, 68.2 μg IgG of RM018 or RM017 was added to the sample and allowed to complex with ApexGT6 and RM20A3 for 30 minutes. Samples were then concentrated to 4–5 mg/ml. A Vitrobot Mark IV (Thermo Fisher Scientific) was used to vitrify the samples with the chamber temperature set to 10°C and humidity at 100%. Quanitifoil Cu 1.2/1.3 300C-mesh (Quanitfoil Micro Tools GmbH) were glow discharged for 25 s at 15 mA by a PELCO easiGLOW (Ted Pella Inc) prior to sample application. Before samples were applied to the glow discharged grids, lauryl maltose neopentyl glycol (LMNG) was added to a final concentration of 0.005 mM. Blot times varied from 5–7 seconds before plunge freezing into liquid ethane, previously cooled by liquid nitrogen.

### Cryo-EM data collection

Samples were collected on a 200 kV Glacios (Thermo Fisher Scientific) with an autoloader and mounted Falcon 4 detector (Thermo Fisher Scientific). EPU software (Thermo Fisher) was used for automated data collection. Micrographs were collected at a magnification of 190,000x with a resulting pixel size of 0.725.

### Cryo-EM data processing

CryoSPARC (72) Live was used for preprocessing of the micrographs and further processing was done in CryoSPARC 3.2 and 4.4. For the monoclonals, particles were picked using blob and template picker before being iteratively 2D classified to remove junk particles. Once good particle stacks were obtained, low resolution, initial 3D volumes were generated using Ab Initio in CryoSPARC. These initial maps were then used to refine the volumes using particle information to generated high-resolution maps after multiple rounds of refinements (homogenous, non-uniform, and local).

### Model building and figure preparation

Initial models for Apex GT6 and RM20A3 and RM038 were generated using UCSF Chimera Modeller (73) using PDB: 7T74. For RM018 Fv, initial model was generated through SAbPred (74) ABodyBuilder-ML (75) (V_H_ framework: 5i18; V_L_ framework: 7tp4; V_H_-V_L_ orientation: 5n4j). SAbPred ABodyBuilder-ML also generated the initial model for RM017 (V_H_ framework: 7tp4; V_L_ framework: 7e9o; V_H_-V_L_ oritentation: 5vzy). Iterative rounds of model building, and refinements were done through Coot v0.9.8.8 (76), Phenix v1.20.1 (77), and Rosetta (78). Structures were validated using Molprobity (79) and EMRinger (80). Preparations for figures were done through ChimeraX (81).

### Crystallization of unliganded Fabs

For the crystallization of unliganded Fabs, protein samples of RM018, RM014, and RM038 were concentrated to 13 mg/mL in a buffer containing 20 mM Tris, 150 mM NaCl, pH 8.0. Crystallization trials were performed using a Rigaku CrystalMation robotic system and JCSG 1–4 crystal screens at 4°C and 20°C. RM018 Fab crystals were obtained at 20°C in a solution containing 0.16 M zinc acetate, 0.108 M sodium cacodylate (pH 6.5), 14.4% PEG 8000, and 20% glycerol. RM038 Fab crystals were grown at 20°C in a solution containing 0.1 M imidazole (pH 8.0) and 1.0 M sodium citrate. Crystals of RM014 Fab were grown at 20°C in a solution containing 0.2 M Calcium acetate 0.1 M Tris (pH 7.0), and 20% PEG 3000. Crystals were harvested and cryoprotected by adding 15% ethylene glycol to the reservoir crystallization solution before being quickly plunged into liquid nitrogen for storage prior to data collection.

### X-ray data collection and structure determination

Diffraction data for RM018 were collected at the National Synchrotron Light Source II on beamline 17-ID-1 AMX, while data for RM014 and RM038 were collected at the Stanford Synchrotron Radiation Light Source on beamline BL12–2. The crystals of RM018, RM014, and RM038 diffracted to resolutions of 3.30 Å, 1.88 Å, and 1.98 Å, respectively. Data indexing, integration, and scaling were performed using HKL2000 (82). The structures were solved by molecular replacement using Phaser in Phenix (83). Subsequent model building and refinement were performed using Coot and Phenix.refine (76), (84). Structural quality was evaluated with MolProbity (85), and additional validation was carried out using the PDB validation server. The data collection and refinement statistics are summarized in Supplementary Table S3.

### Flow cytometry and sorting

ApexGT6 and ApexGT6.KO probes were prepared by mixing with fluorophore-conjugated streptavidin in 1× PBS at room temperature in 1/3 increments over 45 minutes. LN FNA or PBMC samples were thawed and recovered in fully supplemented RPMI (10% (v/v) FBS, 1× pen/strep, 1× GlutaMAX). Live cells were counted and stained with ApexGT6.KO and ApexGT6 probes sequentially for 20 minutes each at 4°C, followed by staining with an antibody master mix and individual anti-human hashtag antibodies at a concentration of 2.5 μg per 5 million cells for 30 minutes at 4°C (Table S4). PBS with 2% (v/v) FBS was used as FACS buffer. Samples were sorted on a FACSymphony S6 (BD Biosciences). Due to dim staining by our CD71 clone, we chose to sort all CD20^+^CD38^−^ ApexGT6^++^ LN B cells to maximize the number of sequences obtained (fig. S2C, fig. S3). This population should contain both GC and memory B cells. CD20^+^IgD^−^ B_mem_ were sorted from PBMCs (fig. S2D, fig.S3). For LN samples, we also sorted ApexGT6^−^ populations in addition to the ApexGT6^++^ populations to allow direct comparison of antigen-specific and non-specific B cells. The following reagents were used during staining: Alexa Fluor 647 streptavidin (Invitrogen, Cat# S21374), BV421 streptavidin (BioLegend, Cat# 405225), BV650 streptavidin (BioLegend, Cat# 405232), eBioscience Fixable Viability Dye eFluor 506 (Invitrogen, Cat# 65–0866-18), eBioscience Fixable Viability Dye eFluor 780 (Invitrogen, Cat# 65–0865-14), mouse anti-human CD3 APC-Cy7 (SP34–2, BD Biosciences, Cat# 557757, RRID: AB_396863), mouse anti-human CD4 APC-Cy7 (SK3, BD Biosciences, Cat# 566913, RRID: AB_2739681), mouse anti-human CD8a APC-eFluor780 (RPA-T8, Thermo Fisher Scientific, Cat# 47–0088-42, RRID: AB_1272046), mouse anti-human CD14 APC-Cy7 (M5E2, BioLegend, Cat# 301820, RRID: AB_493695), mouse anti-human CD16 APC-eFluor780 (eBioCB16, Thermo Fisher Scientific, Cat# 47–0168-42, RRID: AB_11220086), mouse anti-human CD20 BV570 (2H7, BioLegend, Cat# 302332, RRID: AB_2563805), mouse anti-human CD20 PerCP-Cy5.5 (2H7, BioLegend, Cat# 302326, RRID: AB_893283), mouse anti-human CD27 PE-Cy7 (O323, BioLegend, Cat# 302838, RRID: AB_2561919), mouse anti-human CD38 APC (OKT10, NHP Reagents, Cat# PR-3801, RRID: AB_2819277), mouse anti-human CD71 FITC (L01.1, BD Biosciences, Cat# 347513, RRID: AB_400316), goat anti-human IgD FITC (polyclonal, Southern Biotech, Cat# 2030–02, RRID: AB_2795624), mouse anti-human IgG PE-Cy7 (G18–145, BD Biosciences, Cat# 561298, RRID: AB_10611712), mouse anti-human IgG BV786 (G18–145, BD Biosciences, Cat# 564230, RRID: AB_2738684), mouse anti-human IgM PerCP-Cy5.5 (G20–127, BD Biosciences, Cat# 561285, RRID: AB_10611998), mouse anti-human IgM BV605 (G20–127, BD Biosciences, Cat# 562977, RRID: AB_2737928) and TotalSeq-C0953 PE streptavidin (BioLegend, Cat# 405265). TotalSeq-C anti-human Hashtag antibodies 1–10 (LNH-94 and 2M2, BioLegend) were used as follows: Hashtag 1 (Cat# 394661, RRID: AB_2801031), Hashtag 2 (Cat# 394663, RRID: AB_2801032), Hashtag 3 (Cat# 394665, RRID: AB_2801033), Hashtag 4 (Cat# 394667, RRID: AB_2801034), Hashtag 5 (Cat# 394669, RRID: AB_2801035), Hashtag 6 (Cat# 394671, RRID: AB_2820042), Hashtag 7 (Cat# 394673, RRID: AB_2820043), Hashtag 8 (Cat# 394675, RRID: AB_2820044), Hashtag 9 (Cat# 394677, RRID: AB_2820045), and Hashtag 10 (Cat# 394679, RRID: AB_2820046). For sequencing, Indexed V(D)J, Feature Barcode, and GEX libraries of sorted LN FNA samples were prepared according to the protocol for Single Indexed 10X Genomics V(D)J 5' v.1.1, with Feature barcoding kit (10X Genomics). For sorted PBMC samples, Indexed V(D)J, Feature Barcode and GEX libraries were prepared using the Dual Indexed 10X Genomics V(D)J 5’ v.2 with Feature barcoding kit (10X Genomics). Custom primers were designed to target RM BCR constant regions. Forward primers were used at a final concentration of 1 μM and reverse primers at 0.5 μM, each per 100 μl of PCR reaction. Libraries were pooled and sequenced on a NovaSeq Sequencer (Illumina). A wetting failure occurred during the 10x Genomics library preparation for the week 10 protein PBMC samples, associated with cell health. Additionally, limited cell availability for three animals precluded further attempts. Consequently, no sequence data were obtained from these samples. Overall, sequence recovery from our PBMC samples was lower than expected, likely due to suboptimal cell health.

The list of Oligonucleotides from Integrated DNA Technologies is as follows:

PCR 1 forward: AATGATACGGCGACCACCGAGATCTACACTCTTTCCCTACACGACGCTC
PCR 1 reverse: AGGGCACAGCCACATCCT, TTGGTGTTGCTGGGCTT, TGACGTCCTTGGAAGCCA, TGTGGGACTTCCACTGGT, TGACTTCGCAGGCATAGA.
PCR 2 forward: AATGATACGGCGACCACCGAGATCT
PCR 2 reverse: TCACGTTGAGTGGCTCCT, AGCCCTGAGGACTGTAGGA, AACGGCCACTTCGTTTGT, ATCTGCCTTCCAGGCCA, ACCTTCCACTTTACGCT.

### BCR sequencing and processing

Full-length V(D)J reads were assembled with CellRanger v.3.0, utilizing a custom Rhesus Macaque germline VDJ library (45, 46, 86) and adjusting the constants.py file for a maximum CDR3 length of 110 nucleotides. Gene expression as well as oligo-barcoded antibody hashtag and probe counts were obtained from gene expression and feature libraries using CellRanger v6, aligning to the Ensemble Mmul10 genome. Sequencing data were demultiplexed using the MULTIseqDemux command in Seurat v.4. For B cells with kappa and lambda light-chain contigs, lambda was assigned when both were present. The VDJ sequences were then parsed into an AIRR format using the Change-O pipeline from the Immcantation portal for comprehensive analysis.

### Bioinformatic analysis of sequences

#### Sequence Processing

The output from 10X VDJ contigs was re-annotated using the SADIE 0.4.31 library (https://github.com/jwillis0720/sadie) with the “macaque” option as the input annotation species. This resulted in a paired AIRR compliant dataframe (87). The dataframe was divided into IGH, IGL, and IGK assigned locus and paired based on 10X hashtag, provided they had exactly one heavy IGH and one IGL or IGK call. Somatic hypermutation was calculated by dividing the number of VH or VK/VL gene mutations by the total length of the VH or VK/VL gene. Inferred germline aa sequences were computed by reverting templated V, D, and J gene segments to their germline sequences, based on the predicted alleles from the antibody nucleotide sequence.

#### Apex bnAb-like Definition

The paired dataframe sequences were defined as Apex bnAb-like if they were >= 24 HCDR3 aa long, using IGHD3–15 with the regular expression “…….[DE][DE]Y…..…..“.

#### Apex bnAb-like (22 aa) Definition

The paired dataframe sequences were defined as Apex bnAb-like (22 aa) if they were >= 22 and < 24 HCDR3 aa long, using IGHD3–15 with the regular expression “…….[DE][DE]Y…..…..”.

Analysis of Apex bnAb-like BCR features was conducted using custom Python functions available in the data repository (https://github.com/schieflab/ma2025).

The scripts can be accessed at https://github.com/schieflab/ma2025

### Cell surface antigenic profiling

DNA-encoded membrane-bound trimers (Moderna) were transfected into HEK293F suspension cells grown in 293 Freestyle media (Life Technologies) by the 293fectin Transfection Reagent (Gibco) and incubated at 37°C, 125rpm for 2 days. Each antibody solution for antigenic profile test (in Fig. 5J) was prepared at 10 ug/mL in FACS buffer (HBSS, 1 mM EDTA, 1% BSA). To note, trimer-specific bnAbs (interface/FP: PGT151, Apex: PGT145, PG9 and PCT64.35S) and non-nAbs (V3: 4025, CD4bs: B6 and F105) were selected to characterize the open vs. closed nature of the membrane-bound trimer; not-trimer-specific bnAbs (N332: PGT121 and CD4bs: 12A12) were selected to evaluate cell surface immunogen expression; RMs mAbs (REt18_wk17_023, RJz18_wk12_041 and RIo18_wk8_018) were selected to assess binding capacity of ApexGT6 induced Apex bnAb-related precursor mAbs towards the immunogen and Apex epitopes closer to native. Cell suspensions were distributed into 96-well plates and analyzed as previously described (18).

### Apex bNAb precursor frequency estimates

Apex bnAb precursor searching and frequency estimates were performed as described previously (18), with modifications to JH gene usages of CH01–04. PySpark scripts used in this analysis are available at https://github.com/SchiefLab/Ma2025 along with instructions on setting up an EMR cluster.

### Neutralization assay

MAb neutralizing activity was assessed using single round of replication in TZM-bl target cells as previously described(22).

### Statistical analysis

Statistical analyses and graphing were carried out using GraphPad Prism for the experimental data. Wilcoxon matched-pairs signed rank test was used to compare before and after samples from the same animal, Mann-Whitney compare ranks test was used to compare two experimental groups, and one-way analysis of variance (ANOVA) with Dunn’s multiple comparison test was used for comparing more than two groups. *P < 0.05, **P < 0.01, ***P < 0.001, ns > 0.05. Details of the statistical test and number of replicates are indicated in the figure legends. A value of *P* < 0.05 was considered statistically significant.

## Supplementary Material

Supplementary Materials

Data files S3

Data files S2

Data files S4

MDAR Reproducibility Checklist

Data files S1

The Word file includes:

Figs. S1 to S13.

Tables S1 to S4.

Data files S1 to S4

## Figures and Tables

**Table 1.** Number of sorted and sequenced BCRs with specific binding and sequential features that were isolated from FNA samples of each ApexGT6 adjuvanted-protein immunized RM.

NHP id	ApexGT6^−^	ApexGT6^++^	ApexGT6^++^/ApexGT6 KO^−^	long HCDR3s among ApexGT6^++^/ApexGT6 KO^−^	Apex bnAb-related precursors among ApexGT6^++^/ApexGT6 KO^−^
RGp18	355	327	198	185	183
RJz18	2033	764	625	92	89
RIo18	1689	463	281	39	29
REt18	1890	506	336	11	9
RPb18	1411	295	248	4	2
RPz18	790	21	19	0	0
Total	8168	2376	1707	331	312

**Table 2.** Number of sorted and sequenced BCRs with specific binding and sequential features that were isolated from PBMC samples of each ApexGT6 adjuvanted-protein immunized RM.

NHP id	ApexGT6^++^	ApexGT6^++^/ApexGT6 KO^−^	long HCDR3s among ApexGT6^++^/ApexGT6 KO^−^	Apex bnAb-related precursors among ApexGT6^++^/ApexGT6 KO^−^
REt18	286	286	14	11
RPz18	153	153	12	7
RIo18	227	177	7	3
RPb18	2	2	0	0
RJz18	1	1	0	0
RGp18	9	9	0	0
Total	678	628	33	21

**Table 3.** Number of sorted and sequenced BCRs with specific binding and sequential features that were isolated from PBMC samples of each ApexGT6 membrane-bound mRNA-LNP immunized RM.

NHP id	ApexGT6^++^	ApexGT6^++^/ApexGT6 KO^−^	long HCDR3s among ApexGT6^++^/ApexGT6 KO^−^	Apex bnAb-related precursors among ApexGT6^++^/ApexGT6 KO^−^
RAu18	48	48	4	4
RGu18	43	43	10	10
RJr18	399	399	118	99
RPb19	83	83	14	11
RWj18	760	543	97	92
RYu18	68	68	2	2
Total	1401	1184	245	218

## Data Availability

Cryo-EM atomic models have been deposited in the Protein Data Bank with accession codes 9MQG, 9B8B and 9B8C (Table S2). All cryo-EM maps are available in the Electron Microscopy Data Bank (EMDB) under accession codes EMD-48523, EMD-44342, and EMD-44341 (Table S2). Crystallographic data have been deposited in the Protein Data Bank with accession codes 9MPC and 9MPB (Table S3). All BCR sequences (VDJ+Hashtag single cell data) have been deposited to the Gene Expression Omnibus (GEO304129). All bioinformatic analysis code from these studies are available in the GitHub public data repository (https://github.com/schieflab/ma2025) and permanently archived at Zenodo (https://doi.org/10.5281/zenodo.15588674). All other data are available in the main text or the supplementary materials. Plasmids or proteins related to the immunogens, sort reagents, or antibodies employed in this study are available from W.R.S. (schief@scripps.edu) under a material transfer agreement with The Scripps Research Institute. mRNA vaccine constructs may be made available from S.H. (Sunny.himansu@modernatx.com) if the recipient and Moderna are able to agree upon the terms of a material transfer agreement. Materials or information related to the SMNP adjuvant are available from D.J.I (djirvine@scripps.edu) under a material transfer agreement with MIT.
