# Supplementary material for: HIV broadly neutralizing antibody precursors to the Apex epitope induced in non-human primates
Source: Sci Immunol. Author manuscript; Available in PMC 2025 Sep 22. (PMC12453070; doi:10.1126/sciimmunol.adt6660)
Supplement: Supplementary Materials [file NIHMS2111339-supplement-Supplementary_Materials.pdf]

Science Immunology
AAAS

Supplementary Materials for

HIV broadly neutralizing antibody precursors to the Apex epitope induced in non-human primates

Krystal M. Ma, Henry J. Sutton, Payal P. Pratap, Jon M. Steichen, Diane Carnathan, James Quinn, Oleksandr Kalyuzhniy, Alessia Liguori, Sashank Agrawal, Sabyasachi Baboo, Patrick Madden, Christopher A. Cottrell, Jordan R. Willis, Jeong-Hyun Lee, Elise Landais, Xiaozhen Hu, Parham Ramezani-Rad, Gabriel Ozorowski, Vanessa R. Lewis, Jolene K. Diedrich, Xiaoya Zhou, Tasha K. Altheide, Nicole Phelps, Erik Georgeson, Nushin B. Alavi, Danny Lu, Saman Eskandarzadeh, Michael Kubitz, Yumiko Adachi, Tina-Marie Mullen, Murillo Silva, Mariane B. Melo, Sunny Himansu, Darrell J. Irvine, Dennis R. Burton, John R. Yates III, James C. Paulson, Devin Sok, Ian A. Wilson, Guido Silvestri, Andrew B. Ward, Shane Crotty, William R. Schief
Corresponding author: andrew@scripps.edu (A.B.W.), shane@lji.org (S.C.), or schief@scripps.edu (W.R.S.)

The PDF file includes:

Figs. S1 to S13
Tables S1 to S4

Other Supplementary Materials for this manuscript include the following:

Data files S1 to S4
MDAR Reproducibility Checklist

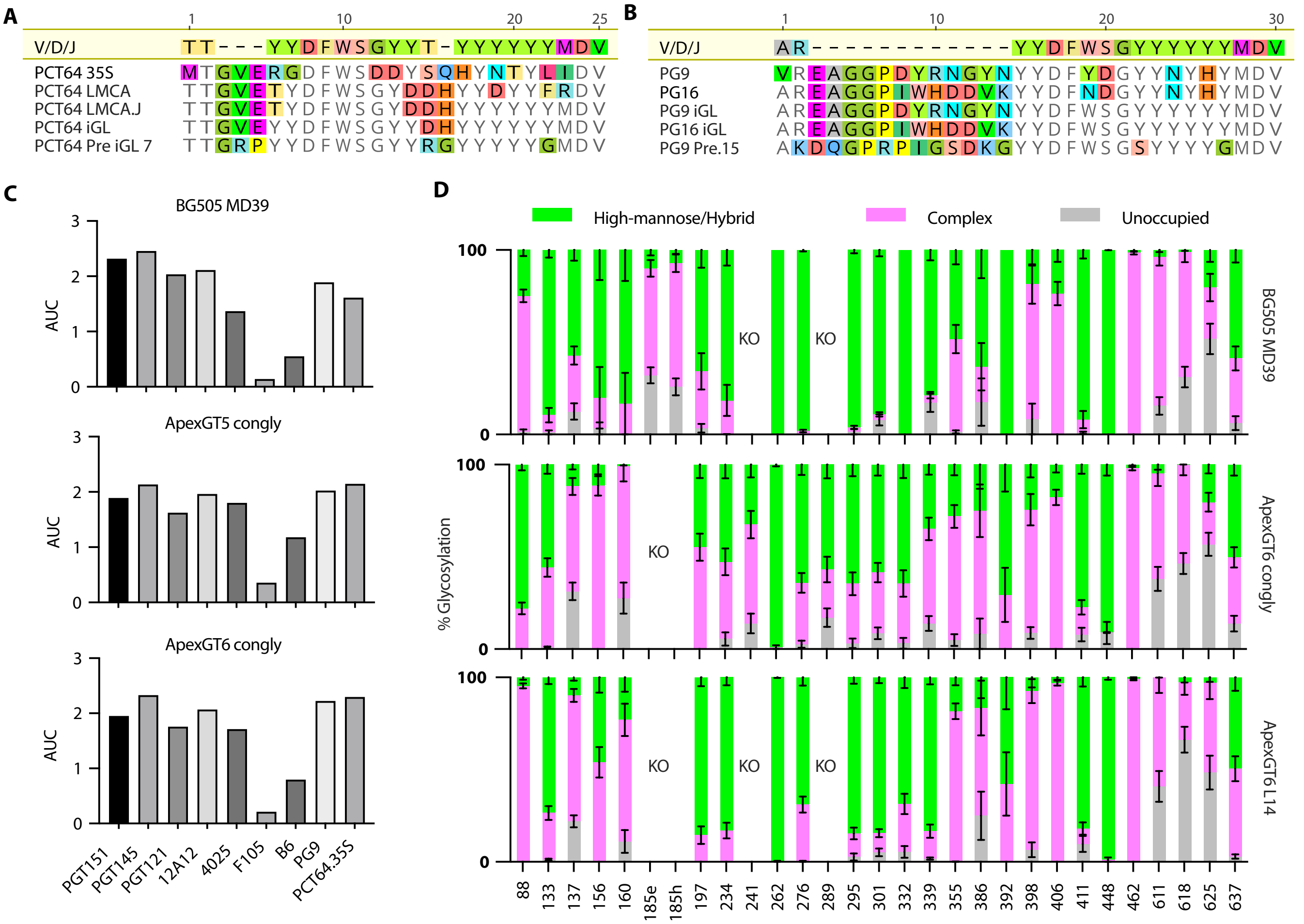

E

	Tm
ApexGT5	72.36
ApexGT5.congly	72.58
ApexGT6	71.48
ApexGT6.congly	70.91

F

Series	Repertoire	HCDR3	D gene	HCDR3 motif
Human PCT64	Human	≥ 24 aa	IGHD3-3	......[FI]W[ST]............
RM PCT64	RM	≥ 24 aa	IGHD3-41	......[FI]W[ST]............
Human DDY	Human	≥ 24 aa		.......[DE][DE]Y..........
RM DDY	RM	≥ 24 aa		.......[DE][DE]Y..........
Human PG9	Human	≥ 29 aa	IGHD3-3	...............[FI]W[ST]........
RM PG9	RM	≥ 29 aa	IGHD3-41	...............[FI]W[ST]........

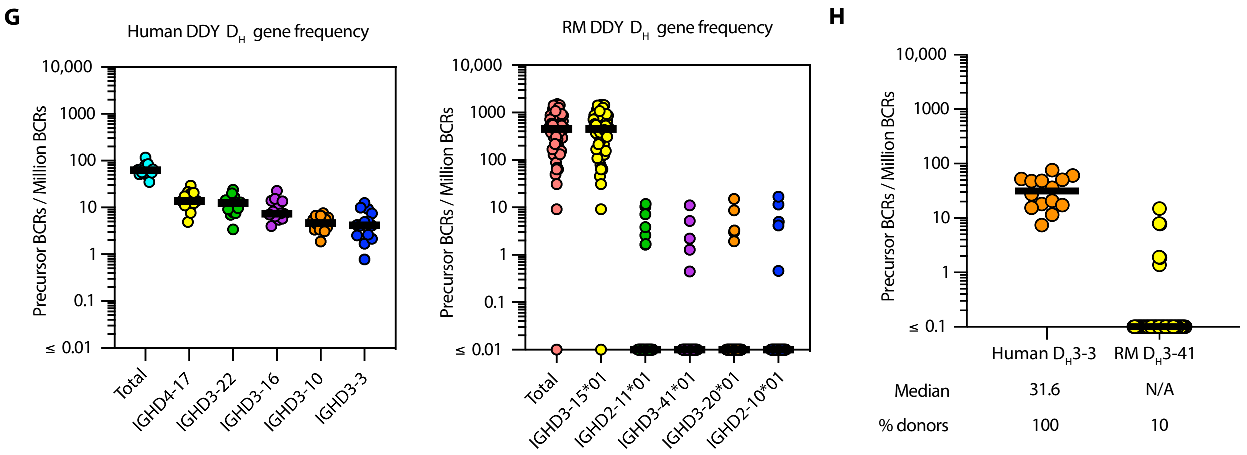

Fig. S1. Binding and stability evaluation of ApexGT trimers.
(A) HCDR3 aa sequence alignment of mAbs tested in Figure 1C. Mature bnAbs, least mutated ancestors (LMCA), inferred germlines (iGL), human NGS precursors (Pre), and corresponding V/D/J genes are also included for reference. (B) Antigenic profiles of MD39, ApexGT5, and ApexGT6 trimers. AUC is the area under the curve of the dilution series of the antibody shown on the x-axis. Closed-trimer binding bnAbs (PGT151, PGT145, PG9, and PCT64.35S), protomer-binding bnAbs (PGT121 and 12A12), and open-trimer binding non-nAbs (4025, B6, and F105) were selected to the antibody list to assess antigenic profile. (C) Melting temperature of ApexGT trimers as assessed by differential scanning calorimetry (DSC). (D) Glycosylation profiling for MD39 and ApexGT6 trimers was conducted using DeGlyPHER2. High-mannose and hybrid are shown in green, complex in pink, unoccupied in gray; KO represents glycans designed to be knocked out. ApexGT6 L14 refers to the soluble protein truncated at D664 (gp140) version of ApexGT6 L14 gp151. The result of MD39 was adapted with permission from Baboo et. al., Anal Chem. 2021, 93(40):13651-7 . Copyright 2021 American Chemical Society (E) The precursor definitions for each Apex series. The HCDR3 motif is used to define the aa sequence features of HCDR3. "." indicates a wildcard character used in the database search and will return all 20 aa at that position. The aa in between the brackets indicate the allowed mutations at that position (e.g. [FI] will return either Phenylalanine [F] or Isoleucine [I] at that position). A specific number of dots before and after the motif indicates that the HCDR3 sequences are at least a certain number of amino acids away from the beginning and the end of the motif. This positions the motif approximately in the middle of the HCDR3. (F) The D gene frequency of Apex bnAb-like HC sequences (with a DDY motif around the middle of the long HCDR3) in 14 human donors (left) and 60 RMs (right). Only the top five most frequent D genes from each search were plotted. The median was plotted across all donors. (G) The frequency of PG9-like HC sequences in 14 human donors and 60 RMs. Median was plotted across all donors. Source data can be found in Data file S4.

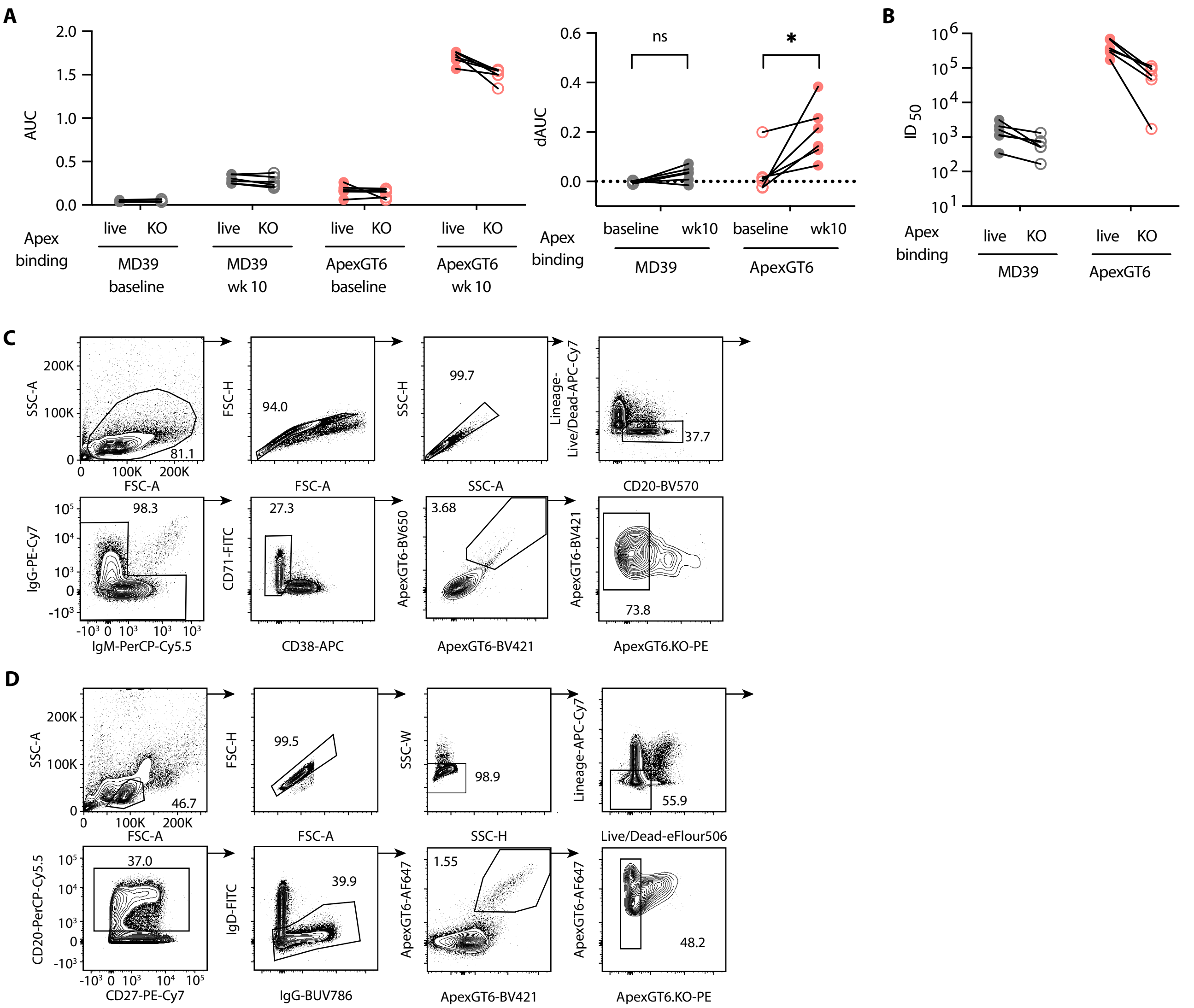

Fig. S2. ApexGT6 adjuvanted soluble protein elicited strong epitope-specific responses.
(A) ELISA quantification of serum IgG binding to antigen (labeled as live) and antigen with Apex-binding knock out (labeled as KO) from MD39 immunized macaques (grey dots, n=6) or ApexGT6 immunized macaques (red dots, n=6) prior to immunization (baseline) and at 10 weeks after first immunization (wk10). dAUC was calculated by AUC of antigen-binding subtracted by AUC of antigen-Apex.KO-binding. Wilcoxon matched-pairs signed rank test, n = 6. *$P < 0.05$, ns > 0.05. (B) ELISA quantification of serum IgG titers at week 10, showing binding to antigen (labeled "live") and antigen with Apex-binding knockout (labeled "KO") in macaques immunized with either MD39 (grey dots, n=6) or ApexGT6 (red dots, n=6). (C) Gating strategy used to identify epitope-specific B_{GC} cells. Live B_{GC} cells were identified as $CD20^+CD38^-$. Epitope-specificity was determined via binding to ApexGT6 BV650 and BV421 probes, but no binding to the ApexGT6.KO PE probe. $CD20^+CD38^-ApexGT6^{++}$ were sorted for BCR sequencing. (D) Gating strategy used to identify epitope-specific B_{mem} cells. Live B_{mem} cells were identified as $CD20^+IgD^-$. Epitope-specificity was determined via binding to ApexGT6 AF647 and BV421 probes, but no binding to the ApexGT6.KO PE probe. $CD20^+IgD^-$ $ApexGT6^{++}$ were sorted for BCR sequencing. Source data can be found in Data file S4.

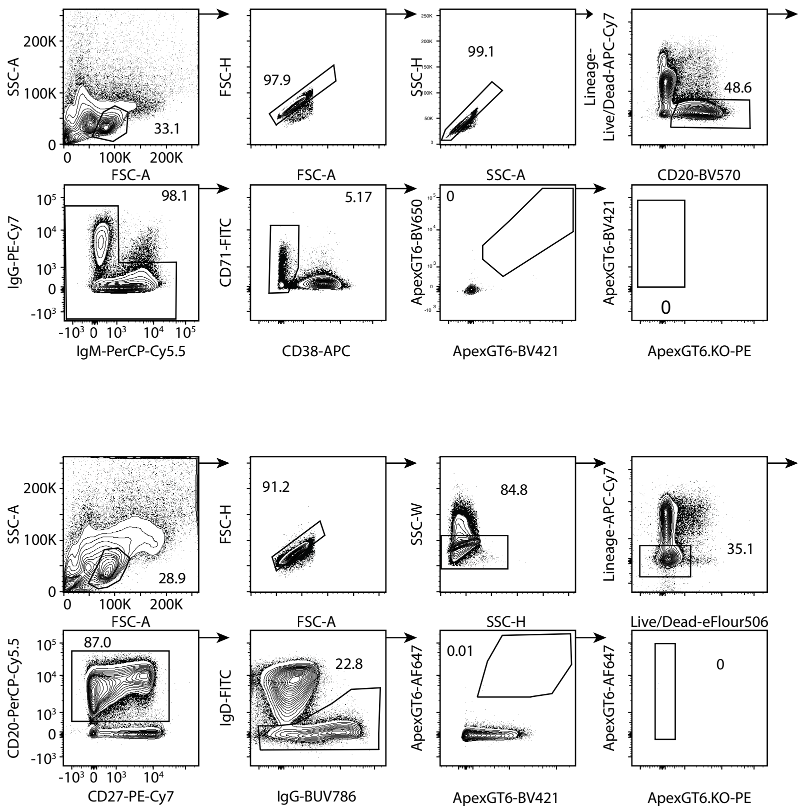

Fig. S3. Representative FACS plots of pre-immune fine needle aspirate (FNA, upper) and PBMC samples (lower). Baseline samples taken 2 weeks prior to the first immunization.

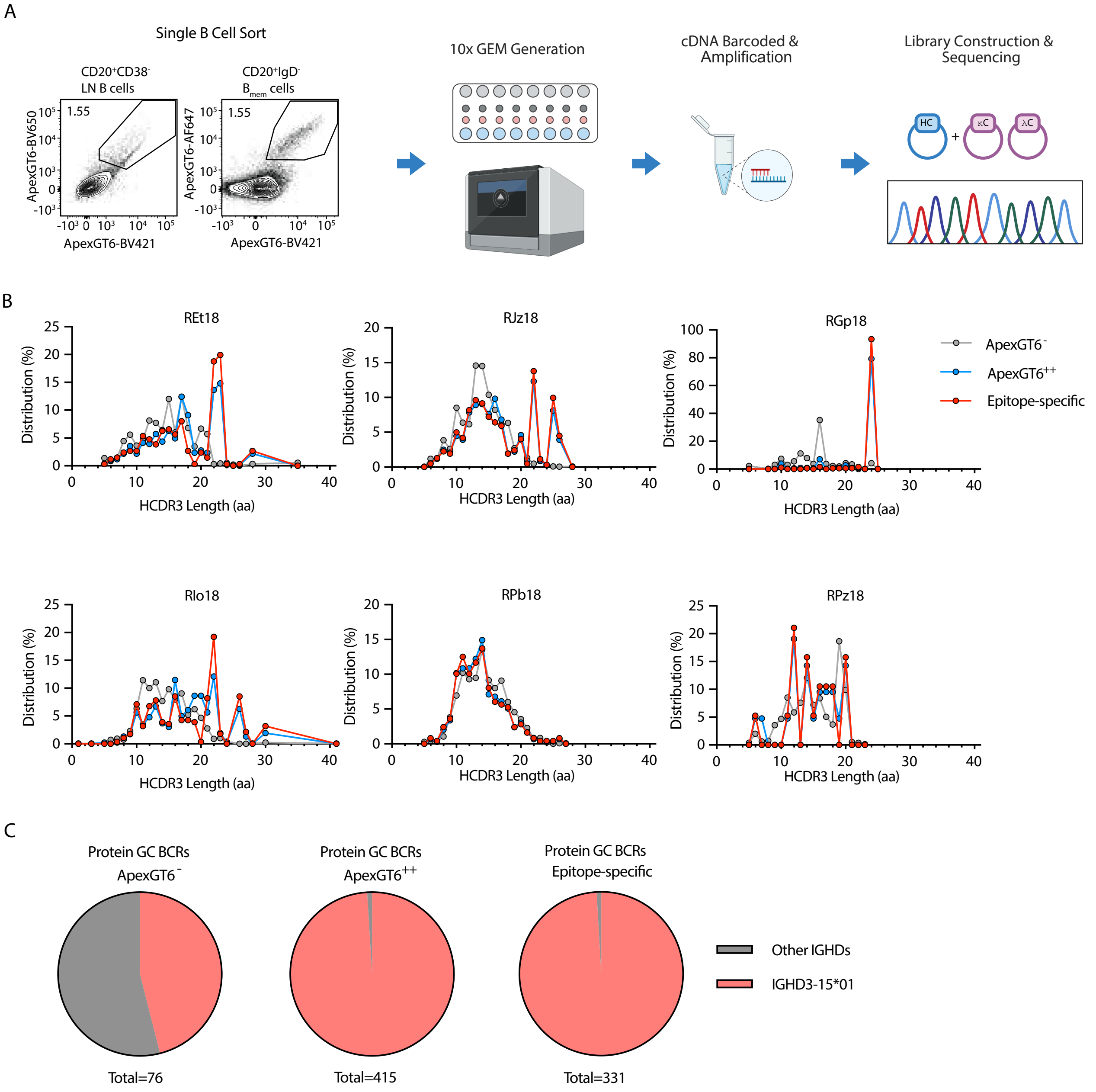

Fig. S4. ApexGT6 protein immunizations induced Apex bnAb-like precursor responses in all immunized animals. (A) Schematic for ApexGT6^{++} B cell sorting and 10x GEM sequencing. The example FACS data is copied from fig. S2D. **(B)** HCDR3 aa length distribution of BCRs in the lymph node for each RM. **(C)** Frequency comparison of RM IGHD3-15 (represented in red) among GC BCRs with long HCDR3s (≥ 24 aa) sorted and sequenced from ApexGT6 soluble protein immunized macaques. Left: ApexGT6- GC BCRs with long HCDR3s. Middle: ApexGT6^{++} GC BCRs with long HCDR3s. Right: epitope-specific GC BCRs with long HCDR3s. Source data can be found in Data file S4.

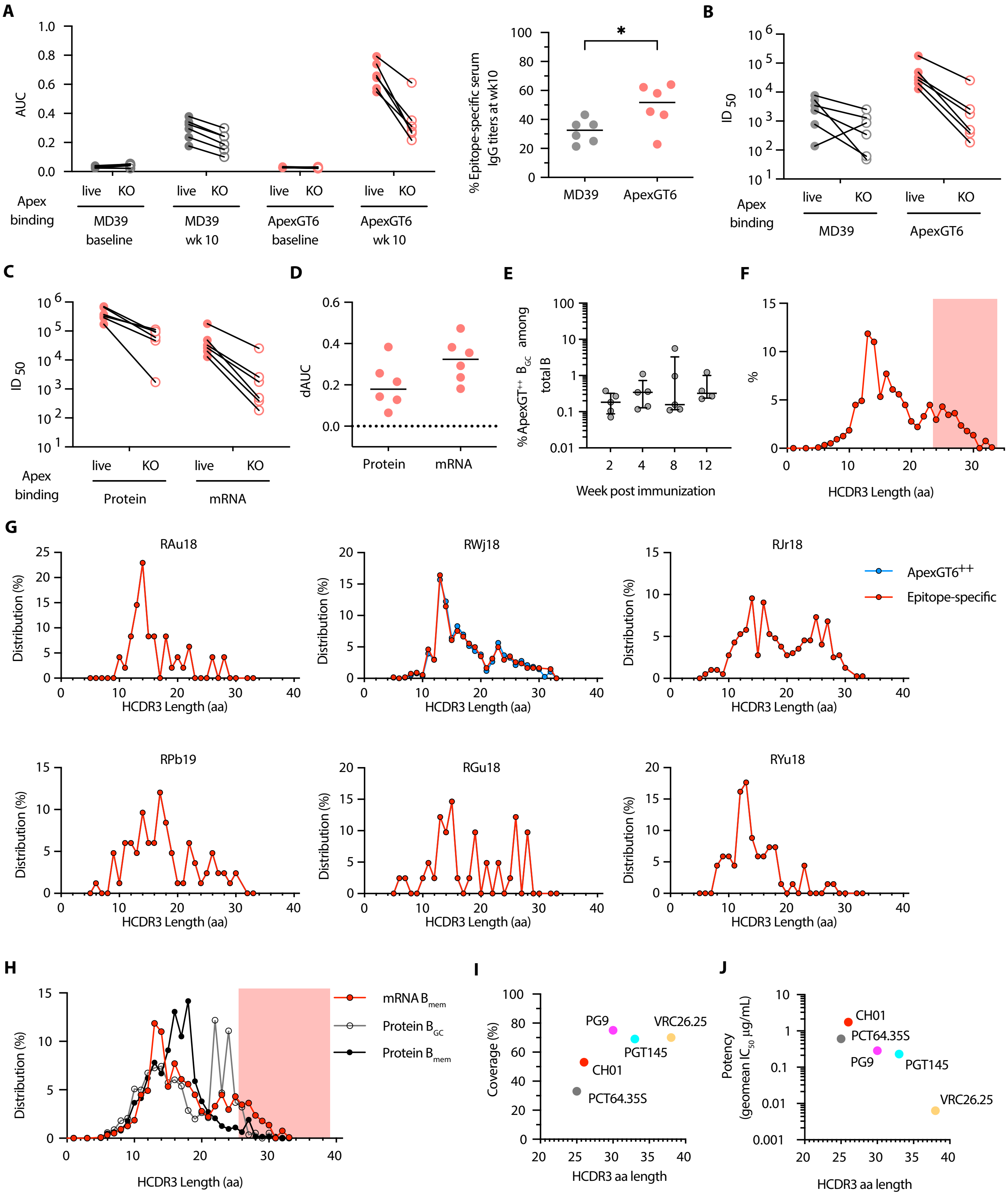
A
AUC
Apex binding
live
KO
MD39 baseline
MD39 wk 10
ApexGT6 baseline
ApexGT6 wk 10
% Epitope-specific serum IgG titers at wk10
*
MD39
ApexGT6
B
ID 50
C
D
dAUC
Protein
mRNA
E
% ApexGT++ B_GC among total B
Week post immunization
F
%
HCDR3 Length (aa)
G
RAu18
RWj18
RJr18
RPb19
RGu18
RYu18
Distribution (%)
ApexGT6++
Epitope-specific
H
mRNA B_mem
Protein B_GC
Protein B_mem
I
Coverage (%)
HCDR3 aa length
PG9
VRC26.25
PGT145
CH01
PCT64.35S
J
Potency (geomean IC50 µg/mL)

Fig. S5. Membrane-bound ApexGT6 mRNA-LNP induced robust Apex bnAb-like precursor memory responses in all immunized animals. (A) ELISA quantification of serum IgG titers binding to antigen (labeled as "live") and antigen with Apex-binding knockout (labeled as "KO") from MD39-immunized macaques (grey dots, n=6) or ApexGT6-immunized macaques (red dots, n=6) prior to immunization (baseline) and at 10 weeks after first immunization (wk10). dAUC was calculated by AUC of antigen binding subtracted by AUC of antigen-Apex.KO binding. Wilcoxon matched-pairs signed rank test, n = 6. *P < 0.05, ns > 0.05. Right: Percentages of antigen-binding serum IgG that are epitope-specific at week 10 in MD39 and ApexGT6 groups. Epitope-specific response was calculated by dAUC, which is the AUC of antigen binding minus the AUC of antigen-Apex.KO binding. Black lines indicate the median values. **(B)** ELISA quantification of serum IgG titers at week 10, showing binding to antigen (labeled "live") and antigen with Apex-binding knockout (labeled "KO") in macaques immunized with either MD39 (grey dots, n=6) or ApexGT6 (red dots, n=6). **(C)** ELISA quantification of serum IgG titers at week 10, showing binding to ApexGT6 (labeled "live") and ApexGT6 with Apex-binding knockout (labeled "KO") in macaques immunized with ApexGT6 either as protein or mRNA. **(D)** ELISA quantification of epitope-specific serum IgG binding from ApexGT6 protein or mRNA-immunized macaques at week 10. Black lines indicate the median values. **(E)** ApexGT6++ BGC cells as a percentage of total B cells post ApexGT6 mRNA-LNP immunization. Median and interquartile range are plotted depending on the scale in all figures unless otherwise stated. **(F)** HCDR3 aa length distribution of epitope-specific BCRs from the mRNA group in memory B cells. Data points ≥ 24 aa are highlighted with a red shadow. **(G)** HCDR3 aa length distribution of BCRs from the mRNA-LNP group in the memory B cells for each immunized macaque. **(H)** HCDR3 aa length distribution of epitope-specific BCRs from the mRNA group in memory BCRs (red), from the protein group in GC BCRs (grey) and memory BCRs (black). Data points ≥ 26 aa are highlighted with a red shadow. **(I)** Breadth of HCDR3-dependent Apex bnAbs, versus their HCDR3 length, from the Los Alamos database. **(J)** Potency of HCDR3-dependent Apex bnAbs, versus their HCDR3 length, from the Los Alamos database. Source data can be found in Data file S4.

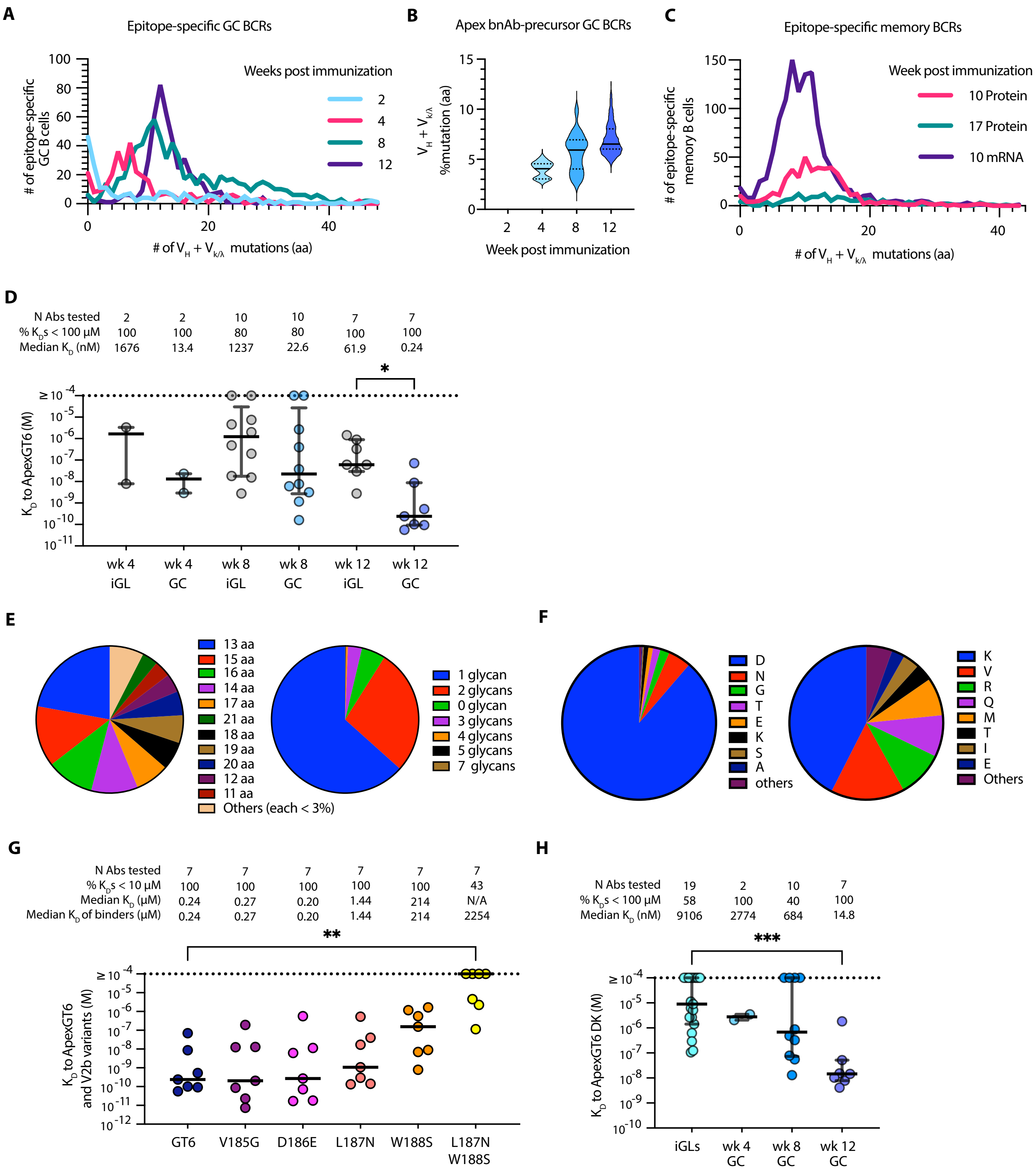
A
Epitope-specific GC BCRs
# of epitope-specific GC B cells
Weeks post immunization
2
4
8
12
# of V_H + V_k/λ mutations (aa)
B
Apex bnAb-precursor GC BCRs
V_H + V_k/λ %mutation (aa)
Week post immunization
C
Epitope-specific memory BCRs
# of epitope-specific memory B cells
Week post immunization
10 Protein
17 Protein
10 mRNA
# of V_H + V_k/λ mutations (aa)
D
N Abs tested 2 2 10 10 7 7
% K_Ds < 100 μM 100 100 80 80 100 100
Median K_D (nM) 1676 13.4 1237 22.6 61.9 0.24
K_D to ApexGT6 (M)
wk 4 iGL
wk 4 GC
wk 8 iGL
wk 8 GC
wk 12 iGL
wk 12 GC
E
13 aa
15 aa
16 aa
14 aa
17 aa
21 aa
18 aa
19 aa
20 aa
12 aa
11 aa
Others (each < 3%)
1 glycan
2 glycans
0 glycan
3 glycans
4 glycans
5 glycans
7 glycans
F
D
N
G
T
E
K
S
A
others
K
V
R
Q
M
T
I
E
Others
G
N Abs tested 7 7 7 7 7 7
% K_Ds < 10 μM 100 100 100 100 100 43
Median K_D (μM) 0.24 0.27 0.20 1.44 214 N/A
Median K_D of binders (μM) 0.24 0.27 0.20 1.44 214 2254
K_D to ApexGT6 and V2b variants (M)
GT6
V185G
D186E
L187N
W188S
L187N W188S
H
N Abs tested 19 2 10 7
% K_Ds < 100 μM 58 100 40 100
Median K_D (nM) 9106 2774 684 14.8
K_D to ApexGT6 DK (M)
iGLs
wk 4 GC
wk 8 GC
wk 12 GC

Fig. S6. Induced Apex bnAb-like precursor antibodies gained somatic hypermutation over time and acquired enhanced affinity for native-like Apex. (A) Frequency distributions of somatic hypermutation of epitope-specific GC BCRs at sampled timepoints. **(B)** Somatic hypermutation of Apex bnAb-like precursor GC BCRs at sampled timepoints. Somatic hypermutation is calculated by combining the number of mutations on VH and Vκ/λ, then dividing by their combined length. All calculations are based on aa. Thick lines indicate median values, and dash lines indicate 25 and 75% quantiles. **(C)** Frequency distributions of somatic hypermutation of epitope-specific memory BCRs at sampled timepoints. **(D)** SPR affinity measurement of mAbs derived from representative Apex bnAb-like precursor GC BCRs isolated at different timepoints compared with their inferred germlines (iGLs) binding to ApexGT6 and ApexGT6.KO. Lines indicate median values and 25 and 75% quantiles. Dunn's multiple comparisons test. $^*P < 0.05$. **(E)** Frequency of different V2b loop features on Envs across HIV strains from the Los Alamos database. Left: aa length of V2b loops (ranged 179 – 191 in HxB2 numbering). Right: numbers of glycans predicted according to aa sequence of V2b loops. **(F)** Frequency of different aa at position 167 (left) and 169 (right) on Envs across HIV strains from the Los Alamos database. **(G)** SPR affinity measurement of mAbs derived from representative Apex bnAb-like precursor GC BCRs isolated from protein group at week 12 binding to ApexGT6 and variants with germline-targeting mutations reverting to the ones from ApexGT2. Lines indicate median values. Dunn's multiple comparisons test. $^{**}P < 0.01$. **(H)** SPR affinity measurement of mAbs derived from representative Apex bnAb-like precursor GC BCRs isolated at different timepoints and their inferred germlines (iGLs) binding to ApexGT6-DK. Lines indicate median values and 25 and 75% quantiles. Dunn's multiple comparisons test. $^{***}P < 0.001$. To note, tested IgGs were co-transfected with Tyrosylprotein Sulfotransferase 1 (TPST1) to enhance tyrosine sulfation during production. Upper and lower lines represent the maximum and minimum values of binders, respectively. Box indicates 10-90 percentile with the median value of the binders. Source data can be found in Data file S4.

IC50 (ug/mL)	Starting at 50 ug/mL				
	RIo18_wk8_021	REt18_wk12_034	RGp18_wk12_035	RJz18_wk12_038	RJz18_wk12_041
BG505	NN	NN	NN	NN	NN
BG505_GT5.V2b	NN	NN	NN	NN	NN
BG505_GT5_R169K	NN	NN	NN	NN	NN
BG505_GT5_N167D	NN	NN	NN	2.4461	NN
BG505_GT5_DK	NN	NN	NN	NN	NN
HIV01428	NN	NN	NN	NN	NN
HIV01428_GT5_V2b	NN	NN	NN	NN	NN
AC_10	NN	NN	NN	NN	NN
AC10_GT5_V2b	NN	NN	NN	NN	NN
CNE8	NN	NN	NN	NN	NN
191084	NN	NN	NN	NN	NN
MLV	NN	NN	NN	NN	NN

IC50 (ug/mL)	Starting at 50 ug/mL					Starting at 5ug/mL			
	PCT64.LMCA	PCT64.13G	PCT64.18D	PCT64.24E	PCT64.35S	PG9	PGT145	RHA1	N6
BG505	NN	NN	0.1314	0.1430	0.1650	0.0336	0.0176	0.0672	0.0302
BG505_GT5.V2b	NN	0.0047	0.0033	0.0122	0.0207	0.0147	NN	0.0029	0.0500
BG505_GT5_R169K	NN	NN	0.3196	3.4048	4.7930	NN	NN	NN	0.2390
BG505_GT5_N167D	NN	NN	0.6730	12.6785	NN	0.1754	NN	0.3984	0.0986
BG505_GT5_DK	NN	NN	3.2677	20.8737	NN	0.4407	NN	NN	0.0869
HIV01428	NN	NN	NN	NN	NN	0.0216	0.1486	1.2542	0.0063
HIV01428_GT5_V2b	NN	NN	0.0275	0.1365	NN	0.0210	NN	0.0157	0.1049
AC_10	NN	NN	NN	NN	0.5962	0.1384	0.0231	NN	0.4245
AC10_GT5_V2b	NN	3.3171	0.0501	0.4118	NN	0.0424	1.6045	0.0162	0.4668
CNE8	NN	NN	NN	NN	NN	1.7489	0.3312	NN	0.2225
191084	NN	NN	8.7534	2.7478	NN	0.0446	0.2423	0.0274	0.0349
MLV	NN	NN	NN	NN	NN	NN	NN	NN	NN

Fig. S7. Neutralization assay of selected post-priming RM mAbs. We evaluated the RM mAbs elicited by ApexGT6, along with PCT64 lineage members and other V2 apex bnAbs. BG505_GT5_V2b PSV represents ApexGT5 with its V2b loop reverted to BG505 WT. HIV01428_GT5_V2b and AC10_GT5_V2b PSV contain the GT mutations within the Apex of ApexGT5, transferred to HIV01428 or AC10, with their V2b loops reverted to their respective WT sequences. All experiments were carried out in duplicate.

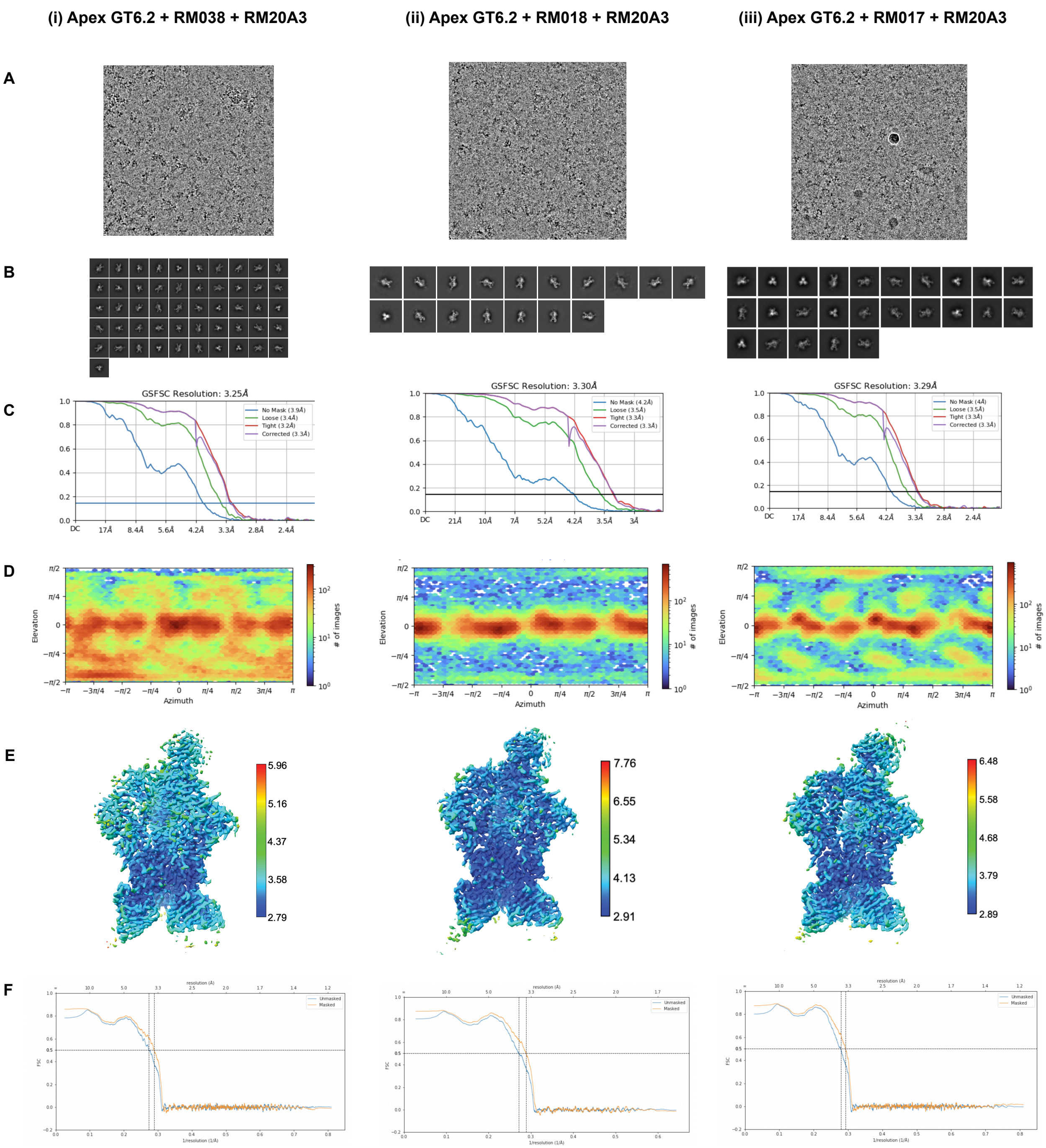

Fig. S8. Supplemental CryoEM Statistics. (A) Representative EM micrographs of (i) RM038 (ii) RM018 and (iii) RM017. **(B)** Representative 2D Classes of (i) RM038 (ii) RM018 and (iii) RM017. **(C)** Fourier shell correlation resolution estimates for (i) RM038 (ii) RM018 and (iii) RM017. **(D)** Euler distribution plot for (i) RM038 (ii) RM018 and (iii) RM017. **(E)** Local resolution estimates (in units Å) for (i) RM038 (ii) RM018 and (iii) RM017. **(F)** Map to atomic model fourier shell correlation for (i) RM038 (ii) RM018 and (iii) RM017.

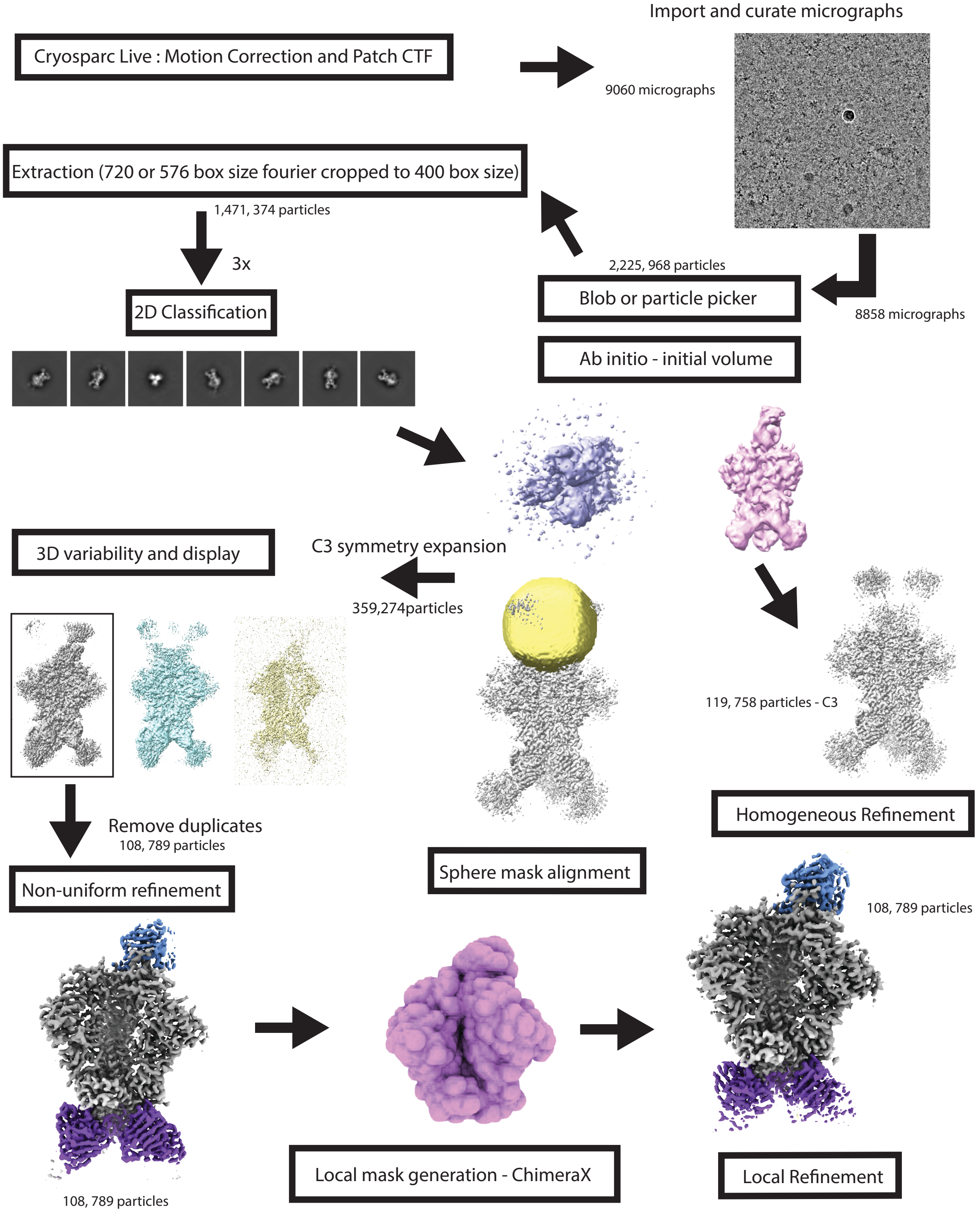

Fig. S9. Representative workflow for cryoEM processing and map generation and refinement.

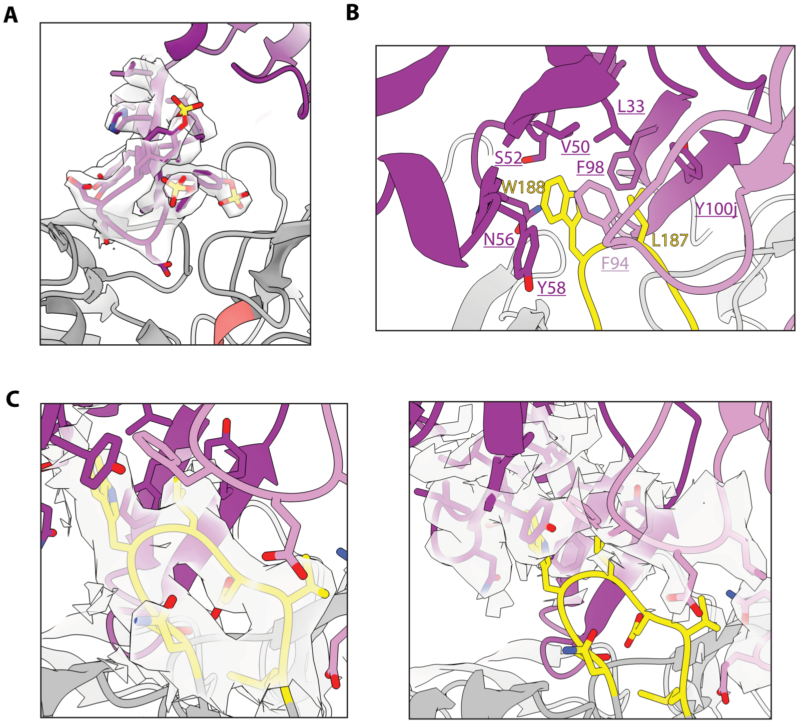

Fig. S10. ApexGT6 induced mAbs possessed structural features similar to PCT64. (A) RM017 HCDR3 with experimental map overlay in same view as Fig. 6E. **(B)** Detailed view of the hydrophobic pocket residues (shown as sticks) interacting with the engineered hydrophobic V2b loop (shown in yellow cartoon, with hydrophobic mutations as sticks). **(C)** RM017 interacting with V2b loop with experimental map overlap of the V2b loop (left) and the paratope (right) in zoomed in view of Fig. 6H.

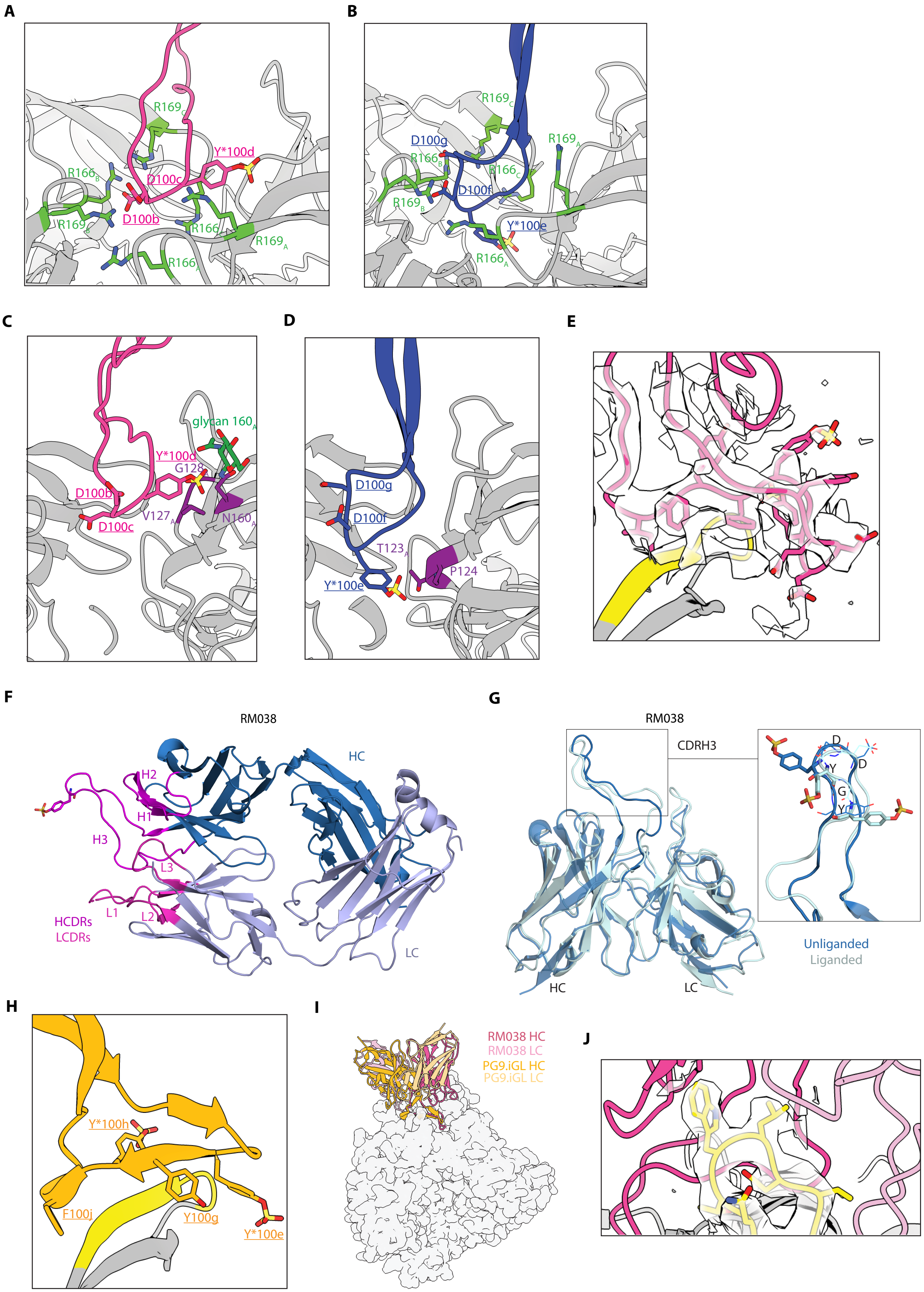
A
R169C
Y*100d
R166B
D100c
R169B
D100b
R166
R169A
R166A
B
R169C
D100g
R169A
R166B
R166C
D100f
R169B
Y*100e
R166A
C
glycan 160A
Y*100d
G128
D100b
V127A
N160A
D100c
D
D100g
D100f
T123A
Y*100e
P124
E
F
RM038
HC
H2
H1
H3
L3
L1
L2
HCDRs
LCDRs
LC
G
RM038
CDRH3
D
D
Y
G
Y
Unliganded
Liganded
HC
LC
H
Y*100h
F100j
Y100g
Y*100e
I
RM038 HC
RM038 LC
PG9.iGL HC
PG9.iGL LC
J

Fig. S11. ApexGT6 induced mAbs possessed structural features similar to PCT64 and PG9.
(A) Zoomed-in views of the DDY motif of RM038 (fuchsia sticks) interacting to the 3-fold axis of ApexGT6 (grey cartoon). Arginine 166 and 169 on the strand C depicted as green sticks. **(B)** Zoomed-in views of the DDY motif of PCT64 LMCA (blue sticks) interacting to the 3-fold axis of ApexGT2.2MUT (grey cartoon). Arginine 166 and 169 on the strand C depicted as green sticks. **(C)** Zoomed-in views of residues (purple sticks) on the3-fold axis of ApexGT6 (grey cartoon) within a 4Å interface to the sulfated tyrosine on the DDY motif (fuchsia sticks) of RM038. **(D)** Zoomed-in views of residues (purple sticks) on the 3-fold axis of ApexGT2.2MUT (grey cartoon) within a 4Å interface to the sulfated tyrosine on the DDY motif (fuchsia sticks) of PCT64 LMCA. **(E)** RM038 HCDR3 with experimental map overlap in same view as Fig. 7E. **(F)** Crystal structures of unliganded Fabs of RM038 shown in cartoon representation, with CDRs highlighted in magenta. **(G)** Structural alignment of the Fv regions of unliganded and ApexGT6-trimer-bound RM038 Fabs. The inset highlights the HCDR3 region, showing conformational changes and residues in the 'DDYXY' region. These residues are displayed as lines, with the sulfated tyrosine shown as sticks. **(H)** Side view of the HCDR3 of PG9 iGL (orange cartoon) and strand C (166-170 residues colored as yellow) on gp120A of ApexGT3, with key residues depicted as sticks. **(I)** The structure of RM038 (heavy chain: fuchsia, light chain: pink) in complex with ApexGT6 (white surface), aligned to the structure of PG9 iGL (heavy chain: orange, light chain: yellow) binding to ApexGT3 (white surface), but with flipped heavy and light chain. Two liganded structures were aligned using gp120A of each ApexGT trimer. **(J)** RM038 interacting with V2b loop with experimental map overlap of the V2b loop in zoomed in view of Fig. 7F.

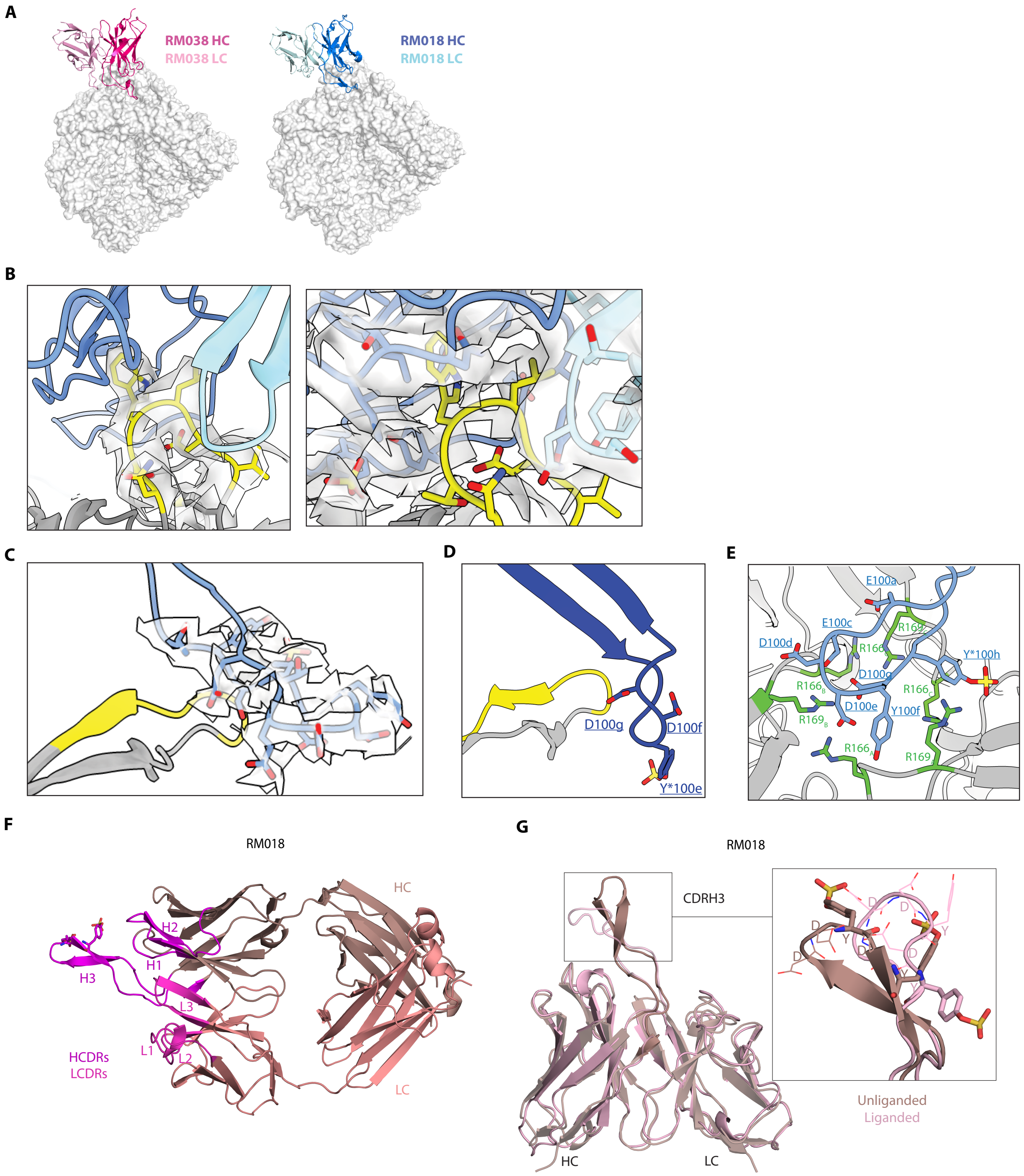

Fig. S12. ApexGT6 induced mAbs possessed structural features similar to CH01-04.
(A) Side views of the liganded structures of RM038 (pink) and RM018 (blue) by aligning the gp120A of each ApexGT6 trimer (white surface). **(B)** RM018 interacting with V2b loop with experimental map overlap of the V2b loop (left) and the paratope (right) in same view of Fig. 8B. **(C)** RM018 HCDR3 with experimental map overlap in same view as Fig. 8C. **(D)** Side view of the HCDR3 of PCT64 LMCA (navy blue) and strand C (166-170 residues colored as yellow) on gp120A of ApexGT6, with DDY motif depicted as sticks. **(E)** Zoomed-in views of the DDY motif of RM018 (blue sticks) interacting to the 3-fold axis of ApexGT6 (grey cartoon). Arginine 166 and 169 on the strand C depicted as green sticks. **(F)** Crystal structures of unliganded Fabs of RM018 shown in cartoon representation, with CDRs highlighted in magenta. **(G)** Structural alignment of the Fv regions of unliganded and ApexGT6-trimer-bound RM038 Fabs. The inset highlights the HCDR3 region, showing conformational changes and residues in the 'DDYXY' region. These residues are displayed as lines, with the sulfated tyrosine shown as sticks.

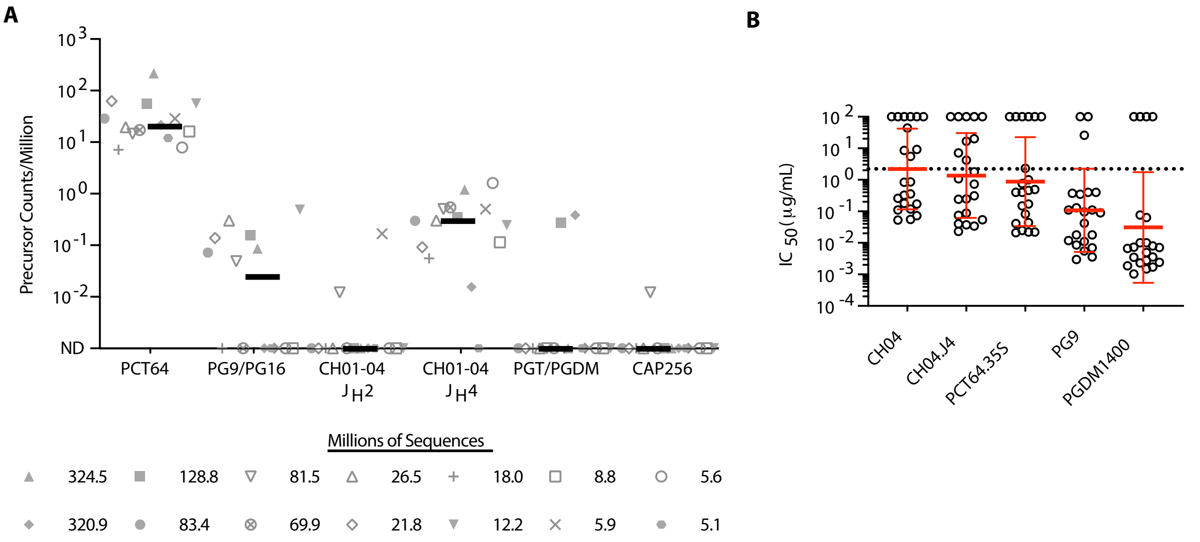

C

IC50 (µg/mL)	CH04	CH04.JH4	PCT64.35S	PG9	PGDM1400
TRO11	0.36	0.040	0.082	0.11	0.077
25710	0.85	0.24	0.025	0.018	0.0024
398F1	0.17	0.25	NN	26	NN
CNE8	NN	NN	1.0	0.091	0.0023
X2278	0.055	0.038	2.3	0.0056	0.0080
BJOX002000	5.6	1.8	0.15	0.042	0.0067
X1632	44	20	0.50	0.11	0.0072
CE1176	0.053	0.034	NN	0.0070	0.0074
246F3	0.33	1.08	0.022	0.011	0.0017
CH119	0.87	0.74	0.4	0.4	0.064
CE0217	0.12	0.075	0.46	0.003	0.0015
CNE55	NN	16.8	0.4	0.35	0.0050
92RW020	NN	NN	NN	0.40	0.010
94UG103	8.6	7.2	0.76	0.38	0.010
BGN	0.18	0.054	0.044	0.018	0.0028
92BR020	NN	NN	NN	NN	NN
JRCSF	0.26	0.31	0.022	0.0036	0.0036
JRFL	NN	NN	NN	NN	NN
WITO	0.073	0.023	0.0211	0.012	0.0035
IAVIC22	NN	NN	NN	0.096	NN
93IN905	NT	NT	NT	NT	NT
16055	9.3	4.2	0.17	0.0086	0.00104
92TH021	0.11	0.089	0.041	0.13	0.0019
MLV	NN	NN	NN	NN	NN
Geomean of detected	0.53	0.39	0.15	0.06	0.01
% detected	70	74	70	87	78

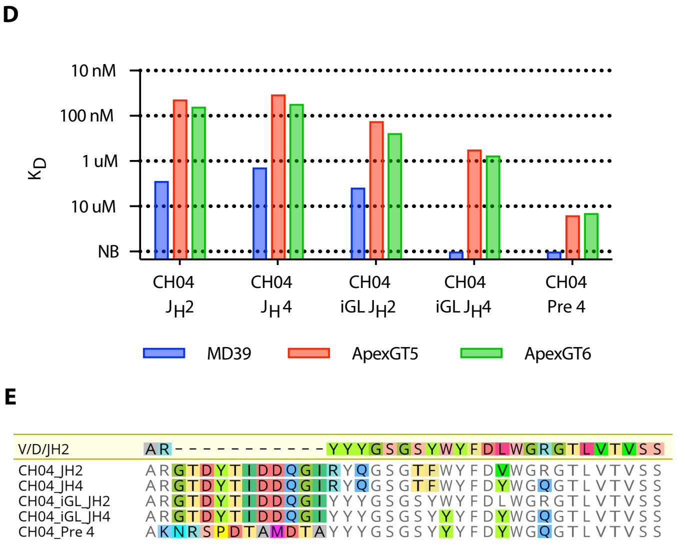

Fig. S13. JH4-variant CH01-04 bnAbs are an important vaccine target and ApexGT6 has potential to prime precursors for this class of bnAb. (A) Precursor frequencies for each donor, and for each class of HCDR3-dominant Apex bnAb, considering HC only. The number of unique sequences used in the precursor search for each donor is indicated. Black lines indicate median precursor frequencies computed over all values. **(B)** Neutralization assay of selected Apex bnAbs over a panel of 23 pseudovirus. Lines indicate geometric mean values and geometric standard deviations if IC50 (µg/mL). All experiments were carried out in duplicate. **(C)** IC50 (µg/mL) results in fig. S13B. "NN" indicates that no neutralization was detected, while "NT" indicated that no titer was detected. All experiments were carried out in duplicate. **(D)** SPR K_Ds for MD39 and ApexGT trimer analytes binding to CH04 variants as IgG ligands. The data were fit using a 1:2 binding model. NB, no binding, is the highest concentration (~ 10 µM) tested. **(E)** HCDR3 aa sequence alignment of mAbs tested in fig. S13D. Mature and inferred germlines (iGL) using either JH2 or JH4, human NGS precursors (Pre), and corresponding V/D/J genes are also included for reference. Source data can be found in Data file S4.

Table S1. Amino acid sequences of MD39 and ApexGT variants.
Bold-and-underline refers to ApexGT mutations, bold-only refers to the congly design (glycan hole filled), underline-only refers to L14, and italics refer to the transmembrane domain of the gp151 platform.

BG505 MD39 soluble trimer	AENLWVTVYYGVPVWKDAETTLFCASDAKAYETEKHNVWATHACVPTDPNPQEIHLENVTEEFNMWKNNMVEQMHEDIIS<br>LWDQSLKPCVKLTPLCVTLQCTNVTNNITDDMRGELKNCSFNMTTELRDKKQKVYSLFYRLDVVQINENQGNRSNNSNKE<br>YRLINCNTSAITQACPKVSFEPIPIHYCAPAGFAILKCKDKKFNGTGPCPSVSTVQCTHGIKPVVSTQLLLNGSLAEEEV<br>IIRSENITNNAKNILVQLNTPVQINCTRPNNNTVKSIRIGPGQAFYYTGDIIGDIRQAHCNVSKATWNETLGKVVKQLRK<br>HFGNNTIIRFAQSSGGDLEVTTHSFNCGGEFFYCNTSGLFNSTWISNTSVQGSNSTGSNDSITLPCRIKQIINMWQRIGQ<br>AMYAPPIQGVIRCVSNITGLILTRDGGSTNSTTETFRPGGGDMRDNWRSELYKYKVVKIEPLGVAPTRCKRRVVGRRRRR<br>R--------AVGIGAVSLGFLGAAGSTMGAASMTLTVQARNLLSGIVQQQSNLLRAPEPQQHLLKDTHWGIKQLQARVLA<br>VEHYLRDQQLLGIWGCSGKLICCTNVPWNSSWSNRNLSEIWDNMTWLQWDKEISNYTQIIYGLLEESQNQQEKNEQDLLA<br>LD**
ApexGT5 congly soluble trimer	AENLWVTVYYGVPVWKDAETTLFCASDAKAYETEKHNVWATHACVPTDPNPQEIHLENVTEEFNMWKNNMVEQMHEDIIS<br>LWDQSLKPCVKLTPLCVTLQCTNVTNNITDDMRGELKNCSFN**A**TTELR**N**K**R**QKVYSLFYRLD**I**V**PMVDLWTN--------**<br>YRLINCNTSAITQACPKVSFEPIPIHYCAPAGFAILKCKDKKFNGTGPC**QN**VSTVQCTHGIKPVVSTQLLLNGSLAEEEV<br>IIRSENITNNAKNILVQLNT**S**VQINCTRPNNNTVKSIRIGPGQAFYYTGDIIGDIRQAHCNVSKATWNETLGKVVKQLRK<br>HFGNNTIIRFAQSSGGDLEVTTHSFNCGGEFFYCNTSGLFNSTWISNTSVQGSNSTGSNDSITLPCRIKQIINMWQRIGQ<br>AMYAPPIQGVIRCVSNITGLILTRDGGSTNSTTETFRPGGGDMRDNWRSELYKYKVVKIEPLGVAPTRCKRRVVGRRRRR<br>R--------AVGIGAVSLGFLGAAGSTMGAASMTLTVQARNLLSGIVQQQSNLLRAPEPQQHLLKDTHWGIKQLQARVLA<br>VEHYLRDQQLLGIWGCSGKLICCTNVPWNSSWSNRNLSEIWDNMTWLQWDKEISNYTQIIYGLLEESQNQQEKNEQDLLA<br>LD**
ApexGT6 congly soluble trimer	AENLWVTVYYGVPVWKDAETTLFCASDAKAYETEKHNVWATHACV**S**TDPNPQEIHLENVTEEFNMWKNNMVEQMHEDIIS<br>LWDQSLKPCVKLTPLCV**G**LQCTNVTNNITDDMRGELKNCSFN**A**TTELR**N**K**R**QKVYSLFYRLD**I**V**PMVDLWTN--------**<br>YRLI**S**CNTSAITQACPKVSFEPIPIHYCAPAGFAILKCKDKKFNGTGPC**QN**VSTVQCTHGIKPVVSTQLLLNGSLAEEEV<br>IIRSENITNNAKNILVQLNT**S**VQINCTRPNNNTVKSIRIGPGQAFYYTGDIIGDIRQAHCNVSKATWNETLGKVVKQLRK<br>HFGNNTIIRFAQSSGGDLEVTTHSFNCGGEFFYCNTSGLFNSTWISNTSVQGSNSTGSNDSITLPCRIKQIINMWQRIGQ<br>AMYAPPIQGVIRCVSNITGLILTRDGGSTNSTTETFRPGGGDMRDNWRSELYKYKVVKIEPLGVAPTRCKRRVVGRRRRR<br>R--------AVGIGAVSLGFLGAAGSTMGAASMTLTVQARNLLSGIVQQQSNLLRAPEPQQHLLKDTHWGIKQLQARVLA<br>VEHYLRDQQLLGIWGCSGKLICCTNVPWNSSWSNRNLSEIWDNMTWLQWDKEISNYTQIIYGLLEESQNQQEKNEQDLLA<br>LD**
ApexGT6 L14 gp151 membrane-anchored trimer	AENLWVTVYYGVPVWKDAETTLFCASDAKAYETEKHNVWATHACV**S**TDPNPQEIHLENVTEEFNMWKNNMVEQMHEDIIS<br>LWDQSLKPCVKLTPLCV**G**LQCTNVTNNITDDMRGELKNCSFN**A**TTELR**N**K**R**QKVYSLFYRLD**I**V**PMVDLWTN--------**<br>YRLI**S**CNTSAITQACPKVSFEPIPIHYCAPAGFAILKCKDKKFNGTGPCPSVSTVQCTHGIKPVVSTQLLLNGSLAEEEV<br>IIRSENITNNAKNILVQLNTPVQINCTRPNNNTVKSIRIGPGQAFYYTGDIIGDIRQAHCNVSKATWNETLGKVVKQLRK<br>HFGNNTIIRFAQSSGGDLEVTTHSFNCGGEFFYCNTSGLFNSTWISNTSVQGSNSTGSNDSITLPCRIKQIINMWQRIGQ<br>AMYAPPIQGVIRCVSNITGLILTRDGGSTNSTTETFRPGGGDMRDNWRSELYKYKVVKIEPLGVAPTRCKRRVVGSHSGS<br>GGSGSGGHAAVGIGAVSLGFLGAAGSTMGAASMTLTVQARNLLSGIVQQQSNLLRAPEPQQHLLKDTHWGIKQLQARVLA<br>VEHYLRDQQLLGIWGCSGKLICCTNVPWNSSWSNRNLSEIWDNMTWLQWDKEISNYTQIIYGLLEESQNQQEKNEQDLLA<br>LD*KWASLWNWFDISNWLWYIKIFIMIVGGLIGLRIVFAVLSVIHRVR***

Table S2. Cryo-EM data collection, refinement and validation statistics

	RM038 Fab in Complex with Apex GT 6.2 and RM20A3 (PDB 9B8B) (EMD-44341)	RM018 Fab in Complex with Apex GT 6.2 and RM20A3 (PDB 9B8C) (EMD-44342)	RM017 Fab in Complex with Apex GT 6.2 and RM20A3 (PDB 9MQG) (EMD-48523)
**Data collection and processing**			
Microscope	TFS Glacios	TFS Glacios	TFS Glacios
Voltage (keV)	200	200	200
Camera	TFS Falcon 4i	TFS Falcon 4i	TFS Falcon 4i
Magnification	190,000x	190,000x	190,000x
Pixel size at detector (Å)	0.725	0.725	0.725
Total electron exposure (e–/Å^2)	45	45	45
Exposure rate (e-/pixel/sec)	6.619	6.045	6.661
Number of EER frames	40	40	40
Defocus range (μm)	0.8 to -1.8	0.8 to -1.8	0.8 to -1.8
Automation software	EPU	EPU	EPU
Micrographs collected (no.)	10955	9060	9085
Micrographs used (no.)	10573	8858	8564
Total extracted particle images (no.)	131,058	108789	140,520
Symmetry	C1	C1	C1
Map resolution (masked/unmasked Å)	3.3/4	3.3/3.3	3.3/3.5
FSC threshold	0.143	0.143	0.143
Map sharpening *B* factor (Å^2)	-78.7	-67.0	-79.4
Map resolution range (Å)	2.9-6.5	2.9-8.0	2.8-6.0
**Refinement**			
Initial model used (PDB code)	7T73	5IL8, 7TP4, 5N4J	7TP4, 7E9O, 5VZY
Refinement package	Phenix real space refine	Phenix real space refine	Phenix real space refine
Model resolution (Å)	3.4	3.5	3.4
FSC threshold	0.5	0.5	0.5
EMRinger score	3.24	3.06	3.68
CC (mask)	0.77	0.82	0.84
Model composition			
Non-hydrogen atoms	21,008	20,900	21,332
Protein residues	2,568	2,556	2,618
Ligands	74	76	68
Mean B factors (Å^2)			
Protein	75.76	76.86	73.38
Ligand	77.25	103.94	103.81
R.m.s. deviations			
Bond lengths (Å)	0.005	0.006	0.005
Bond angles (°)	0.854	1.23	0.990
Validation			
MolProbity score	0.90	1.09	0.80
Clashscore	0.94	1.19	0.71
Poor rotamers (%)	0.05	0.18	0.04
Ramachandran plot			
Favored (%)	97.41	96.25	97.65
Allowed (%)	2.59	3.75	2.35
Disallowed (%)	0.00	0.00	0.00
Cβ outliers (%)	0.00	0.00	0.00
CaBLAM outliers (%)	2.60	3.12	2.63

Table S3. X-ray data collection and refinement statistics

Data Collection	RM018	RM014	RM038
Beamline	NSLS-II 17-ID-1	SSRL BL12-2	SSRL BL12-2
Wavelength (Å)	0.920	0.980	0.980
Resolution (Å)	30.00 - 3.30 (3.36 – 3.30)	30.00 - 1.88 (1.93 - 1.88)	50.00 - 1.98 (2.01 - 1.98)
Space group	I 4 2 2	C 1 2 1	C 1 2 1
Unit cell a, b, c (Å)	118.7 118.7 194.4	98.8 60.3 82.0	181.2 66.5 53.4
α, β, γ (°)	90 90 90	90 112.9 90	90 103.2 90
Total reflections	167,184	196,145	239,320
Unique reflections	10,810	35,396	40,445
Multiplicity	15.5 (16.4)	5.6 (3.1)	5.9 (6.4)
Completeness (%)	100.0 (100.0)	98.4 (96.9)	94.1 (94.6)
Mean I/sigma(I)	7.6 (1.1)	11.6 (1.1)	28.7 (6.6)
Rsym	0.28 (1.38)	0.19 (0.81)	0.09 (0.48)
Rpim	0.07 (0.34)	0.08 (0.52)	0.04 (0.21)
CC1/2	0.99 (0.62)	0.99 (0.52)	0.99 (0.92)
**Refinement Statistics**			
Resolution (Å)	29.71 - 3.30	28.90 - 1.88	41.58 - 1.98
Reflections total / Rfree	10,668 / 1270	35,317 / 2011	40,422 / 2800
Rcryst / Rfreee	0.25 / 0.31	0.19 / 0.24	0.19 / 0.22
No. of copies in ASU	1	1	1
Number of atoms	3299	3342	3549
macromolecules	3281	3200	3384
ligands	18	-	-
solvent	-	142	165
Average B-factor ($Å^2$)	72	28	31
macromolecules	72	28	31
solvent	-	27	33
Wilson B-factor ($Å^2$)	84	22	26
**RMSD from ideal geometry**			
Bond angle (°)	0.77	0.79	1.43
Bond length (Å)	0.005	0.006	0.017
**Ramachandran statistics (%)**			
Favored	94.6	97.4	97.9
Allowed	5.4	2.4	1.62
**PDB Code**	**9MPC**	**9MPX**	**9MPB**

Statistics for the highest-resolution shell are shown in parentheses.

Table S4. Commercial antibodies used in this study.

Antibody	Source	Identifier (cat# and RRID)	
Goat Anti-Human IgG Fc-UNLB	SouthernBiotech	Cat# 2048-01	RRID: AB_2795685
His epitope tag peptide HHHHHH conjugated to KLH'	GenScript	Cat# A00174	RRID: AB_914703
Mouse anti V5-Tag antibody	Bio-Rad	Cat# MCA1360	RRID: AB_322378
R-Phycoerythrin AffiniPure® F(ab')$_2$ Fragment Goat Anti-Human IgG, Fcγ fragment specific	Jackson Immunoresearch	Cat# 109-116-170	RRID: AB_2337681
anti-C-Myc Antibody (Chicken) - FITC Conjugated	Immunolgy Consultants Lab	Cat# CMYC-45F	
Alexa Fluor® 647 AffiniPure® Goat Anti-Human IgG (H+L)	Jackson Immunoresearch	Cat# 109-605-003	RRID: AB_2337880
Alexa Fluor 647 streptavidin	Invitrogen	Cat# S21374	
BV421 streptavidin	BioLegend	Cat# 405225	
BV650 streptavidin	BioLegend	Cat# 405232	
eBioscience Fixable Viability Dye eFluor 506	Invitrogen	Cat# 65-0866-18	
eBioscience Fixable Viability Dye eFluor 780	Invitrogen	Cat# 65-0865-14	
mouse anti-human CD3 APC-Cy7 (SP34-2)	BD Biosciences	Cat# 557757	RRID: AB_396863
mouse anti-human CD4 APC-Cy7 (SK3)	BD Biosciences	Cat# 566913	RRID: AB_2739681
mouse anti-human CD8a APC-eFluor780 (RPA-T8)	Thermo Fisher Scientific	Cat# 47-0088-42	RRID: AB_1272046
mouse anti-human CD14 APC-Cy7 (M5E2)	BioLegend	Cat# 301820	RRID: AB_493695
mouse anti-human CD16 APC-eFluor780 (eBioCB16)	Thermo Fisher Scientific	Cat# 47-0168-42	RRID: AB_11220086
mouse anti-human CD20 BV570 (2H7)	BioLegend	Cat# 302332	RRID: AB_2563805
mouse anti-human CD20 PerCP-Cy5.5 (2H7)	BioLegend	Cat# 302326	RRID: AB_893283
mouse anti-human CD27 PE-Cy7 (O323)	BioLegend	Cat# 302838	RRID: AB_2561919
mouse anti-human CD38 APC (OKT10)	NHP Reagents	Cat# PR-3801	RRID: AB_2819277

mouse anti-human CD71 FITC (L01.1)	BD Biosciences	Cat# 347513	RRID: AB_400316
goat anti-human IgD FITC (polyclonal)	Southern Biotech	Cat# 2030-02	RRID: AB_2795624
mouse anti-human IgG PE-Cy7 (G18-145)	BD Biosciences	Cat# 561298	RRID: AB_10611712
mouse anti-human IgG BV786 (G18-145)	BD Biosciences	Cat# 564230	RRID: AB_2738684
mouse anti-human IgM PerCP-Cy5.5 (G20-127)	BD Biosciences	Cat# 561285	RRID: AB_10611998
mouse anti-human IgM BV605 (G20-127)	BD Biosciences	Cat# 562977	RRID: AB_2737928
TotalSeq-C0953 PE streptavidin	BioLegend	Cat# 405265	
TotalSeq-C0251 anti-human Hashtag 1 antibody	BioLegend	Cat# 394661	RRID: AB_2801031
TotalSeq-C0251 anti-human Hashtag 2 antibody	BioLegend	Cat# 394663	RRID: AB_2801032
TotalSeq-C0251 anti-human Hashtag 3 antibody	BioLegend	Cat# 394665	RRID: AB_2801033
TotalSeq-C0251 anti-human Hashtag 4 antibody	BioLegend	Cat# 394667	RRID: AB_2801034
TotalSeq-C0251 anti-human Hashtag 5 antibody	BioLegend	Cat# 394669	RRID: AB_2801035
TotalSeq-C0251 anti-human Hashtag 6 antibody	BioLegend	Cat# 394671	RRID: AB_2820042
TotalSeq-C0251 anti-human Hashtag 7 antibody	BioLegend	Cat# 394673	RRID: AB_2820043
TotalSeq-C0251 anti-human Hashtag 8 antibody	BioLegend	Cat# 394675	RRID: AB_2820044
TotalSeq-C0251 anti-human Hashtag 9 antibody	BioLegend	Cat# 394677	RRID: AB_2820045
TotalSeq-C0251 anti-human Hashtag 10 antibody	BioLegend	Cat# 394679	RRID: AB_2820046
